# Selective defluorination of 1,1,1,2-tetrafluoroethane by lithium phosphide reagents

**DOI:** 10.1039/d6dt00747c

**Published:** 2026-05-01

**Authors:** Hodan R. Warsame, Colleen M. Demetriou, Mark R. Crimmin

**Affiliations:** a Department of Chemistry, Molecular Sciences Research Hub, Imperial College London 82 Wood Lane Shepherds Bush London W12 0BZ UK m.crimmin@imperial.ac.uk

## Abstract

1,1,1,2-Tetrafluoroethane (HFC-134a), the most commonly used 3^rd^ generation refrigerant, reacts with lithium phosphide reagents through selective defluorophosphination of the single C(sp^3^)–F bond to yield diphenyl(2,2,2-trifluoroethyl)phosphane. DFT calculations were used to rationalise the most likely pathways and origin of selectivity. The new phosphane was demonstrated as a ligand for transition metals and its electronic and steric properties compared to structurally similar analogues.

## Abstract 2

1,1,1,2-Tetrafluoroethane (HFC-134a), the most commonly used 3^rd^ generation refrigerant, reacts with lithium phosphide reagents through selective defluorophosphination of the single C(sp^3^)–F bond to yield diphenyl(2,2,2-trifluoroethyl)phosphane.

## Graphical abstract

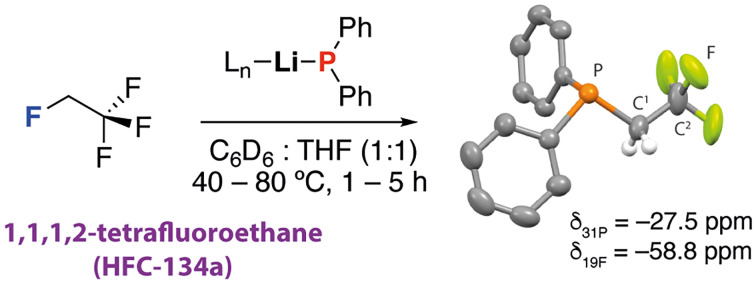

Hydrofluorocarbons (HFCs) are volatile fluorine containing gases that have been extensively used as third generation refrigerants.^1,2^ They tend to be non-toxic, non-flammable, and chemically inert gases that are readily condensed below room temperature.^3^ 1,1,1,2-Tetrafluoroethane (HFC-134a) has a boiling point of −26 °C and has been widely used as refrigerant in automobile air-conditioning units and as a propellent in metered dose inhalers. 1,1,1,2-Tetrafluoroethane has a global warming potential (GWP_100_) that is 1500 times greater than CO_2_.

Prior work investigating the reactivity of 1,1,1,2-tetrafluoroethane has established two reaction pathways (Fig. 1). The first, and most common, involves deprotonation with organometallic reagents followed by facile β-fluoride elimination to generate 1,1,2-trifluoroethene.^4^ For example, addition of alkyl lithium reagents to 1,1,1,2-tetrafluoroethane forms 1,1,2-trifluoroethene, in most cases as a reactive intermediate which undergoes a second deprotonation to generate the trifluorovinyl anion.^5–9^ Similarly, reactions with oxygen, nitrogen or sulfur based nucleophiles with 1,1,1,2-tetrafluoroethane in DMSO solution yield alkene containing products and are proposed to occur through an initial deprotonation.^10^ This deprotonation–elimination strategy has recently been used as a means to recycle the fluoride content of 1,1,1,2-tetrafluoroethane (and related HFCs) through treatment with KO^*t*^Bu or KHMDS to generate KF *in situ*.^11^ Given the Brønstead acidity of the protons adjacent to the multiple fluorine atoms in 1,1,1,2-tetrafluoroethane, it is perhaps expected that its dominant reaction pathway involves deprotonation. Very recently, we documented an alternative type of reactivity of this HFC involving a 1,2-defluorination to form 1,1-difluoroethene (Fig. 1).^12^ Reaction of 1,1,1,2-tetrafluoroethane with a reagent containing a [Mg–Mg] bond led to exclusive reaction of the C(sp^3^)–F bonds, with no evidence for deprotonation. This switch in selectivity was explained by the fluorophilic nature of the magnesium reagent and proposed to occur through an initial nucleophilic attack on the C(sp^3^)–F bond to generate an unstable organomagnesium intermediated followed by β-fluoride elimination.

**Fig. 1:**
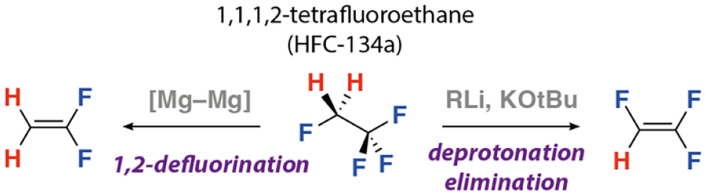
Reactions of 1,1,1,2-tetrafluoroethane.

Based on this finding, and as part of ongoing studies into the remediation and chemical recycling of refrigerants,^13^ we became interested in whether highly nucleophilic but non-basic reagents might react with 1,1,1,2-tetrafluoroethane in pathways that avoid deprotonation events. Herein we report that lithium diphenylphosphide compounds selectively defluorinate 1,1,1,2-tetrafluoroethane with nucleophilic displacement of the C(sp^3^)–F bond, leaving the CF_3_ group chemically unaltered. We use DFT calculations to rationalise this discovery and demonstrate that the product of defluorination, a trifluoroethyl substituted phosphane, is a versatile ligand for transition metals.

The reaction of 1,1,1,2-tetrafluoroethane (HFC-134a, 1 bar) with the lithium diphenylphosphide 1·TMEDA in a 1 : 1 mixture of C_6_D_6_ (or toluene) : THF for 5 h at 40 °C led to the selective formation of diphenyl(2,2,2-trifluoroethyl)phosphane 2 which was isolated in 30% yield (59% *in situ*) as a colourless oil following column chromatography (Scheme 1). The reaction to form 2 is particularly noteworthy as this is the first example of the selective C(sp^3^)–F functionalisation of this HFC and the only one that generates a product with an intact trifluoromethyl group. All reactions known to date destroy this functionality through elimination pathways. Monitoring reactions by ^19^F NMR spectroscopy showed no formation of 1,1-difluoroethane or 1,1,2-trifluoroethane, the expected products from elimination.

**Scheme 1:**
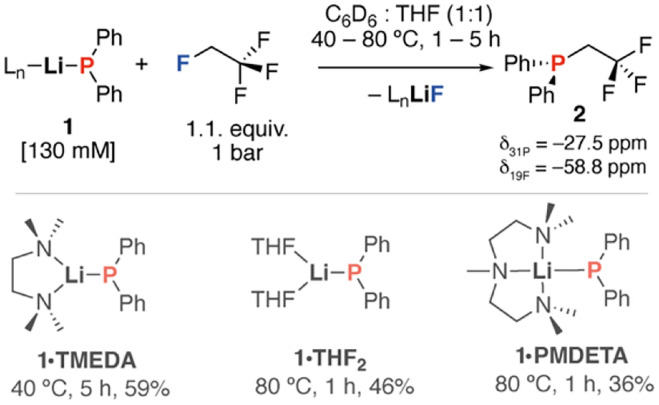
Reaction of 1,1,1,2-tetrafluoroethane with lithium phosphide reagents (1) to form phosphane 2.

The conditions and stoichiometry of this reaction were varied but the *in situ* yield was consistently between 40–60%, suggesting the modest isolated yield reflects the efficiency of the transformation. Variation of the ligand to include both 1·PMDETA and 1·THF_2_ consistently gave 2 in 36–46% yield. Optimisation of the concentrations, temperature, and equivalents of HFC showed that 0.13 M solutions of 1·TMEDA, 1.1 equiv. of 1,1,1,2-tetrafluoroethane and 40 °C gave the highest *in situ* yield of 2 of 59%. Attempts to modify the solvent away from binary mixture of hydrocarbon and THF gave poorer results with low yields observed when THF was used as a solvent alone. Some of the side-products of the reaction were determined by ^31^P NMR spectroscopy and include HPPh_2_, PPh_3_ and Ph_2_PPPh_2_. The only fluorine-containing species present in solution other than 2 at the end of the reaction was unreacted HFC-134a. 2 was characterised by a diagnostic methylene resonance in the ^1^H NMR spectrum found at *δ* = 2.91 ppm (2H, dq, ^3^*J*_F–H_ = 11.5 Hz, ^2^*J*_P–H_ = 0.8 Hz) with coupling to adjacent ^19^F and ^31^P nuclei. The ^19^F NMR spectrum shows a upfield resonance for the intact CF_3_ moiety at *δ* = −58.8 ppm (dt, ^3^*J*_P–F_ = 14.8 Hz, ^3^*J*_F–H_ = 11.5 Hz). With the ^31^P{^1^H} NMR exhibiting the expected quartet at *δ* = −27.5 ppm (q, ^3^*J*_P–F_ = 14.8 Hz). 2 could be crystallised from *n*-pentane solution at −35 °C. In the solid-state, 2 exists in a staggered conformation, with the CF_3_ group adjacent (*gauche*) to the lone pair on phosphorus. The C^1^–P bond length is 1.856(2) Å, while the C^1^–C^2^ bond length is 1.495(3) Å (Fig. 2).

**Fig. 2:**
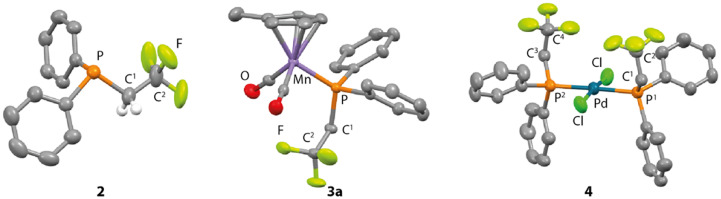
Crystal structures of 2, 3a, and 4. Selected hydrogen atoms omitted for clarity.

DFT calculations were undertaken to better understand the selective formation of 2. A series of potential reaction pathways were investigated for the reaction of 1·PMDETA with 1,1,1,2-tetrafluoroethane (Fig. 3). This nucleophile was chosen to reduce ambiguity over the coordination environment at lithium as 1·PMDETA was assumed to remain tetracoordinate in solution. We have previously studied the solution dynamics of closely related species and concluded that PMDETA remains coordinated and the complexes are monomeric in hydrocarbon solutions.^14^ The lowest energy pathway calculated in benzene solvent (PCM) involves the association of 1·PMDETA and 1,1,1,2-tetrafluoroethane to form an encounter complex Int-1 (
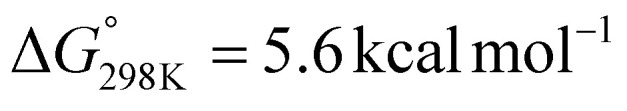
) in which the C(sp^3^)–F group of the HFC is weakly associated with the lithium site. Defluorophosphination evolves from Int-1 through TS-1 (Δ*G*^‡^_298 K_ = 26.3 kcal mol^−1^) and involves a σ-bond metathesis reaction which breaks the Li–P and C–F bond and simultaneously makes the C–P and Li–F bonds to form 2 and LiF·PMDETA. The overall process is exergonic (
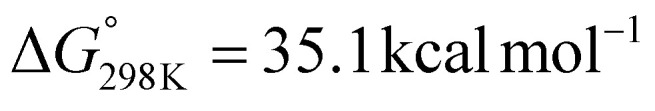
).

**Fig. 3:**
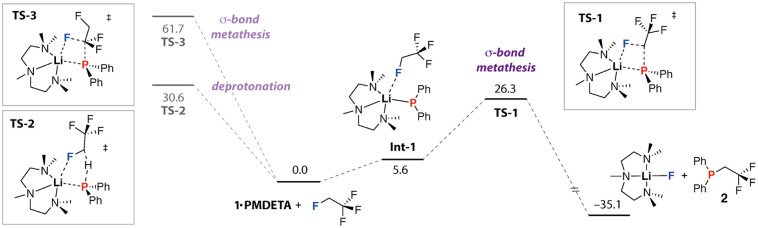
DFT calculated pathway for reaction of 1·PMDETA with 1,1,1,2-tetrafluoroethane through σ-bond metathesis and deprotonation. G09. B3PW91-D3/def2-TZVPP/PCM(benzene)//B3PW91-D3/def2-SVP (C,H)/def2-TZVP (Li,N,F,P)/PCM (benzene). Gibbs energies reported in kcal mol^−1^ at 298 K.

The selective formation of 2 is remarkable, as it implies that 1·PMDETA reacts with 1,1,1,2-tetrafluoroethane exclusively as a nucleophile, rather than a base, and shows a preference for the C(sp^3^)–F bond over the CF_3_ group. DFT calculations support this hypothesis. Pathways were located for the deprotonation of 1,1,1,2-tetrafluoroethane with 1·PMDETA through TS-2 (Δ*G*^‡^_298 K_ = 30.6 kcal mol^−1^) and σ-bond metathesis involving the CF_3_ group *via*TS-3 (Δ*G*^‡^_298 K_ = 61.7 kcal mol^−1^). Both are higher in energy than the experimentally observed product formed through TS-1. An alternative pathway involving addition of the C–F bond of the HFC to the phosphorus site of **2** to form a hypervalent centre was also found to be significantly higher in energy (ΔG^‡^_298 K_ > 45 kcal mol^−1^) and inaccessible under the experimental conditions. Changing the solvent model to THF, there is a stabilisation of **TS-2** (Δ*G*^‡^_298 K_ = 27.1 kcal mol^−1^) relative to **TS-1** (ΔG^‡^_298 K_ = 26.9 kcal mol^−1^) such that deprotonation would expect to be competitive with nucleophilic substitution. This finding is consistent with experimental observation that the reaction proceeds with lower efficiency (27% yield) in THF and the need for a solvent mixture with a reduced dielectric constant. For comparison, a series of s-block reagents including metal amides and alkyls react with 1,1,1,2-tetrafluoroethane through deprotonation and elimination pathways.^4–11^ This contrasting behaviour is likely a reflection of the increased polarisability of the phosphorus atom in 1·PMDETA in comparison to nitrogen or carbon rendering it a better nucleophile and poorer base.

Established synthetic routes to 2 involve the trifluoroethylation of diphenylphosphine with suitably electrophilic reagents including trifluoroethyl iodides and triflates.^15,16^ While 2 has been employed as a reagent in photoredox hydroalkylation of alkenes,^17^ there are no reports of its use as a ligand, despite the potential that electronically modified phosphines hold for coordination chemistry and catalysis. The reaction of 2 with a small array of transition metal precursors under both thermal and photochemical conditions was investigated (Scheme 2). These reactions allowed the preparation of the metal carbonyl complexes 3a–c, along with the square planar d^8^ palladium(ii) complex 4.

**Scheme 2:**
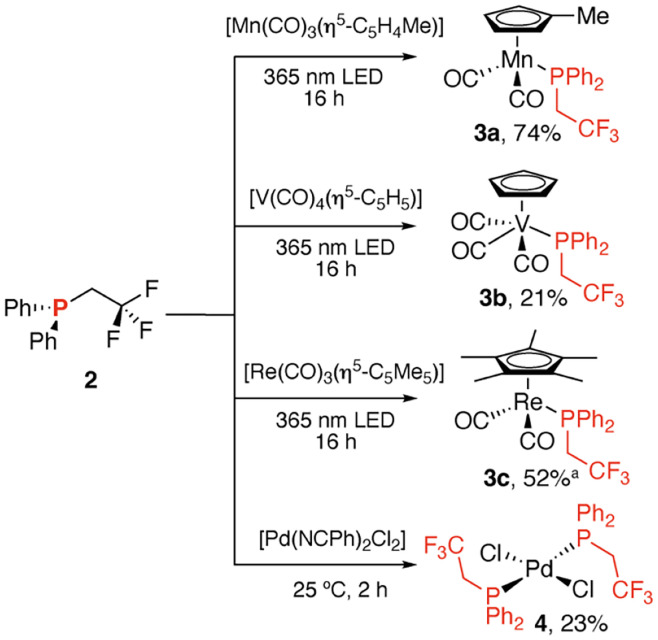
Reaction of 2 with transition metal complexes to form 3a–c and 4. ^a^3c formed alongside [Re(CO)(PPh_2_CH_2_CF_3_)_2_(η^5^-C_5_Me_5_)] (3c′) in a 7.7 : 1 ratio.

Substitution of carbonyl ligands with 2 occurred readily on irradiation with a 365 nm LED. While in the case of 3a and 3b monosubstituted products were formed cleanly, the rhenium complex 3c was generated alongside a minor isomer 3c′ (7.7 : 1 ratio 3c : 3c′) likely to be the bis(phosphine) complex formed from substitution with 2 equiv. of 2. Similarly, 4 could be formed from thermal displacement of benzonitrile from [Pd(NCPh)_2_Cl_2_] with 2 equiv. of 2. ^19^F NMR spectroscopic data on 3a–c and 4 reveal that the fluorine chemical shift is largely insensitive to the coordination environment occurring between *δ* = −54.1 and −54.9 ppm. In contrast the ^31^P NMR resonance is entirely dependent on the nature of the metal and coligands. 3a and 4 were crystallographically characterised (Fig. 2).

Generation of this series of complexes provides an opportunity to interrogate the steric and electronic coordination properties of 2. The solid-state structure of 3a was compared directly to structurally characterised analogues with more common phosphine ligands.^16,18–22^ Comparison of buried volumes (%*V*_bur_) for 2, PPh_2_CH_2_Ph, PPh_2_Me, and PMe_3_ gave values of %*V*_bur_ = 30.3, 29.0, 26.4 and 23.8% respectively, suggesting that 2 occupies a similar steric volume to other diphenylalkylphosphanes. Symmetric and asymmetric carbonyl stretches of 3a occur at *ν*_CO_ = 1925 and 1856 cm^−1^ and are comparable to those found in [Mn(CO)_2_(PPh_3_)(η^5^-C_5_H_4_Me)] of *ν*_CO_ = 1927 and 1863 cm^−1^ and [Mn(CO)_2_(PPMe_3_)(η^5^-C_5_H_4_Me)] of *ν*_CO_ = 1915 and 1856 cm^−1^,^23^ signifying these vibrations are reasonably insensitive to electronic changes at the phosphine. Further insight was gained through oxidation of 2 with Se_8_ to form the selenide 2·Se*in situ*. The ^1^*J*_Se–P_ coupling constant of 662 Hz compares well with that of 725 Hz reported for Se=PPh_2_Me and signifies that 2 is a slightly weaker σ-donating ligand than PPh_2_Me. Brisdon and others have reported a series of electron-neutral phosphine ligands containing perfluorocarbon groups including PPh_2_(CF=CF_2_) and PPh_2_[CF(CF_3_)_2_] with ^1^*J*_Se–P_ values of 785 and 828 Hz for the corresponding phosphide selenides.^21^

In summary, we report a new defluorophosphination reaction of 1,1,1,2-tetrafluoroethane (HFC-134a) with lithium diphenylphoshide reagents that proceed through selective functionalisation of the C(sp^3^)–F bond to form diphenyl(2,2,2-trifluoroethyl)phosphane. The coordination chemistry of the diphenyl(2,2,2-trifluoroethyl)phosphane has also been investigated. The low basicity and high nucleophilicity of the phosphide reagent likely control the chemoselectivity of this reaction and render reaction at the C(sp^3^)–F bond through a σ-bond metathesis pathway more energetically favourable than deprotonation of the adjacent C(sp^3^)–H bonds. This discovery may well allow design of new selective reactions of 1,1,1,2-tetrafluoroethane, and related HFC refrigerants, through tuning of nucleophility *vs.* basicity of the reagents involved.

## Conflicts of interest

There are no conflicts to declare.

## Supplementary Material

DT-055-D6DT00747C-s001

DT-055-D6DT00747C-s002

DT-055-D6DT00747C-s003

## Data Availability

Supplementary information (SI): experimental procedures, calculations, and spectroscopic data (PDF); cartesian coordinates for DFT calculated stationary points (XYZ). See DOI: https://doi.org/10.1039/d6dt00747c. CCDC 2536808 (2), 2536809 (3a) and 2537000 (4) contain the supplementary crystallographic data for this paper.^24*a*–*c*^
