# Supplementary material for: Selective defluorination of 1,1,1,2-tetrafluoroethane by lithium phosphide reagents
Source: Dalton Trans. 2026 May 1;55(20):7826–30. doi: 10.1039/d6dt00747c (PMC13162326; doi:10.1039/d6dt00747c)
Supplement: DT-055-D6DT00747C-s002 [file DT-055-D6DT00747C-s002.pdf]

Supplementary Information (SI) for Dalton Transactions.
This journal is © The Royal Society of Chemistry 2026

Selective Defluorination of 1,1,1,2-Tetrafluoroethane by Lithium Phosphide Reagents

Supplementary Information

Hodan R. Warsame, Colleen M. Demetriou, Mark R. Crimmin*

m.crimmin@imperial.ac.uk

Department of Chemistry, Molecular Sciences Research Hub, 82 Wood Lane, Shepherds Bush, London, W12 0BZ, UK

Contents:

1. General Procedures 3

2.1 Synthesis of Lithium Phosphide Reagents 4

3.1 General Procedure of Batch Scale Reactions of HFC-134a with Lithium Phosphide Reagents 7

3.2 Optimisation of Batch Scale Reactions of HFC-134a with Lithium Phosphide Reagents 7

4.1 Batch Synthesis of diphenyl(2,2,2-trifluoroethyl)phosphane, 2 10

4.2 Batch Synthesis of diphenyl(2,2,2-trifluoroethyl)phosphine oxide, 2a 11

4.3 *In-Situ* Formation of Phosphine Selenide, 2·Se 12

5.1 Coordination of 2 to Transition Metal Complexes 13

5.2 Buried Volumes of $Mn(CO)_2CpPR_3$ Complexes 17

6.1 Single Crystal X-ray Diffraction Data 18

7.1 Computational Studies 21

7.2 DFT Study: Experimental Mechanism 22

7.3 DFT Study: Alternative Mechanisms 23

7.4 DFT Study: Alternative Products 25

7.5 Functional Testing 27

7.6 NBO Analysis 28

8.1 Computational Coordinates 30

9.1 IR Spectra 56

10.1 NMR Spectra 59

11.1 References 77

1. General Procedures

Unless otherwise specified, standard Schlenk line and glovebox techniques were used for all manipulations under an inert atmosphere of nitrogen or argon. NMR scale reactions were performed in J. Young NMR tubes equipped with internal standard of fluorobenzene was used (^{19}F NMR spectroscopy) and prepared in a glovebox.

An MBraun Labmaster glovebox was used, operating at 0.1 ppm H_2O and O_2. Glassware was dried for at least 6h at 120 ºC prior to use. Benzene-d_6, toluene, diethyl ether, *n*-pentane, *n*-hexane, and THF were obtained from a Grubbs design solvent purification system and de-gassed and stored over 3 Å molecular sieves before use. All reagents were acquired from Sigma Aldrich (Merck), Tokyo Chemical Industry, Scientific Laboratory Supplies, or Fluorochem and used without further purification unless specified. 1,1,1,2-tetrafluoroethane (HCF-134a) was acquired from CK special gases and used without further purification or drying.

Where liquids at 25 °C, reagents were dried over activated 3 Å molecular sieves and freeze-pump-thaw degassed prior to use. *N*,*N*,*N'*,*N'*-tetramethylethylenediamine (TMEDA) and *N*,*N*,*N'*,*N''*,*N''*-pentamethyldiethylenetriamine (PMDETA) were distilled over CaH_2 and dried over activated 3 Å molecular sieves and freeze-pump-thaw degassed prior to use.

Purifications were carried out by column chromatography on silica gel (tech grades, 60 Å, 230-400 mesh, 40-63 µm particle size). AT-IR spectra were recorded on an Agilent Technologies Cary 630 FTIR spectrometer.

2.1 Synthesis of Lithium Phosphide Reagents

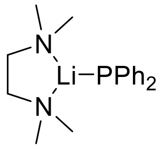

Synthesis of 1·TMEDA

In a N_2 filled glovebox, TMEDA (2.4 mL, 16.1 mmol, 1 equiv.) was dissolved in *n*-pentane (25 mL) and transferred into a Schlenk. Diphenylphosphine (2.8 mL, 16.1 mmol, 1 equiv.) was added dropwise to the Schlenk at room temperature *via* syringe. At – 78 °C, a 2.5 M solution of *n*-BuLi in *n*-hexane (7.5 mL, 17.7 mmol, 1.1 equiv.) was added dropwise to the reaction mixture, forming a bright yellow solution. The reaction mixture was left to thaw to room temperature and stirred for 1.5 h. The solution was filtered *via* cannula filtration, and the yellow solid was dried under *vacuo*, affording a bright yellow powder (4.57 g, 14.8 mmol, 92 %).

^{1}H NMR (400 MHz, C_6D_6) δ/ppm: 7.96 (d, *J* = 7.6 Hz, 4H, C$\mathbf{H}_{\mathbf{Ph}}$), 7.21 (t, *J* = 7.5 Hz, 4H, C$\mathbf{H}_{\mathbf{Ph}}$), 6.95 (t, *J* = 7.2 Hz, 2H, C$\mathbf{H}_{\mathbf{Ph}}$), 1.90 (s, 12H, NC$\mathbf{H_3}$), 1.69 (s, 4H, NC$\mathbf{H_2}$).

^{13}C NMR (101 MHz, C_6D_6) δ/ppm: 151.8 (s, CH_{Ph}), 131.1 (s, **C**H_{Ph}), 127.6 (d, *J* = 23.8 Hz, **C**H_{Ph}), 121.7 (s, **C**H_{Ph}), 57.3 (s, N**C**H_2), 46.0 (s, N**C**H_3).

^{31}P{^{1}H} NMR (162 MHz, C_6D_6) δ/ppm: -31.0 (s).

^{7}Li NMR (156 MHz, C_6D_6) δ/ppm: 1.8 (s).

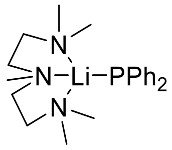

Synthesis of 1·PMDETA

In a N_2 filled glovebox, PMDETA (0.6 mL, 2.87 mmol, 1 equiv.) was dissolved in *n*-hexane (15 mL) and transferred into a Schlenk. Diphenylphosphine (0.5 mL, 2.87 mmol, 1 equiv.) was added dropwise to the Schlenk at room temperature *via* syringe. At – 78 °C, a 1.6 M solution of *n*-BuLi in *n*-hexane (2.0 mL, 3.16 mmol, 1.1 equiv.) was added dropwise to the reaction mixture, forming a bright yellow solution. The reaction mixture was left to thaw to room temperature and stirred for 1.5 h. The solution was filtered *via* cannula filtration, and the yellow solid was dried under *vacuo*, affording a bright yellow powder (0.52 g, 1.43 mmol, 50 %).

^{1}H NMR (400 MHz, C_6D_6) δ/ppm: 7.91 (tt, *J* = 6.2, 1.4 Hz, 4H, C**H**$_{\mathbf{Ph}}$), 7.22 – 7.13 (m, 4H, C**H**$_{\mathbf{Ph}}$), 6.91 (tt, *J* = 7.2, 1.3 Hz, 2H, C**H**$_{\mathbf{Ph}}$), 1.95 – 1.74 (m, 15H, NC**H**$_{\mathbf{3}}$), 1.67 – 1.53 (m, 8H. NC**H**$_{\mathbf{2}}$).

^{13}C NMR (101 MHz, C_6D_6) δ/ppm: 155.8 (d, *J* = 42.8 Hz, CH$_{Ph}$), 130.9 (d, *J* = 17.7 Hz, **C**H$_{Ph}$), 128.0 (s, **C**H$_{Ph}$), 120.5 (s, **C**H$_{Ph}$), 57.4 (s, N**C**H$_2$), 53.6 (s, N**C**H$_2$), 46.0 (s, N(**C**H$_3$)$_2$), 44.9 (s, N**C**H$_3$).

^{31}P{^{1}H} NMR (162 MHz, C_6D_6) δ/ppm: -23.3 (s).

^{7}Li NMR (156 MHz, C_6D_6) δ/ppm: 1.3 (s).

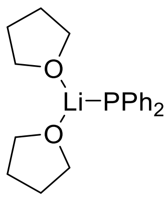

Synthesis of 1·(THF)$_2$

In a N_2 filled glovebox, THF (0.7 mL, 8.61 mmol, 3 equiv.) was dissolved in 15 mL of *n*-hexane and transferred into a Schlenk. Diphenylphosphine (0.5 mL, 2.87 mmol, 1 equiv.) was then added to the Schlenk. At – 78 °C, a 1.6 M solution of *n*BuLi in *n*-hexane (1.98 mL, 3.16 mmol, 1.1 equiv.) was added dropwise to the reaction mixture, forming a bright yellow solution. The reaction was left to thaw to room temperature and stirred for 1.5 h. The solution was filtered *via* cannula filtration, and the yellow solid was dried under *vacuo*, affording a bright yellow powder (0.71 g, 2.12 mmol, 74 %).

^{1}H NMR (400 MHz, C_6D_6) δ/ppm: 7.92 (ddd, *J* = 8.0, 6.5, 1.4 Hz, 4H, C**H$_{Ph}$**), 7.22 – 7.13 (m, 4H, C**H$_{Ph}$**), 6.95 (tt, *J* = 7.3, 1.2 Hz, 2H, C**H$_{Ph}$**), 3.53 – 3.40 (m, 8H, C**H$_2$**O), 1.30 – 1.16 (m, 8H, C**H$_2$**).

^{13}C NMR (101 MHz, C_6D_6) δ/ppm: 151.7 (d, *J* = 30.7 Hz, **C**H$_{Ph}$), 132.2 (d, *J* = 15.5 Hz, **C**H$_{Ph}$), 128.1 (d, *J* = 5.8 Hz, **C**H$_{Ph}$), 122.3 (s, **C**H$_{Ph}$), 68.7 (s, **C**H$_2$O), 25.7 (s, **C**H$_2$).

^{31}P{^{1}H} NMR (162 MHz, C_6D_6) δ/ppm: -36.8 (s).

^{7}Li NMR (156 MHz, C_6D_6) δ/ppm: 1.85 (s).

3.1 General Procedure of Batch Scale Reactions of HFC-134a with Lithium Phosphide Reagents

In a N_2 filled glovebox, ***L*·LiPPh₂** (0.08 mmol, 1 equiv.) was dissolved in 0.6 mL of a 1:1 solution of C_6D_6/THF and transferred to a J Young NMR tube equipped with a known amount of fluorobenzene as an internal standard. The solution was degassed once via freeze-pump-thaw, and HFC-134a (1 bar, 25 °C, 0.088 mmol, 1.1 equiv.) was added to the J. Young NMR tube. The J. Young NMR tube was inverted several times and heated to 40 °C for 5 h. The reaction was followed by NMR spectroscopy and the yield of the product **2** was determined through *in situ* upon integral comparison to a known amount of fluorobenzene in the ^{19}F NMR spectrum.

3.2 Optimisation of Batch Scale Reactions of HFC-134a with Lithium Phosphide Reagents

- **Ligand Screen**

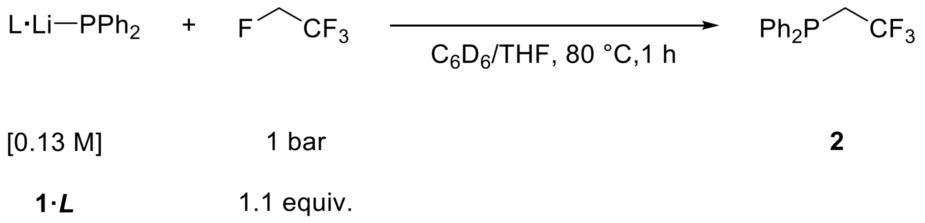

Ligand	Yield of 2 (%)[a]
TMEDA	46
PMDETA	36
THF	46

Table S1: Results of ligand variation. [a]Yield calculated by ^{19}F NMR spectroscopy using fluorobenzene and an internal standard.

- **Concentration Screen**

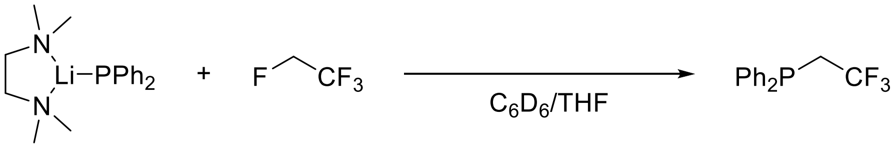

1·TMEDA 1 bar **2**

**Concentration of 1·TMEDA (M) {amount of 1·TMEDA (mmol)}**	**Approx. equivalent of HFC-134a (headspace of NMR tube = 2.2 mL)**	**Temperature (°C)**	**Yield of 2 (%)[a]**
0.02 {0.011}	7	25	51
0.04 {0.022}	4	40	55
0.10 {0.059}	1.5	40	63
0.13 {0.080}	1.1	25	53
0.13 {0.080}	1.1	40	59
0.13 {0.080}	1.1	60	48
0.13 {0.080}	1.1	80	51
0.18 {0.110}	0.8	25	46
0.18 {0.11}	0.8	40	62
0.29 {0.176}	0.5	40	57

Table S2: Effect of HFC-134a equivalence/concentration/temperature on **1·TMEDA**. [a]Yield calculated by ^{19}F NMR spectroscopy using fluorobenzene as an internal standard.

- **Solvent Screen**

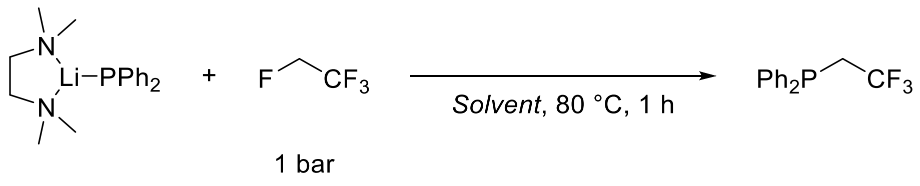

1·TMEDA 1.1 equiv. **2**

Solvent	Yield of 2 (%)[a]
C_6D_6/THF	46
Toluene/THF	40
THF	27

Table S3: Results of solvent screen. [a]Yield calculated by ^{19}F NMR spectroscopy using fluorobenzene as an internal standard.

4.1 Batch Synthesis of diphenyl(2,2,2-trifluoroethyl)phosphane, 2

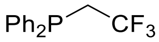

Preparative Procedure: In a N_2 filled glovebox, **1·TMEDA** (0.494 g, 1.60 mmol, 1 equiv.) was dissolved in 20 mL a 1:1 solution of Toluene/THF, to make a 0.08 M solution. This solution was transferred into an ampoule (headspace = 155 mL). The solution was degassed *via* freeze-pump-thaw, and HFC-134a (1 bar, 25 °C, 6.26 mmol, 3.9 equiv.) was added. The ampoule was sealed and the reaction mixture was heated to 40 °C for 5 h, a colour change from bright yellow to pale yellow was observed. The reaction mixture was concentrated *in vacuo* by rotary evaporation before being purified by an automated silica column, eluted with 100% *n*-pentane. The resulting oil was dissolved in a minimum volume of *n*-pentane and left to recrystalise for 3 days at –35 °C. The mother liquor was quickly decanted, and a colourless oil was isolated (129 mg, 0.024 mmol, 30 %).

^{1}H NMR (400 MHz, $CDCl_3$) δ/ppm: 7.49 – 7.40 (m, 4H, C$\mathbf{H}_{\mathbf{Ph}}$), 7.38 (dq, *J* = 4.7, 1.7 Hz, 6H, C$\mathbf{H}_{\mathbf{Ph}}$), 2.91 (qd, *J* = 11.6, 0.8 Hz, 2H, C$\mathbf{H_2}$)

^{13}C NMR (101 MHz, $CDCl_3$) δ/ppm: 136.9 (d, *J* = 11.1 Hz, **C**H_{Ph}), 133.2 (d, *J* = 20.6 Hz, **C**H_{Ph}), 129.8 (s, **C**H_{Ph}), 129.2 (d, *J* = 7.3 Hz, **C**H_{Ph}), 127.9 (q, *J* = 260.0 Hz, **C**F_3), 35.6 (qd, *J* = 27.7, 23.1 Hz, **C**H_2).

^{19}F NMR (377 MHz, $CDCl_3$) δ/ppm: -58.8 (dt, *J* = 14.8, 11.5 Hz).

^{31}P NMR (162 MHz, $CDCl_3$) δ/ppm: -27.5 (q, *J* = 13.9 Hz).

^{31}P{^{1}H} NMR (162 MHz, $CDCl_3$) δ/ppm: -27.5 (q, *J* = 13.9 Hz).

IR (thin film)/cm^{-1}: 3055, 3014, 2954, 1961, 1808, 1483, 1429, 1288, 1226, 1101, 1044.

4.2 Batch Synthesis of diphenyl(2,2,2-trifluoroethyl)phosphine oxide, 2·O

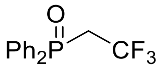

In a N_2 filled glovebox, **1·TMEDA** (0.171 g, 0.555 mmol, 1 equiv.) was dissolved in 5 mL of THF and transferred into an ampoule (headspace = 15 mL). The solution was degassed *via* freeze-pump-thaw and HFC-134a (1 bar, 25 °C, 0.605 mmol, 1.1 equiv.) was added to the ampoule at room temperature. The ampoule was sealed and the reaction mixture was heated to 50 °C for 5 h, a colour change from bright yellow to pale yellow was observed. Following cooling to room temperature, meta-Chloroperoxybenzoic acid, mCPBA, (0.308 g, 1.79 mmol, 3.2 equiv.) was added to the ampoule, and the solution went colourless immediately. The reaction was left to stir for one hour and the reaction mixture was concentrated *in vacuo* by rotary evaporation and the crude dissolved in 20 mL of DCM. The organic layer was washed with 2 x 20 mL $NaHSO_3$ and then 2 x 20 mL Na_2CO_3 and then dried with Mg_2SO_4. The organic layer was concentrated *in vacuo* by rotary evaporation before being purified by silica column chromatography, eluted with 80/20 ethyl acetate/*n*-pentane – 100% ethyl acetate gradient. A white solid was isolated (49.9 mg, 0.176 mmol, 32 %)

^{1}H NMR (400 MHz, $CDCl_3$) δ/ppm: 7.78 (ddd, *J* = 12.3, 8.3, 1.4 Hz, 4H, C**H**$_{Ph}$), 7.62 – 7.55 (m, 2H, C**H**$_{Ph}$), 7.54 – 7.45 (m, 4H, C**H**$_{Ph}$), 3.24 (dq, *J* = 11.9, 10.6 Hz, 2H, C**H**$_2$).

^{13}C NMR (101 MHz, $CDCl_3$) δ/ppm: 132.4 (d, *J* = 2.8 Hz), 131.4, 130.7 (d, *J* = 9.7 Hz), 128.7 (d, *J* = 12.6 Hz), 123.9 (qd, *J* = 277.4, 4.3 Hz), 36.5 (dq, *J* = 65.4, 29.1 Hz).

^{19}F NMR (377 MHz, $CDCl_3$) δ/ppm: -55.5 (dt, *J* = 10.6, 8.1 Hz).

^{19}F{^{1}H} NMR (377 MHz, $CDCl_3$) δ/ppm: -55.5 (d, *J* = 6.9 Hz).

^{31}P NMR (162 MHz, $CDCl_3$) δ/ppm: 22.2.

4.3 *In-Situ* Formation of Phosphine Selenide, **2**·Se

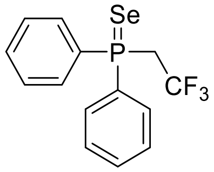

In a N_2 filled glovebox, **2** (38.5 mg, 0.144 mmol, 1 equiv.) and selenium (37.4 mg, 0.474 mmol, 3.3 equiv.) was dissolved in 0.6 mL of $CDCl_3$ and transferred to a J. Young NMR tube. The solution was heated to 60 °C for 4 hours. The NMR tube was cooled to room temperature and a ^{31}P NMR was acquired without further purification.

^{31}P NMR (162 MHz, $CDCl_3$) δ/ppm: 21.9 (d, $^{1}J_{P\text{-}Se}$ = 661.8 Hz).

5.1 Coordination of 2 to Transition Metal Complexes

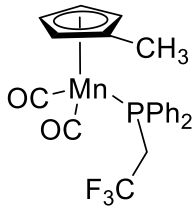

Synthesis of [Mn(CO)$_2$Cp'(PPh$_2$CH$_2$CF$_3$)], 3a

In a N_2 filled glovebox, **2** (116 mg, 0.434 mmol, 1 equiv.) and [Mn(CO)$_3$Cp'] (108 mg, 0.495 mmol, 1.1 equiv.) were dissolved in C_6D_6 and the reaction mixture was irradiated (365 nm UV) for 3 days. The reaction mixture was diluted with 2 mL of *n*-pentane and filtered three times and then concentrated *in vacuo*. The crude product was then redissolved in a 4 mL 1:1 mixture of HMDSO and *n*-pentane and, the mother liquor was filtered and left to recrystalise for a day at –35 °C, forming brownish yellow crystals of **4** suitable for X-ray diffraction (120 mg, 0.255 mmol, 74 %).

^{1}H NMR (400 MHz, C_6D_6) δ/ppm: 7.42 – 7.28 (m, 4H, C**H$_{Ph}$**), 7.04 – 6.93 (m, 6H, C**H$_{Ph}$**), 3.90 (m, 2H, **Cp'**), 3.78 (m, 2H, **Cp'**), 2.91 (qd, *J* = 11.0, 7.6 Hz, 2H, C**H$_2$**), 1.63 (s, 3H, C**H$_3$**).

^{13}C NMR (126 MHz, C_6D_6) δ/ppm: 232.5 (d, *J* = 23.9 Hz, **C**O), 138.3 (d, *J* = 39.3 Hz, **C**H$_{Ph}$), 132.3 (d, *J* = 10.7 Hz, **C**H$_{Ph}$), 130.1 (s, **C**H$_{Ph}$), 128.8 (s, **C**H$_{Ph}$), 126.0 (q, *J* = 277.6 Hz, **C**F$_3$), 99.3 (**Cp'**) 83.0 (**Cp'**), 82.6 (**Cp'**), 39.1 (qd, *J* = 27.6, 17.0 Hz, **C**H$_2$), 13.6 (s, **C**H$_3$).

^{19}F NMR (377 MHz, C_6D_6) δ/ppm: -54.7 (td, *J* = 11.0, 3.3 Hz, C**F$_3$**).

^{31}P NMR (162 MHz, C_6D_6) δ/ppm: 80.8 (s).

IR (thin film)/cm^{-1}: 3090, 2930, 1925 (**C=O**), 1856 (**C=O**), 1482, 1377, 1031, 833, 669.

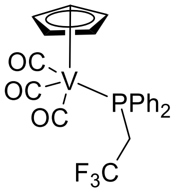

Synthesis of [$V(CO)_3Cp(PPh_2CH_2CF_3)$], 3b

In a N_2 filled glovebox, **2** (50 mg, 0.186 mmol, 1 equiv.) and [$V(CO)_4Cp$] (42.4 mg, 0.186 mmol, 1 equiv.) were dissolved in C_6D_6 and the reaction mixture was irradiated (365 nm UV) for 16 h. The reaction mixture was concentrated *in vacuo* and the crude product dissolved in 2 mL of *n*-pentane. The solution was filtered twice and then left in the freezer at – 35 °C for 1.5 h. The mother liquor was filtered, and a red solid was isolated and dried *in vacuo* (18 mg, 0.038 mmol, 21 %).

^{1}H NMR (400 MHz, C_6D_6) δ/ppm: 7.57 – 7.43 (m, 4H, C**H$_{Ph}$**), 7.25 – 7.10 (m, 6H, C**H$_{Ph}$**), 4.64 (d, *J* = 1.4 Hz, 5H, **Cp**), 3.14 (qd, *J* = 10.9, 6.3 Hz, 2H, C**H$_2$**).

^{13}C NMR (126 MHz, C_6D_6) δ/ppm: 135.8 (d, *J* = 34.3 Hz, **C**H$_{Ph}$), 132.4 (d, *J* = 10.0 Hz, **C**H$_{Ph}$), 130.6 (d, *J* = 2.2 Hz, **C**H$_{Ph}$), 128.9 (d, *J* = 9.2 Hz, **C**H$_{Ph}$), 125.6 (q, *J* = 278.0 Hz, **C**F$_3$), 91.8 (s, **Cp**), 36.6 (qd, *J* = 28.4, 11.9 Hz, **C**H$_2$).

^{19}F NMR (377 MHz, C_6D_6) δ/ppm: -54.5 (t, *J* = 10.9 Hz, C**F$_3$**).

^{31}P NMR (202 MHz, C_6D_6) δ/ppm: 76.1 (m).

IR (thin film)/cm^{-1}: 3053, 2274, 1949 (**C=O**), 1826 (**C=O**), 1569, 1431, 1409, 1295, 1236, 1107, 1060, 1012, 816, 740, 693.

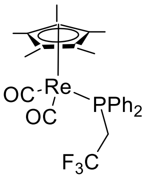

Synthesis of [Re(CO)$_3$Cp*(PPh$_2$CH$_2$CF$_3$)] 3c and [Re(CO)$_2$Cp*(PPh$_2$CH$_2$CF$_3$)$_2$], 3c'

In a N_2 filled glovebox, **2** (50 mg, 0.186 mmol, 1.1 equiv.) and [Re(CO)$_3$Cp*] (67.6 mg, 0.166 mmol, 1 equiv.) were dissolved in C_6D_6 and the reaction mixture was irradiated (365 nm UV) for 16 h. The reaction mixture was concentrated *in vacuo* and the crude product dissolved in 2 mL *n*-pentane, and then left in the freezer at – 35 °C for 1.5 h. As no product precipitated, this solution was filtered, concentrated *in vacuo*, dissolved in 2 mL of HMDSO, and then left in the freezer at – 35 °C for 1.5 h. A light blue solid was isolated and dried *in vacuo* (56 mg, 0.086 mmol, 52 % (**3c:3c'** = 7.4:1)).

Major Isomer, 3c:

^{1}H NMR (400 MHz, C_6D_6) δ/ppm: 7.47 (ddt, *J* = 11.2, 8.1, 1.4 Hz, 4H, C**H$_{Ph}$**), 7.08 – 6.93 (m, 6H, C**H$_{Ph}$**), 3.24 (qd, *J* = 10.9, 8.1 Hz, 2H, C**H$_2$**), 1.61 (s, 15H, C**H$_3$**).

^{13}C NMR (126 MHz, C_6D_6) δ/ppm: 205.2 (d, *J* = 7.9 Hz, **C**O), 132.4 (d, *J* = 11.8 Hz, **C**H$_{Ph}$), 129.6 (s, **C**H$_{Ph}$), 128.0 (s, **C**H$_{Ph}$), 128.0 (s, **C**H$_{Ph}$), 127.0 (q, *J* = 277.2 Hz, **C**F$_3$), 95.5 (s, **Cp***), 39.5 (m, **C**H$_2$), 9.9 (s, **C**H$_3$).

^{19}F NMR (377 MHz, C_6D_6) δ/ppm: -54.9 (td, *J* = 11.2, 4.4 Hz).

^{31}P NMR (162 MHz, C_6D_6) δ/ppm: 14.0 (s).

IR (thin film)/cm^{-1}: 3055, 2900, 2270, 2003, 1914 (**C=O**), 1847 (**C=O**), 1441, 1433, 1292, 1237, 1066, 1060, 808, 699, 693.

Minor Isomer. 3c' – partial data:

^{1}H NMR (400 MHz, C_6D_6) δ/ppm: 7.57 (ddd, *J* = 12.1, 8.1, 1.6 Hz, 6H), 2.63 – 2.46 (m, 4H), 1.69 (s, 15H).

^{13}C NMR (126 MHz, C_6D_6) δ/ppm: 198.2 (s, **C**O), 136.1 (d, *J* = 47.7 Hz, **C**H$_{Ph}$), 131.6 (d, *J* = 3.0 Hz. **C**H$_{Ph}$), 130.7 (d, *J* = 9.7 Hz, **C**H$_{Ph}$), 128.4 (d, *J* = 12.2 Hz, **C**H$_{Ph}$), 97.9 (s, **Cp***), 37.0 – 35.5 (m, **C**H$_2$), 10.0 (s, **C**H$_3$).

^{19}F NMR (377 MHz, C_6D_6) δ/ppm: -55.3 – -55.4 (m).

^{31}P NMR (162 MHz, C_6D_6) δ/ppm: 17.6 (s).

IR (thin film)/cm^{-1}: 3055, 2900, 2270, 2003, 1914 (**C=O**), 1847 (**C=O**), 1441, 1433, 1292, 1237, 1066, 1060, 808, 699, 693

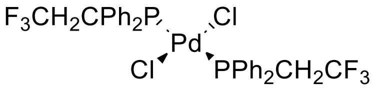

Synthesis of *trans*-[$PdCl_2(PPh_2CH_2CF_3)_2$], 4

In a N_2 filled glovebox, **2** (52.4 mg, 0.195 mmol, 1 equiv.) and [$Pd(NCPh)_2Cl_2$] (75 mg, 0.196 mmol, 1 equiv.) was dissolved in 10 mL of toluene and the reaction mixture was stirred for 2 h. The reaction mixture was concentrated *in vacuo,* and the crude product was redissolved in 10 mL of *n*-pentane, and an orange solid precipitated. The orange solid was isolated by filtration and dried *in vacuo* and then dissolved in 1 mL of chloroform. This solution was filtered and 2 mL of n-pentane added, at which point the orange solid precipitated. The solid was isolated by filtration and dried *in vacuo* to give **7** (31.8 mg, 0.0446 mmol, 23 %).

^{1}H NMR (400 MHz, $CDCl_3$) δ/ppm: 7.84 – 7.76 (m, 4H, C**H**$_{Ph}$), 7.67 – 7.56 (m, 2H, C**H**$_{Ph}$), 7.53 – 7.46 (m, 4H, C**H**$_{Ph}$), 3.61 (m, 2H, C**H**$_2$).

^{13}C NMR (126 MHz, $CDCl_3$) δ/ppm: 133.9 (d, J = 10.9 Hz, **C**H$_{Ph}$), 133.2 (s, **C**H$_{Ph}$), 129.4 (d, J = 12.4 Hz, **C**H$_{Ph}$), 124.7 (d, J = 60.3 Hz, **C**H$_{Ph}$), 122.0 (qd, J = 278.0, 7.0 Hz, **C**F$_3$). 33.2 – 31.8 (m, **C**H$_2$).

^{19}F NMR (377 MHz, $CDCl_3$) δ/ppm: -54.1 (dt, J = 14.3, 7.2 Hz).

^{31}P NMR (162 MHz, $CDCl_3$) δ/ppm: 16.9 (s).

5.2 Buried Volumes of $Mn(CO)_2CpPR_3$ Complexes

**CCDC No.**	**Compound**	**Buried Volume (%)**
GIXRIO[1]	$CpMn(CO)_2$**[Ph_2PCH_2Ph]**	29.0
XATJOS[2]	Cp’$Mn(CO)_2$**[PPh_2Me]**	26.4
DECBES[3]	$CpMn(CO)_2$**[PPr_2Me]**	27.9
VIPGIK[4]	Cp*$Mn(CO)_2$**[PMe_3]**	23.8
**3a**	Cp’$Mn(CO)_2$**[$PPh_2CH_2CF_3$]**	30.3

Table S4: Buried volumes of Mn complexes calculated using SambVca 2.1.[5]

6.1 Single Crystal X-ray Diffraction Data

compound	2	3a	4
**CCDC number**	2536808	2536809	2537000
**formula**	$C_{14}H_{12}F_3P$	$C_{22}H_{19}F_3MnO_2P$	$C_{28}H_{24}Cl_2F_6P_2Pd$
**formula weight (g·mol^{-1})**	268.21	458.28	713.71
**colour, habit**	colourless blocky needle	clear yellow column	yellow block
**crystal size (mm)**	0.43 x 0.25 x 0.09	0.396 x 0.261 x 0.175	0.19 x 0.12 x 0.09
**crystal system**	monoclinic	monoclinic	trigonal
**space group**	$P2_1/c$ (no. 14)	$P2_1/c$ (no. 14)	$P3_121$ (no. 152)
a **(Å)**	12.8168(3)	9.36076(14)	10.5520(2)
b **(Å)**	5.49400(10)	13.91716(19)	10.5520(2)
c **(Å)**	18.5190(3)	16.2618(3)	22.3322(4)
α **(°)**	90	90.0	90
β **(°)**	100.921(2)	106.1445(17)	90
γ **(°)**	90	90.0	120
**V (Å^3)**	1280.41(4)	2034.97(5)	2153.43(9)
**Z**	4	4	3 [b]
**T (K)**	173(1)	173.05(10)	173.05(10)
**D_c (g·cm^{-3})**	1.391	1.496	1.651
**radiation used, μ (mm^{-1})**	Cu Kα, 2.074	Mo Kα, 0.770	Cu Kα, 8.493
**F(000)**	552	936	1068
**absorption correction**	analytical	analytical	analytical
**min-max transmission**	0.518 - 0.843	0.791 - 0.906	0.396 - 0.593
**Θ range for data collection (°)**	3.512 - 73.781	2.608 - 29.426	4.839 - 68.270
**no. of unique reflns measured, obs [F > 4σ(F)]**	2566, 2251	5188, 4354	2620, 2556
**R_{int}, R_{sigma}**	0.0503, 0.0255	0.0309, 0.0240	0.0376, 0.0248
**completeness to Θ(full) (°)**	1.000 to 67.684	0.999 to 25.242	1.000 to 67.684
**no. of parameters, restraints**	163, 0	263, 0	177, 0
**R_1, wR_2 [F > 4σ(F)] [a]**	0.0381, 0.0983	0.0305, 0.0695	0.0349, 0.0922
**R_1, wR_2 [all data] [a]**	0.0435, 0.1042	0.0418, 0.0752	0.0356, 0.0929
**GooF**	1.045	1.053	1.038
**largest diff. Fourier peak, hole (eÅ^{-3})**	0.244, -0.271	0.298, -0.301	0.818, -0.392

Table S5: Crystal Data, Data Collection and Refinement Parameters. Data were collected using Xcalibur PX Ultra A (Cu radiation, compound **2**), Agilent Xcalibur 3 E Agilent (Mo radiation, compounds **3a**), and Rigaku XtaLAB Synergy-*i* diffractometers (Cu radiation, compound **4**). Raw frame data was reduced using CrysAlisPro,[6] and the structures were solved and refined using the OLEX2,[7] SHELXT,[8] and SHELX-2018[9] program systems. [a] $R_1 = \Sigma||F_o| - |F_c||/\Sigma|F_o|$; $wR_2 = \{\Sigma[w(F_o^2 - F_c^2)^2] / \Sigma[w(F_o^2)^2]\}^{1/2}$; $w^{-1} = \sigma_2(F_o^2) + (aP)^2 + bP$. [b] The asymmetric unit contains half of the main molecule.

The crystal structure of **2**

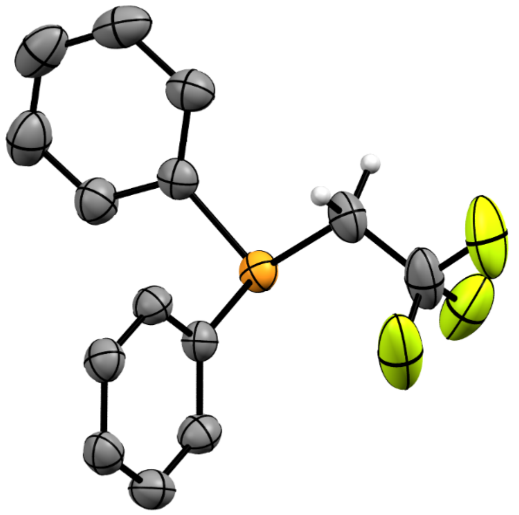

Figure S1: X-ray structure of **2**. Most hydrogen atoms are hidden for clarity.

The compound is a liquid at room temperature with crystals melting rapidly well below room temperature, necessitating appropriate low-temperature handling techniques. In the case of this sample, the microscope slide was placed on a petri dish containing dry ice before introducing the crystals suspended in X-ray oil. To help minimise water condensation an N_2 stream was aimed at the setup using an inverted funnel.

The crystal was found to be a well-separated two-component twin. The best model came from not modelling the twinning.

The crystal structure of **3a**

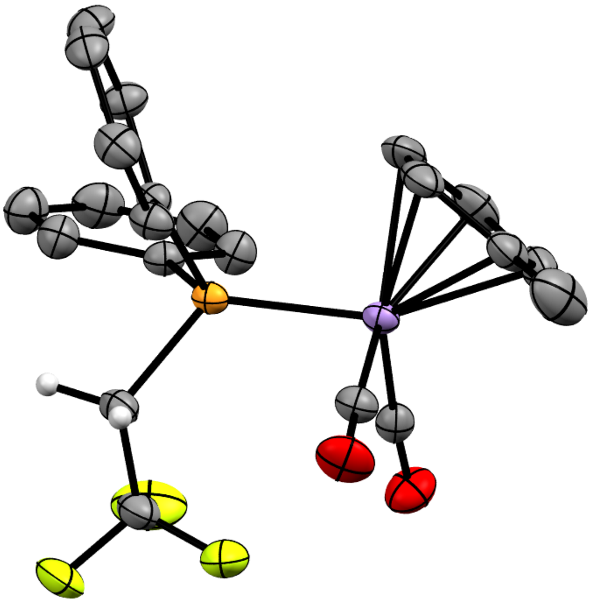

Figure S2: X-ray structure of **3a**. Most hydrogen atoms are hidden for clarity.

The crystal structure of **4**

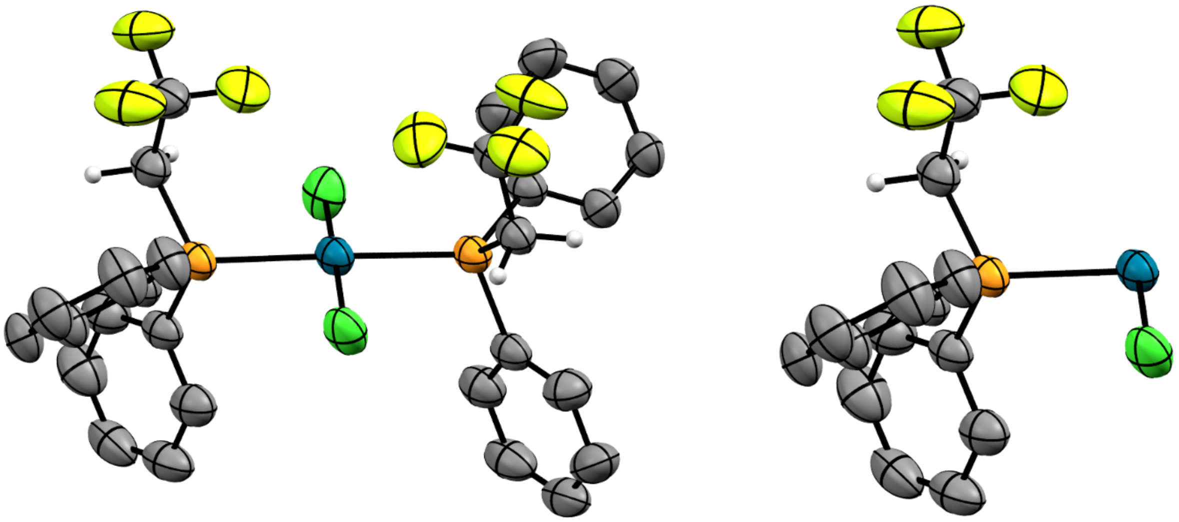

Figure S3: X-ray structure of **4**. Most hydrogen atoms are hidden for clarity. Left: whole molecule, right: asymmetric unit only.

The molecule of **4** lies on a crystallographic special position with the Pd atom being located on a C_2 axis. Thus, the Pd atom was modelled at half occupancy, with half the molecule in the asymmetric unit.

7.1 Computational Studies

DFT calculations were run using Gaussian 09 (Revision D.01) using the B3PW91 density functional including Grimme's D3 dispersion correction.[10-14] The def2-SVP(C,H)/def2-TZVP(Li,N,F,P) was used as a hybrid basis set.[15, 16] Solvent and dispersion effects were included in the optimisation process. Solvent effects were treated by the polarised continuum model (PCM) with a dielectric constant of 2.2706 (benzene), Geometry optimisation calculations were performed without symmetry constraints. The Gaussian 09 default optimisation criteria was tightened to 10^{-9} on the density matrix and 10^{-7} on the energy matrix. The default numerical integration grid was also enhanced using a pruned grid with 99 radial shells and 590 angular points per shell. Frequency analyses for all stationary points were performed using the enhanced criteria to confirm the nature of the structures as either minima (no imaginary frequency) or transition states (only one imaginary frequency). Intrinsic reaction coordinate (IRC) calculations followed by full geometry optimisations on final points were used to connect transition states and minima located on the potential energy surface allowing a full energy profile (calculated at 298.15 K, 1 atm) to be assembled.[17, 18 19] The graphical user interface used to visualise the structures of the intermediates and transition states was GaussView 5.0.9.

Single point energy calculations were performed using Gaussian 09 (Revision D.01) using an ultrafine integration grid on the optimised geometries. The B3PW91-D3 functional with def2-TZVPP basis set was used along with the PCM solvent correction for benzene. Unless otherwise stated, all orbital analyses were performed using densities calculated at this level of theory. The free energies were then recomputed using GoodVibes (v3.2) using the following settings: c = 0.13 M; T = 298.15 K; v = 0.972; entropy: Grimme damping on RRHO, cutoff = 100 cm^{-1} ; enthalpy: Head-Gordon q-RRHO, cutoff = 100 cm^{-1} ; symmetry corrections enabled.[19-21]

Functional testing was performed using the ωB97X-D, M06-2X, and M06-L functionals. The geometries were optimised on the B3PW91/def2-SVP(C,H)/def2-TZVP(Li,N,F,P)/ PCM level, and including Grimme's D3 dispersion correction, and implicit solvation was modelled using PCM method (benzene).[22]

A full NBO analysis was carried out and the relevant NPA charges and Wiberg Bond Indices were calculated. NBO analysis was performed using NBO 6.0 (using Gaussian 09 Rev D.01).[23]

7.2 DFT Study: Experimental Mechanism

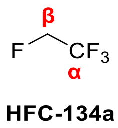

Figure S4: Drawn structure of HFC-134a with α- and β-carbons labelled.

Density functional theory (DFT) calculations were performed to determine the reaction mechanism for the defluorination of HFC-134a. Using ORCA to run Nudged Elastic Band with TS optimization (NEB-TS), it was found that the reaction mechanism proceeds *via* a σ-bond metathesis step.[24, 25] Results suggest that the α-fluorine in HFC-134a interacts with the Li in **1·PMDETA** through **Int-2** (ΔG^{o}_{298K} = 5.6 kcal/mol). Subsequently, the PPh_2 moiety in **1·PMDETA** approaches the α-carbon of HFC-134a *via* **TS-1** ($\Delta G_1^{\ddagger}{}_{298K}$ = 26.3 kcal/mol) undergoing σ-bond metathesis leading to the formation of the experimentally observed product, **2**, alongside **LiF·PMDETA** as a side-product (ΔG^{o}_{298K} = - 35.1 kcal/mol).

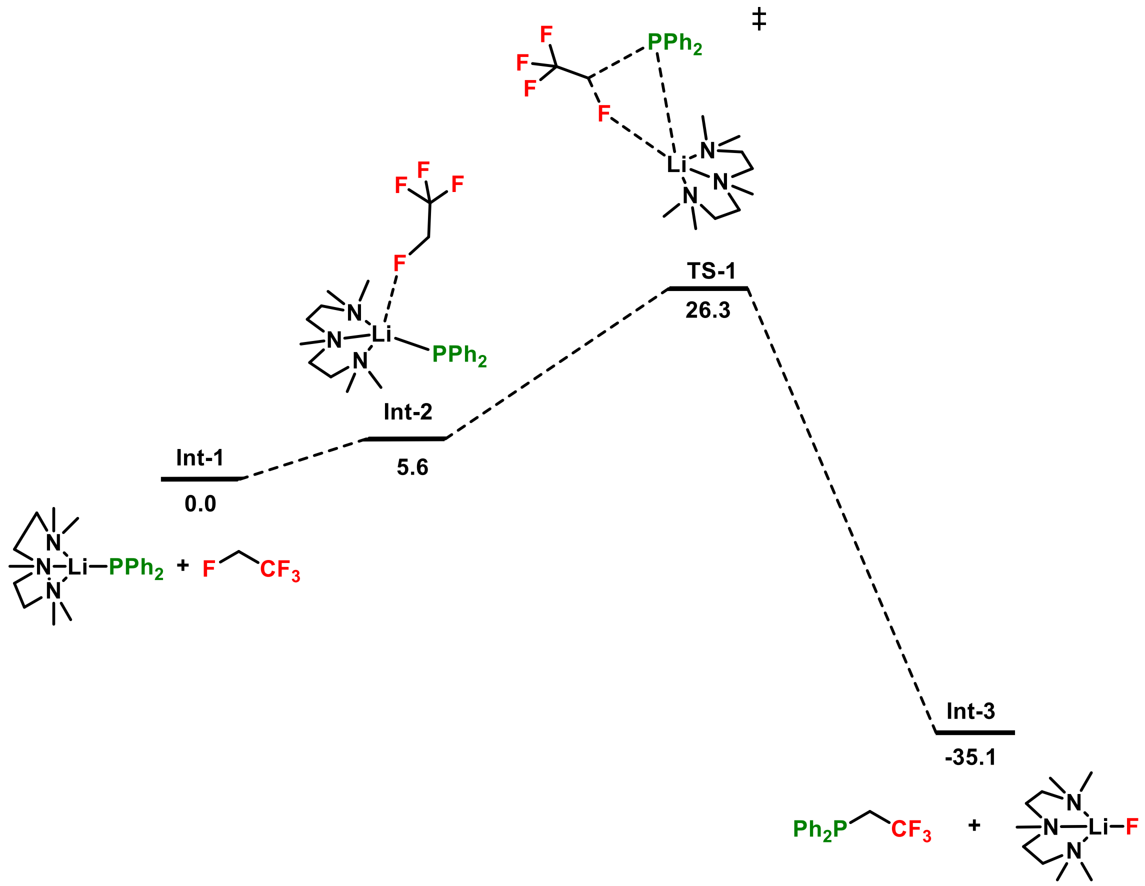

Figure S5: Calculated potential energy surface for the defluorination of HFC-134a with **1·PMDETA** by a s-bond metathesis mechanism. Gibbs Energy in kcal/mol. B3PW91-D3/def2-TZVPP/PCM(benzene)/GoodVibes//B3PW91-D3/def2-SVP(C,H)/def2-TZVP(Li,N,F,P)/PCM(benzene).

7.3 DFT Study: Alternative Mechanisms

An alternative mechanism for the formation of **2** was investigated. It was found that the mechanism proceeds *via* a pathway in which the phosphorus temporarily adopts a hypervalent geometry. Calculations suggests that **1·PMDETA** approaches the α-fluorine of HFC-134a, which weakly coordinates to the phosphorus *via* **TS-2** ($\Delta G_2{}^{\ddagger}{}_{298K}$ = 53.7 kcal/mol), forming a stabilised hypervalent phosphorus centre, **Int-4**, ($\Delta G^{o}{}_{298K}$ = 6.8 kcal/mol). The C–F bond elongates, the Li–F interaction strengthens, and the phosphorus approaches the α-carbon *via* an a-elimination mechanism. This proceeds *via* **TS-3** ($\Delta G_3{}^{\ddagger}{}_{298K}$ = 12.4 kcal/mol), which then forms the experimentally observed product. This mechanism was omitted as **TS-2** was extremely high in energy, and unlikely to occur under the experimental conditions.

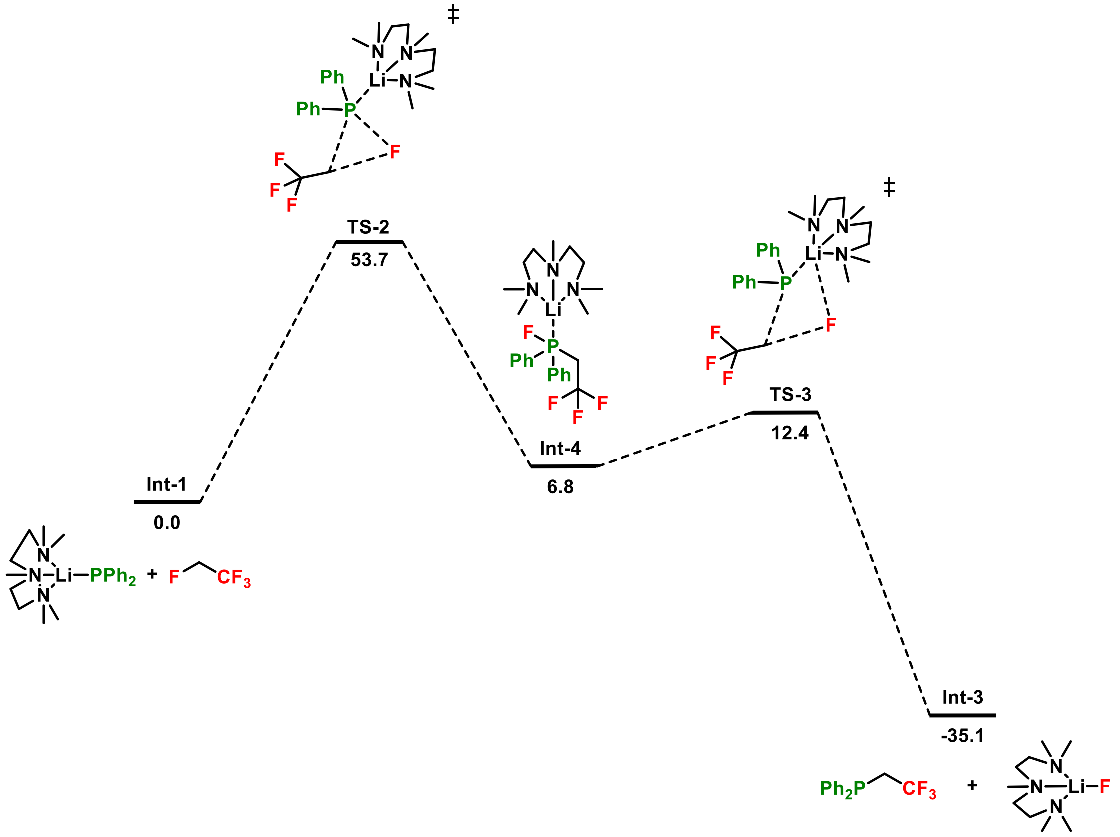

Figure S6: Calculated potential energy surface for the defluorination of HFC-134a *via* **1·PMDETA** in kcal/mol. B3PW91-D3/def2-TZVPP/PCM(benzene)/GoodVibes//B3PW91-D3/def2-SVP(C,H)/def2-TZVP(Li,N,F,P)/PCM(benzene).

Alternative mechanistic pathways were investigated for the formation of **2** *via* the defluorination of HFC-134a. It was found that the reaction could proceed *via* an S_N2 mechanistic pathway. Results suggest that **1·PMDETA** attacks the α-carbon of HFC-134a, displacing free fluoride which then recombines with the lithium cation as the reaction coordinate progresses. This proceeds *via* **TS-4** ($\Delta G_4{}^{\ddagger}{}_{298K}$ = 47.4 kcal/mol), a barrier which is exceptionally high for a reaction that occurs at 80 °C. This elevated energy is likely attributable to the formation of a fluoride anion, which increases the energy of the transition state.

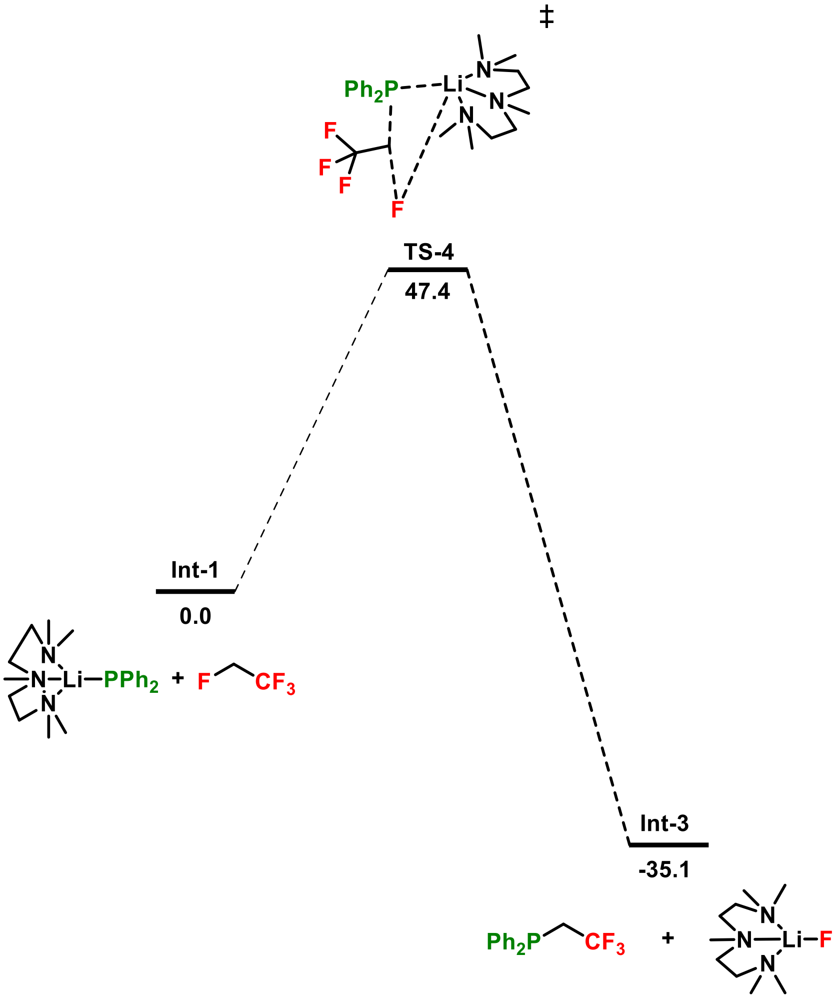

Figure S7: Calculated potential energy surface for the defluorination of HFC-134a *via* **1·PMDETA** in kcal/mol. B3PW91-D3/def2-TZVPP/PCM(benzene)/GoodVibes//B3PW91-D3/def2-SVP(C,H)/def2-TZVP(Li,N,F,P)/PCM(benzene).

7.4 DFT Study: Alternative Products

The reaction of **1·PMDETA** and HFC-134a can also potentially proceed through a deprotonation pathway forming trifluoroetheylene, diphenylphosphine, and **LiF·PMDETA**. In this mechanism, the α-fluorine and hydrogen form an interaction with **1·PMDETA** *via* **TS-5** ($\Delta G_5^{\ddagger}{}_{298K}$ = 30.6 kcal/mol), which then forms a weakly stabilised adduct *via* **Int-6** (ΔG^{o}_{298K} = 22.9 kcal/mol), releasing diphenylphosphine. The intermediate then undergoes 1,2-fluoride migration *via* **TS-6** ($\Delta G_6^{\ddagger}{}_{298K}$ = 34.0 kcal/mol), forming trifluorethylene and **LiF·PMDETA**. This overall reaction is endergonic (ΔG^{o}_{298K} = 1.8 kcal/mol), and the high TS energies suggest this pathway is unlikely to happen experimentally.

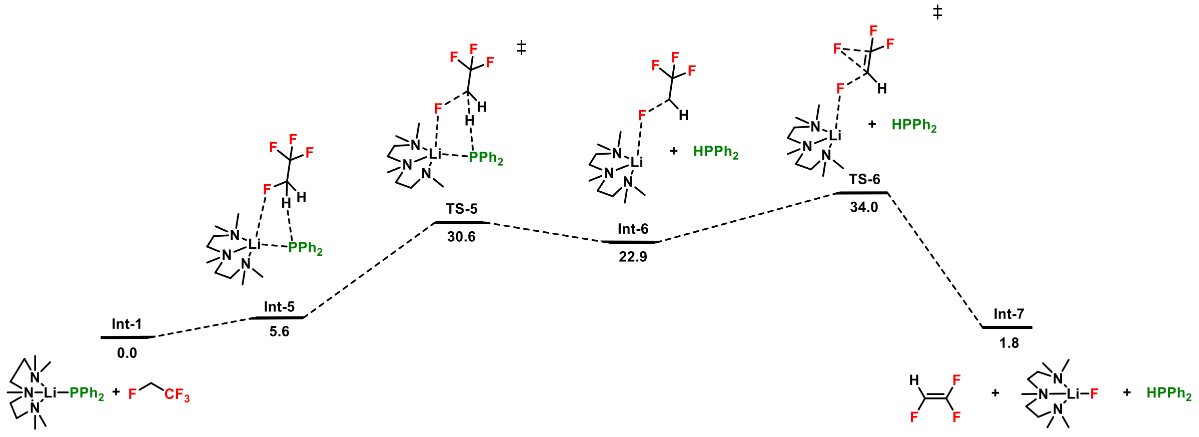

Figure S8: Calculated potential energy surface for the deprotonation of HFC-134a with **1·PMDETA** in kcal/mol. B3PW91-D3/def2-TZVPP/PCM(benzene)/GoodVibes//B3PW91-D3/def2-SVP(C,H)/def2- TZVP(Li,N,F,P)/PCM(benzene).

It was also of interest to investigate why C–F bond occurred selectively on the fluorine on the β-carbon and not the α-carbon. It was found that the reaction mechanism proceeds *via* a σ-bond metathesis mechanism where the PPh_2 moiety in **1·PMDETA** approaches the α-carbon of HFC-134a *via* **TS-7** ($\Delta G_1^{\ddagger}{}_{298K}$ = 61.7 kcal/mol) undergoing σ-bond metathesis, forming **Int-4** (ΔG^{o}_{298K} = - 16.8 kcal/mol). This reaction is unlikely to occur experimentally due to the high energy barriers associated with it. Additionally, the product formed is less thermodynamically stable in comparison to the experimentally observed product.

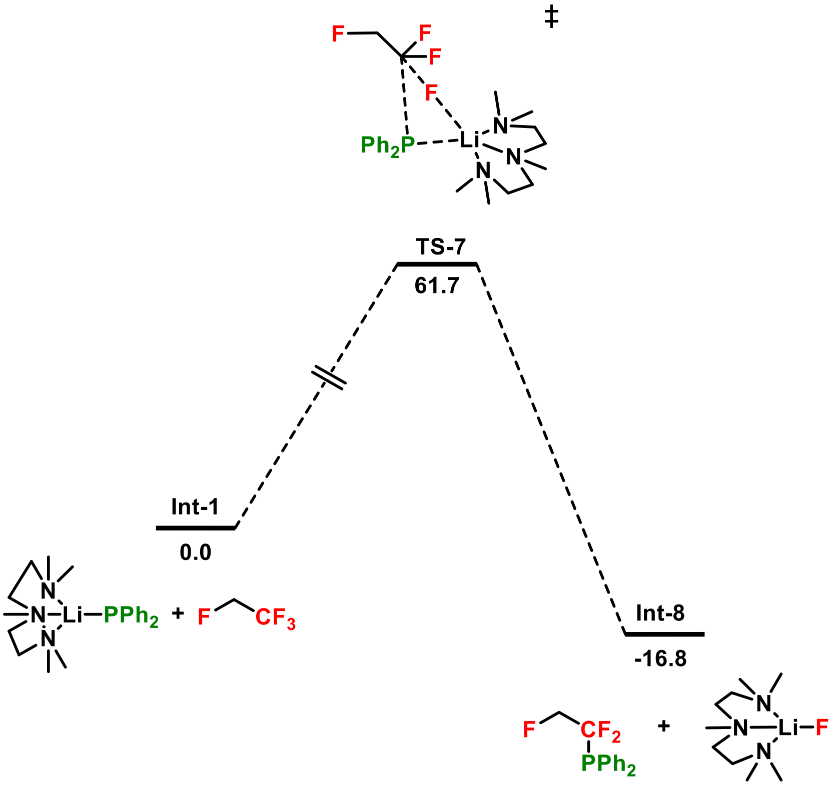

Figure S9: Calculated potential energy surface for the defluorination of the CF_3 group of HFC-134a *via* **1·PMDETA** in kcal/mol. B3PW91-D3/def2-TZVPP/PCM(benzene)/GoodVibes//B3PW91-D3/def2-SVP(C,H)/def2-TZVP(Li,N,F,P)/PCM(benzene).

7.5 Functional Testing

Functional benchmarking calculations were performed using the hybrid GGA functional B3PW91, the Minnesota hybrid meta functionals M062X, M06L, and the long-range corrected functional ωB97xD with Grimme's D2 dispersion correction. The basis set along with solvent corrections (Benzene, PCM) and dispersion corrections (GD3) was kept constant throughout except from ωB97xD which includes Grimme's D2 dispersion corrections in the functional.

Functional benchmarking calculations suggested that the B3PW91 was the most appropriate functional for modelling this system. Previous work reported by our group suggests that the B3PW91 performs well for closely related reactions.[26]

**Functional**	**$\Delta G_1^{\ddagger}$ (kcal/mol)<br>TS-1**
B3PW91	26.4
ωB97xD	29.8
M062X	27.6
M06L	24.1

Table S6: Calculated transition state energies with various density functionals.

7.6 NBO Analysis

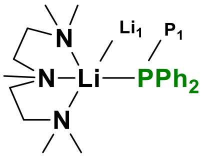

Int-1

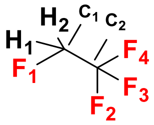

HFC-134a

	Int-1	HFC-134a	Int-2	TS-1	$Ph_2PCH_2CF_3$	LiF·PMDETA
P1	0.032		-0.326	-0.220	0.075	
Li1	0.835		0.220	0.561		0.519
C1		0.057	0.048	-0.149	-0.224	
C2		0.449	0.459	0.541	0.465	
F1		-0.215	-0.232	-0.419		-0.722
F2		-0.163	-0.162	-0.165	-0.168	
F3		-0.163	-0.169	-0.167	-0.172	
F4		-0.168	-0.178	-0.191	-0.177	
H1		0.101	0.122	0.186	0.124	
H2		0.101	0.105	0.096	0.118	

Table S7: NPA charges of key stationary points for main reaction mechanism.

	**Int-1**	**HFC-134a**	**Int-2**	**TS-1**	**$Ph_2PCH_2CF_3$**	**LiF·PMDETA**
Li1-P1	0.1001		0.1039	0.0044		
C1-F1		0.9033	0.8919	0.5239		
C1-C2		0.0000	0.9581	0.9698	0.9914	
C1-P1			0.0132	0.3302	0.9015	
Li1-F1			0.0005	0.0335		0.0714
C2-F2		0.9092	0.8998	0.9078	0.9085	
C2-F3		0.9191	0.9120	0.8762	0.8987	
C2-F4		0.9191	0.9120	0.9155	0.9072	

Table S8: Wiberg Bond Indices of key stationary points for main reaction mechanism.

8.1 Computational Coordinates

HFC-134a

SCF (RB3PW91) = -476.741718133

E(SCF)+ZPE(0 K)= -476.696523

H(298 K)= -476.690104

G(298 K)= -476.726031

Lowest frequency = 111.1911 cm-1

C -2.32339300 1.90394100 0.01687300

C -1.79648100 2.58739700 1.26991200

H -0.69609000 2.56722900 1.24318200

H -2.15516900 3.62819700 1.27805400

F -2.24988900 1.92724000 2.38549600

F -1.87218400 2.56140300 -1.06763800

F -3.66473300 1.90276400 -0.02685900

F -1.91198800 0.62944900 -0.06939900

PMDETA.LiPPh2

SCF (RB3PW91) = -1332.44305604

E(SCF)+ZPE(0 K)= -1331.930684

H(298 K)= -1331.902058

G(298 K)= -1331.987098

Lowest frequency = 29.0148 cm-1

P 3.60185300 5.39668100 2.61434900

Li 2.88836800 7.40167600 3.96039400

N 2.71298600 9.13905500 2.68320400

N 4.17388700 8.64066800 5.15634800

N 1.63231900 7.23788700 5.78734700

C 1.37312700 9.38989200 2.15664700

C 3.64523600 8.92577800 1.57516600

C 3.16019300 10.23018700 3.55137300

C 4.39385500 9.85232900 4.36077100

C 5.43139900 7.96807300 5.46962700

C 3.39198900 8.90615800 6.36916400

C 2.58680400 7.69331100 6.80551300

C 1.11678100 5.92195500 6.16901000

C 0.51591200 8.16551800 5.62353700

C 2.00615600 5.65269700 1.76516400

C 2.04558800 5.97644000 0.38726700

C 0.90341800 6.36053900 -0.31570200

C -0.34076700 6.41152200 0.32002600

C -0.41439000 6.06898100 1.67486800

C 0.73117700 5.70439400 2.37955100

C 3.24200800 4.27200800 3.99404800

C 2.18571400 3.33426100 4.05820500

C 2.02138300 2.49463300 5.15910500

C 2.91611300 2.53221100 6.23373300

C 3.99583800 3.42116000 6.17367100

C 4.15108700 4.27134000 5.08106900

H 1.04349000 8.52574000 1.56326800

H 1.33787700 10.29437100 1.51566800

H 0.66058800 9.52755900 2.98194800

H 3.77734300 9.84634500 0.97039300

H 3.25808600 8.13172200 0.92567100

H 4.62612300 8.59714100 1.94160600

H 3.37452500 11.15273400 2.97164500

H 2.33158300 10.48485100 4.23085600

H 4.70020600 10.71049300 4.99237600

H 5.23542800 9.66119400 3.68021300

H 6.12747300 8.62158400 6.03313300

H 5.92052500 7.63732000 4.54250000

H 5.23926200 7.07225600 6.07505100

H 2.71304500 9.74862600 6.17717100

H 4.04524400 9.23097000 7.20354600

H 3.26820600 6.85303800 7.00901800

H 2.07351200 7.92140700 7.76348500

H 0.58492700 5.95988900 7.14134500

H 1.93700500 5.19774400 6.23928300

H 0.41238500 5.55725200 5.41075400

H 0.86908400 9.16582300 5.34139900

H -0.08446300 8.26579800 6.55105700

H -0.14275600 7.80630700 4.81973800

H 3.00322300 5.91547000 -0.13960300

H 0.98396600 6.60745000 -1.37861400

H -1.23828700 6.70164100 -0.23188100

H -1.37756500 6.09630600 2.19389500

H 0.64108700 5.46080100 3.43936200

H 1.48301800 3.25857400 3.22505300

H 1.18397200 1.79001400 5.17357100

H 2.78267300 1.87286800 7.09498200

H 4.72067700 3.45795800 6.99285000

H 4.99971000 4.96250300 5.05710000

Int-2

SCF (RB3PW91) = -1809.19381329

E(SCF)+ZPE(0 K)= -1808.635706

H(298 K)= -1808.599352

G(298 K)= -1808.705758

Lowest frequency = 20.3698 cm-1

P 0.63023600 -0.72987700 1.28005600

Li -0.70374800 1.08265900 0.20521100

N -1.92229800 2.21954500 1.55657600

N -2.49882700 0.63840200 -0.83056700

N 0.00599100 2.05603800 -1.58449100

C -1.40174900 3.50738300 2.00787200

C -2.12106300 1.33569600 2.70807200

C -3.15964900 2.39704900 0.78930000

C -3.56991600 1.13862700 0.03520700

C -2.66242500 -0.78487700 -1.11912400

C -2.35156100 1.41910400 -2.06529500

C -0.90997600 1.43690000 -2.55237600

C 1.39476200 1.76263700 -1.94163800

C -0.17247400 3.50349500 -1.50843100

C 0.36198700 -2.02313100 0.03154400
C 0.83027400 -2.02973200 -1.30234800
C 0.40570300 -2.98616800 -2.22430200
C -0.50696600 -3.97955700 -1.85568400
C -0.97088800 -4.00590000 -0.53657200
C -0.53612600 -3.05708500 0.38661600
C 2.19533300 0.09709000 0.88691800
C 2.36222400 1.40899400 1.39544200
C 3.53426200 2.13495600 1.19859900
C 4.59237100 1.58526900 0.46660900
C 4.45984100 0.28319800 -0.02709200
C 3.29586600 -0.45313000 0.19114600
H -0.44613000 3.35862600 2.52923000
H -2.09599800 4.02098900 2.70327500
H -1.22550200 4.16955800 1.14900000
H -1.18271500 1.23847500 3.26736500
H -2.40028300 0.32493000 2.38397500
H -2.90379000 1.72399100 3.39084000
H -3.99946200 2.71772400 1.44085400
H -2.99678200 3.22093400 0.07687600
H -4.50257500 1.33941100 -0.53020700
H -3.81712600 0.34419000 0.75367100
H -2.65849800 -1.35925000 -0.18411000
H -1.81898800 -1.15792100 -1.71544100
H -3.60383600 -0.99217000 -1.66659600
H -2.69235600 2.44819800 -1.88405700
H -3.00468200 1.02291600 -2.86762800
H -0.57026100 0.40174500 -2.71083100
H -0.85764300 1.94481000 -3.53812900
H 1.65742200 2.17271900 -2.93766200
H 1.55761500 0.67933000 -1.95228500
H 2.07486100 2.18507200 -1.19059200
H 0.05014300 4.00168700 -2.47418200
H 0.50211200 3.91605300 -0.74480700
H -1.20071100 3.76401200 -1.22524400
H 1.53183800 -1.26181600 -1.63212800
H 0.78971400 -2.95014700 -3.24851200
H -0.83981100 -4.72755100 -2.57968700
H -1.67328100 -4.78289000 -0.21998500
H -0.90671100 -3.10087700 1.41598100
H 1.53919100 1.87044000 1.95164800
H 3.61841600 3.14420100 1.61244300
H 5.50827700 2.15619200 0.29511000
H 5.28523800 -0.17395300 -0.58172500
H 3.24211300 -1.48037200 -0.17661700
C 1.74069300 1.44404000 5.11827200
C 1.26195500 0.02935500 4.85086500
F 0.99380500 2.36006600 4.46681700
F 1.68897400 1.75687200 6.42655900
F 3.01134100 1.58399800 4.71193200
H 1.91470800 -0.66850000 5.39665400
F -0.03555500 -0.09801900 5.30111500

H 1.29739300 -0.18609900 3.76732800

TS-1

SCF (RB3PW91) = -1809.16079913

E(SCF)+ZPE(0 K)= -1808.604246

H(298 K)= -1808.568853

G(298 K)= -1808.671088

Lowest imaginary frequency = -429.7119 cm-1

P -2.11219700 -2.12761900 2.60503800

Li 0.00541600 1.63850800 -0.83563500

N -0.09128600 2.67523400 -2.67473600

N 1.70258500 0.60846800 -1.55925900

N 1.23531900 2.32155100 0.74677900

C 0.45515300 4.02955700 -2.66473000

C -1.48472100 2.69691600 -3.12293600

C 0.71479400 1.76758700 -3.50477700

C 1.99386500 1.33274100 -2.80135100

C 1.42434000 -0.80324200 -1.80408900

C 2.72537100 0.79468800 -0.52628000

C 2.57505400 2.13982300 0.17190300

C 1.04081500 1.45991800 1.91696800

C 1.01344600 3.71847800 1.11512600

C -2.49553400 -3.41362200 1.37477400

C -1.77280800 -4.61463600 1.19841900

C -2.18317500 -5.58224300 0.28343100

C -3.34037300 -5.39650100 -0.48136200

C -4.08377100 -4.22699500 -0.30892700

C -3.66629800 -3.25285700 0.59939100

C -0.29938900 -2.08804700 2.66806500

C 0.57803300 -2.46032400 1.61994400

C 1.95943300 -2.27866900 1.72144800

C 2.52206100 -1.69556300 2.85952700

C 1.67242900 -1.29541000 3.89899100

C 0.29708200 -1.48931000 3.80513400

H -0.13168400 4.66337400 -1.98444700

H 0.43803900 4.49368900 -3.67145900

H 1.49501200 4.03054300 -2.31042000

H -1.58187100 3.05757800 -4.16621200

H -2.07279000 3.36053000 -2.47284000

H -1.91525600 1.68883400 -3.05054100

H 0.10237300 0.87942800 -3.71962400

H 0.95923400 2.22555600 -4.48474400

H 2.60186800 2.21775100 -2.56517800

H 2.60647700 0.71990500 -3.49169900

H 2.31564100 -1.34866800 -2.17358000

H 0.61973000 -0.91000900 -2.54382000

H 1.07705700 -1.27728000 -0.87688800

H 3.75220100 0.70227200 -0.93309300

H 2.61400500 -0.01447100 0.20955400

H 3.36287400 2.24084100 0.94633900

H 2.74590800 2.95618300 -0.54770200

H 1.14347300 0.40001700 1.65217300

H 0.03507200 1.60745800 2.32839200

H 1.77687600 1.67656100 2.71514600

H 1.72075300 4.06405300 1.89527000

H -0.00864200 3.84190400 1.50149900

H 1.13037700 4.36732000 0.23523500

H -0.88125500 -4.79796300 1.80384300

H -1.59916900 -6.50121500 0.17422200

H -3.66120600 -6.15868900 -1.19629500

H -4.99716800 -4.06728200 -0.88963800

H -4.26897800 -2.34660200 0.72452600

H 0.16859300 -2.91227700 0.71291900

H 2.60355000 -2.59760100 0.89600300

H 3.60327000 -1.55718700 2.93867100

H 2.09072300 -0.83428900 4.79855100

H -0.34674100 -1.17958900 4.63436800

C -2.65887000 0.78291100 1.27424100

C -1.82891400 -0.28374500 0.56978200

F -2.27804200 1.00405600 2.54678400

F -2.55037300 2.00159700 0.67202700

F -3.96793000 0.48280800 1.27075800

H -2.37656200 -1.10907200 0.12307100

F -1.38115900 0.44990600 -0.82536700

H -0.84209500 -0.47754900 0.97927600

PMDETA.LiF

SCF (RB3PW91) = -628.174205372

E(SCF)+ZPE(0 K)= -627.843813

H(298 K)= -627.825499

G(298 K)= -627.886809

Lowest frequency = 59.4520 cm-1

C 3.66877000 3.39260300 0.91558500

H 3.25082600 3.88289900 0.01134800

H 2.82628300 3.06865600 1.54281300

H 4.21243100 2.49126200 0.59738000

C 5.70363700 4.66482500 0.83895900

H 5.40464500 5.28850600 -0.02881200

H 6.21216900 3.76665400 0.46011400

H 6.42778500 5.21268900 1.45746800

C 3.86045000 5.43787400 2.19917400

H 3.19914500 5.90642400 1.44008700

H 4.61749400 6.19239700 2.45935900

C 3.05296400 5.09379300 3.44473900

H 2.45884700 5.97627000 3.75860700

H 2.32127400 4.31125800 3.19824200

C 4.51232700 5.71193200 5.27626800

H 3.76038600 6.29747300 5.84505900

H 5.03647800 6.39409900 4.59338200

H 5.26498100 5.32111700 5.97466200

C 3.26132600 3.64055300 5.39958700

H 2.24184900 3.95911000 5.70140000

H 3.84409500 3.57883700 6.32996800

C 3.18225000 2.26025600 4.75647400

H 2.71102100 1.55466800 5.47426600

H 2.51298300 2.29811400 3.88236100

C 4.33237900 0.62071600 3.41902400

H 3.88949700 -0.25392800 3.93964200

H 5.31506200 0.32319200 3.02465600

H 3.68569400 0.87431100 2.56668100

C 5.38963700 1.46850800 5.39732100

H 4.98450800 0.67811500 6.06300000

H 5.59209700 2.36641400 5.99557400

H 6.35533500 1.13556500 4.99408100

Li 5.46996500 3.51507400 3.47222800

N 4.56127900 4.27028100 1.65881800

N 3.91310400 4.61535700 4.52832100

N 4.48244200 1.77548100 4.29413000

F 7.09794700 3.86066100 3.71494400

$PPh_2CH_2CF_3$

SCF (RB3PW91) = -1181.06302481

E(SCF)+ZPE(0 K)= -1180.836780

H(298 K)= -1180.819831

G(298 K)= -1180.884065

Lowest frequency = 11.1176 cm-1

C -2.02219900 3.29266600 5.94314400

C -1.58799100 2.22091000 4.97469300

H -1.22189900 2.72002700 4.06569600

H -0.74821900 1.69480700 5.45110800

F -3.04176100 4.03829500 5.47320200

F -1.00400200 4.14631800 6.20216900

F -2.41430100 2.78649600 7.13032700

P -2.90558200 0.96359000 4.58897500

C -3.91099400 1.89388000 3.36790900

C -3.44815300 2.21641900 2.08259800

C -5.19455000 2.30389800 3.75325900

C -4.25129900 2.94384000 1.20569300

H -2.45534400 1.88732400 1.76279000

C -5.99811000 3.03562200 2.87598500

H -5.56510200 2.05190600 4.75085300

C -5.52746900 3.35607700 1.60190100

H -3.88218500 3.18902200 0.20632500

H -6.99619400 3.35299900 3.18864900

H -6.15672500 3.92508300 0.91257800

C -1.98090600 -0.15938900 3.47663100

C -0.61772900 -0.07397200 3.15840100

C -2.73534100 -1.20751400 2.91905500

C -0.02736500 -1.01075300 2.30484100

H 0.00740000 0.72375600 3.56329200

C -2.14939600 -2.13252600 2.05856200

H -3.79961000 -1.29296400 3.15940100

C -0.78894400 -2.03867800 1.75021800

H 1.03704300 -0.92894700 2.06956700

H -2.75553300 -2.93526500 1.63087600

H -0.32502800 -2.76733500 1.08073600

TS-2

SCF (RB3PW91) = -1809.11851434

E(SCF)+ZPE(0 K)= -1808.562227

H(298 K)= -1808.526687

G(298 K)= -1808.628959

Lowest imaginary frequency = -551.2704 cm-1

P 1.27196100 -0.60113400 0.66857400

Li -0.80752000 0.65334200 0.17831400

N -0.31229600 2.68046800 -0.22022600

N -2.32125700 1.40889900 1.44752300

N -2.36226400 -0.43680200 -0.85652100

C 0.04780000 2.99682400 -1.60082000

C 0.81057400 2.99036500 0.67047700

C -1.52941900 3.38728500 0.18679200

C -2.09124400 2.85721600 1.50056400

C -2.27676700 0.79975300 2.77395400

C -3.55541300 1.06650700 0.73137000

C -3.47946700 -0.31002600 0.08860700

C -2.12752400 -1.84970500 -1.15873000

C -2.60623600 0.29841900 -2.09622600

C 2.01002400 -0.12545800 -0.93532400

C 1.38958600 -0.44298100 -2.16238100

C 1.86443600 0.06289900 -3.37151300

C 2.97305900 0.91490700 -3.39101800

C 3.60273400 1.23727700 -2.18709300

C 3.13497600 0.72081700 -0.97644600

C 0.83478000 -2.34616600 0.49962900

C 0.02396700 -2.86352200 1.53513900

C -0.35590300 -4.20548200 1.56385300

C 0.06224100 -5.07413300 0.55275000

C 0.88870100 -4.58574600 -0.46582600

C 1.28279500 -3.24954200 -0.48556900

H 0.93132000 2.41300700 -1.89413500

H 0.27607800 4.07325200 -1.73693800

H -0.77692200 2.73298000 -2.27868500

H 0.62593300 2.62421200 1.68774400

H 1.00799300 4.08058200 0.71030500

H 1.71484000 2.47913200 0.32251900

H -1.35486800 4.47949400 0.28309400

H -2.27530000 3.26843500 -0.61556200

H -3.01426400 3.41245100 1.76100000

H -1.37712900 3.05672600 2.31144500

H -2.41561300 -0.28769500 2.69735000

H -3.05986900 1.20050500 3.44824100

H -1.29208400 0.97219100 3.23093300

H -3.73427400 1.82383300 -0.04470500

H -4.43554300 1.10893800 1.40367400

H -3.33621900 -1.07166900 0.86976200

H -4.44908300 -0.54188900 -0.39899000

H -1.87745300 -2.39942100 -0.24245700

H -1.27490500 -1.95397500 -1.84166900

H -3.01171300 -2.32009600 -1.63359700

H -2.78658500 1.36259000 -1.89422600

H -3.48149800 -0.09606600 -2.65116400

H -1.72183600 0.22885500 -2.74568200

H 0.51221100 -1.09278500 -2.16869900

H 1.35928500 -0.20305200 -4.30461700

H 3.34413500 1.31833400 -4.33675400

H 4.47648700 1.89501500 -2.18663800

H 3.63013400 0.97653400 -0.03738100

H -0.32995900 -2.18607500 2.32071100

H -0.98847100 -4.57366900 2.37677500

H -0.24092700 -6.12413400 0.56362900

H 1.24149800 -5.26164000 -1.25035300

H 1.95147600 -2.89916900 -1.27449800

C 4.23020700 -1.42183300 1.77096500

C 3.00605600 -0.85499600 2.43906400

F 3.99168000 -2.12774200 0.65043600

F 5.16135800 -0.50221300 1.46465000

F 4.82935300 -2.29310000 2.63834600

H 2.31193700 -1.68807600 2.62005300

F 2.93668800 0.88527700 2.05610900

H 3.32352800 -0.47733100 3.42009400

Int-4

SCF (RB3PW91) = -1809.19447235

E(SCF)+ZPE(0 K)= -1808.637507

H(298 K)= -1808.601746

G(298 K)= -1808.704561

Lowest frequency = 21.0919 cm-1

P 0.90197800 -0.19168300 0.03563200

Li -1.14116900 1.26270500 -0.22175700

N -0.78806600 3.39686800 0.05145600

N -2.67722300 1.49873700 1.25040700

N -2.65596900 0.63651600 -1.57707600

C -0.57551800 4.24821300 -1.11789800

C 0.40309600 3.42719700 0.90422400

C -1.98312500 3.83767300 0.78126000

C -2.45615400 2.83128500 1.82047700

C -2.69651000 0.47993500 2.29668700

C -3.89163000 1.43103100 0.42542800

C -3.77464600 0.37611300 -0.66398200

C -2.32760900 -0.56818000 -2.34071500

C -2.94175300 1.73335600 -2.49916400

C 1.02944300 -0.21143900 -1.92614100

C 0.97137400 -1.31837800 -2.78953100

C 0.81196900 -1.16458400 -4.17230500

C 0.70639800 0.10744400 -4.73778700

C 0.77857000 1.22845200 -3.90327100

C 0.93807600 1.05734900 -2.52799100

C 0.79921600 -2.00932200 0.16873900

C -0.42383800 -2.62278400 0.46808800

C -0.51724400 -4.01149100 0.58493700

C 0.61720500 -4.80546100 0.40516300

C 1.84284500 -4.20189100 0.11071600

C 1.93267800 -2.81494700 -0.00704600

H 0.31483800 3.91590900 -1.66844900

H -0.42778700 5.31036800 -0.83780300

H -1.43689300 4.18663700 -1.79691500

H 0.26895100 2.78030500 1.77963600

H 0.62990100 4.45257000 1.25885700

H 1.27120400 3.04567000 0.34918900

H -1.81391900 4.81409100 1.28085700

H -2.77902500 4.01224300 0.04084900

H -3.36709600 3.22498100 2.31553300

H -1.69949600 2.73270800 2.61233700

H -2.81406000 -0.51960600 1.85709800

H -3.52198000 0.63841000 3.01890700

H -1.74543100 0.48822300 2.84520400

H -4.06572700 2.41248400 -0.03617400

H -4.78566100 1.22641400 1.04669900

H -3.60415900 -0.60857200 -0.20295600

H -4.73636300 0.30057600 -1.21230200

H -1.96194100 -1.35730100 -1.66945200

H -1.52652400 -0.34501000 -3.05468800

H -3.20497700 -0.94974800 -2.90069100

H -3.19855700 2.65057000 -1.95248200

H -3.78491900 1.49637200 -3.17920800

H -2.04876600 1.93668400 -3.10754300

H 1.03824300 -2.33059900 -2.38310000

H 0.76490400 -2.05038200 -4.81338400

H 0.57494300 0.22645200 -5.81668900

H 0.71213300 2.23511000 -4.32811300

H 0.99107800 1.94661400 -1.88901400

H -1.31486800 -2.00581200 0.61816400

H -1.47967300 -4.47553800 0.81848200

H 0.54747300 -5.89253800 0.49734100

H 2.73630300 -4.81732500 -0.02575000

H 2.89301900 -2.34570100 -0.23523400

C 2.17999300 -0.86330800 2.84380700

C 1.18850000 -0.06648200 2.06574900

F 1.81062100 -2.15805600 3.00264100

F 3.42193600 -0.87799700 2.30470000

F 2.36026200 -0.38882300 4.12731000

H 0.20096500 -0.28862400 2.49598800

F 2.45764600 0.37666800 -0.02034400

H 1.42065700 0.99277700 2.24399100

TS-3

SCF (RB3PW91) = -1809.18548142

E(SCF)+ZPE(0 K)= -1808.629373

H(298 K)= -1808.595061

G(298 K)= -1808.695041

Lowest imaginary frequency = -104.0130 cm-1

P 0.82684200 -1.22431000 0.25031100

Li -0.64399600 0.94448400 0.13526100

N 0.20947900 2.86267900 0.76286500

N -2.28908300 1.48644200 1.41974300

N -2.09752000 0.82395700 -1.47142000

C 0.83255800 3.55240600 -0.36518700

C 1.18972100 2.71986800 1.84471000

C -0.98293700 3.57930000 1.22858100

C -1.86460800 2.70783800 2.11314900

C -2.67282900 0.43732900 2.36283500

C -3.36802400 1.74946900 0.45648600

C -3.34654800 0.76625400 -0.70032900

C -1.98356600 -0.37423200 -2.30747400

C -2.03453500 2.01222100 -2.32050600

C 1.19997800 -1.16771200 -1.64894700

C 0.79486400 -2.28656500 -2.40102800

C 0.75263600 -2.26483800 -3.79421800

C 1.10663000 -1.10339200 -4.49126100

C 1.51801200 0.01866900 -3.77068700

C 1.56982900 -0.01920200 -2.37168600

C 1.70074700 -2.82009600 0.46231700

C 1.04533100 -3.92405600 1.02826900

C 1.71177100 -5.13786500 1.19445500

C 3.05093300 -5.26149500 0.81320300

C 3.71315600 -4.16638100 0.25452900

C 3.03949400 -2.95788500 0.07160900

H 1.70566000 2.97890200 -0.70609400

H 1.17230700 4.57330300 -0.09814800

H 0.12597800 3.63396400 -1.20303100

H 0.76149700 2.16988000 2.69201900

H 1.53135400 3.70824700 2.21156600

H 2.05195200 2.14356900 1.49295600

H -0.71361100 4.50494600 1.77752800

H -1.55104000 3.90675400 0.34406700

H -2.73017700 3.29911800 2.47318900

H -1.30529200 2.40942200 3.01118000

H -2.92975600 -0.48368500 1.82172600

H -3.54133600 0.73237900 2.98457700

H -1.83056500 0.19991400 3.02615900

H -3.25933700 2.77189100 0.06909700

H -4.35850600 1.71812300 0.95165500

H -3.44584400 -0.25775100 -0.31018100

H -4.22812500 0.94007000 -1.35141300

H -1.88411800 -1.26441700 -1.67206600

H -1.08543600 -0.30941800 -2.93187500

H -2.86550600 -0.49298400 -2.96832700

H -2.12260200 2.93100000 -1.72474900

H -2.84135800 2.02357300 -3.08100300

H -1.06717500 2.03515500 -2.84264100

H 0.49553700 -3.20153400 -1.87753400

H 0.43955600 -3.15742000 -4.34432400

H 1.06915300 -1.07932600 -5.58370000

H 1.81181700 0.93034300 -4.30060800

H 1.91499800 0.86637400 -1.83424200

H -0.00087600 -3.83520000 1.33557600

H 1.18372300 -5.99354600 1.62461400

H 3.57553800 -6.21094100 0.94963400

H 4.76083900 -4.25505900 -0.04622400

H 3.55524900 -2.10784500 -0.38278800

C 2.05659800 -1.02810600 3.20040600

C 0.83348500 -1.00978400 2.35488400

F 2.67397900 -2.23075100 3.23428900

F 2.99327100 -0.12164600 2.82200900

F 1.80132900 -0.72407600 4.52652000

H 0.16295100 -1.80210100 2.71824600

F 1.76041800 0.17486000 0.35009700

H 0.34514000 -0.03908500 2.53137900

TS-4

SCF (RB3PW91) =	-1809.12634254
E(SCF)+ZPE(0 K)=	-1808.570663
H(298 K)=	-1808.534997
G(298 K)=	-1808.638589

Lowest imaginary frequency = -489.9897 cm-1

P 2.55702900 5.41496100 3.80802000

Li 1.88138800 7.74687500 4.19042600

N 3.20942600 9.16109200 5.05279400

N 0.65299100 8.04356300 5.86357800

N 0.33614200 8.42201700 2.93579000

C 3.81600300 10.11136300 4.12500600

C 4.25318600 8.37262300 5.70801000

C 2.34673700 9.84543500 6.02387000

C 1.43173300 8.88371700 6.77495300

C 0.22191000 6.79558000 6.49119600

C -0.48281200 8.75238700 5.25522200

C -0.79120600 8.22431100 3.86122700

C 0.19214500 7.56161900 1.75828400

C 0.46568500 9.81437400 2.51913700

C 2.77289300 5.00143600 2.04332700

C 1.73723000 4.40901500 1.29995400

C 1.88644700 4.16766000 -0.06814500

C 3.07447800 4.50095300 -0.72051100

C 4.11647000 5.08554600 0.00626300

C 3.96313100 5.33911200 1.36890500

C 3.87799500 4.51383000 4.63376800

C 4.67176000 3.50245300 4.05142700

C 5.57032000 2.76203500 4.81893700

C 5.69418000 2.98906500 6.19278300

C 4.89384000 3.96394900 6.79510200

C 3.99982300 4.70866500 6.02848900

H 4.40218100 9.56848400 3.36911400

H 4.49081300 10.82692000 4.63679000

H 3.03710200 10.68661500 3.60607800

H 3.81423700 7.63414000 6.38903500

H 4.95013800 9.01037800 6.28836600

H 4.82480200 7.81234000 4.95521200

H 2.94099800 10.43115300 6.75567200

H 1.73905000 10.57703300 5.46907800

H 0.78818400 9.46112000 7.46921100

H 2.03660500 8.21920900 7.40902400

H -0.27766000 6.15673100 5.75143500

H -0.46044000 6.97301000 7.34627500

H 1.09895600 6.24133800 6.85434600

H -0.24870400 9.82567500 5.20485100

H -1.38696700 8.66960600 5.88949900

H -0.97906900 7.14192800 3.91568500

H -1.71529300 8.70288400 3.47674200

H 0.08238500 6.51883400 2.08052000

H 1.08909700 7.63767200 1.12664300

H -0.68990200 7.84411600 1.14903800

H 0.59792000 10.47777800 3.38494100

H -0.42445200 10.16413500 1.95742400

H 1.34572100 9.92829800 1.86953100

H 0.80125800 4.15428700 1.80184100

H 1.06551600 3.70906800 -0.62650400

H 3.19033700 4.30711400 -1.79022300

H 5.05194800 5.35210300 -0.49390000

H 4.78113400 5.80562200 1.92593400

H 4.58581800 3.29644800 2.98210600

H 6.17760500 1.98998200 4.33757700

H 6.39898600 2.40486600 6.78972600

H 4.96551500 4.14423600 7.87149300

H 3.37457000 5.46155700 6.51868500

C -0.36120700 3.00405800 4.83270300

C 0.92972700 3.75548500 4.55634100

H 1.38721500 3.93975400 5.53496800

H 1.55649900 3.07889600 3.96306300

F -0.08716800 5.00989500 3.74370300

F -0.02746500 1.86277600 5.51173200

F -1.02658200 2.59188200 3.73849700

F -1.23491900 3.65147700 5.63108300

Int-5

SCF (RB3PW91) = -1809.19416570

E(SCF)+ZPE(0 K)= -1808.635677

H(298 K)= -1808.599388

G(298 K)= -1808.704585

Lowest frequency = 20.2797 cm-1

P 1.91980800 0.37685300 -0.54353600

Li -0.12057200 1.74856800 -0.13378000

N 0.49065700 3.82886100 -0.20982600

N -1.25985900 2.36192200 1.59745800

N -1.99671700 1.15649700 -1.01417900

C 0.55815500 4.39596700 -1.55316000

C 1.81150200 3.88286400 0.41916000

C -0.52238400 4.50831000 0.60180900

C -0.85284200 3.74259700 1.87631100

C -1.06104900 1.49943900 2.75981300

C -2.64287900 2.27959700 1.10634200

C -2.84900900 1.09223900 0.17995300

C -1.96099700 -0.14822300 -1.67681600

C -2.45590100 2.16704500 -1.96354700

C 1.39782100 -0.44645000 -2.07141100

C 0.98475300 -1.78620500 -2.25121600

C 0.45947600 -2.23346400 -3.46343100

C 0.32275900 -1.36783900 -4.55222900

C 0.73866900 -0.03903200 -4.40522100

C 1.27249400 0.40494300 -3.19822900

C 2.34216700 -0.88462800 0.68079900

C 2.84428500 -2.17959500 0.41701000

C 3.13494800 -3.07263400 1.44938600

C 2.97381200 -2.70242800 2.78786100

C 2.53217900 -1.40416000 3.07486300

C 2.22044300 -0.52217300 2.04455500

H 1.28136100 3.82816800 -2.15596800

H 0.87167200 5.45970300 -1.54767500

H -0.42293800 4.33024300 -2.04420100

H 1.80649400 3.36235300 1.38645300

H 2.14504300 4.92796000 0.58174000

H 2.54106400 3.36244500 -0.21458100

H -0.20862200 5.53905600 0.86972900

H -1.42928700 4.61747700 -0.01326800

H -1.62634000 4.29501600 2.44709900

H 0.03383900 3.70825000 2.52519100

H -1.31397100 0.46016200 2.51384400

H -1.67830500 1.81411500 3.62522700

H -0.00503400 1.51751800 3.06175500

H -2.88638800 3.20848900 0.57199900

H -3.36023900 2.21937400 1.94837700

H -2.60134600 0.16357200 0.71514600

H -3.92197000 1.01958100 -0.09446600

H -1.52688500 -0.90017500 -1.00820900

H -1.32897200 -0.09885700 -2.57167700

H -2.97393300 -0.48603700 -1.97379700

H -2.48425200 3.16256800 -1.50066900

H -3.47085000 1.94409300 -2.35121200

H -1.76285900 2.20943400 -2.81597900

H 1.04879500 -2.49436400 -1.42475900

H 0.14119000 -3.27652700 -3.55168900

H -0.09044600 -1.72251900 -5.49962500

H 0.65549000 0.65657500 -5.24587600

H 1.60358800 1.44546900 -3.11006800

H 3.02006700 -2.49225200 -0.61391100

H 3.50236000 -4.07288300 1.20154600

H 3.20015900 -3.40590600 3.59276900

H 2.41426100 -1.08352900 4.11431600

H 1.85405100 0.47933800 2.29014300

C -0.96166800 -3.72174900 0.60491000

C -0.49585800 -2.44223500 1.27433000

F -1.89198400 -3.48379800 -0.33876600

F 0.07751300 -4.33368400 0.01341700

F -1.49942100 -4.58266800 1.48727300

H -0.08852900 -1.75533300 0.51785900

F -1.57529600 -1.84990900 1.90595300

H 0.28836300 -2.67814100 2.00870300

TS-5

SCF (RB3PW91) = -1809.14900551

E(SCF)+ZPE(0 K)= -1808.597268

H(298 K)= -1808.561505

G(298 K)= -1808.666387

Lowest imaginary frequency = -303.4298 cm-1

P 1.37283800 -0.70689500 -0.85563500

Li -0.62718400 0.86476800 0.65690700

N 0.62810800 2.58048000 1.09657900

N -2.14759300 1.94345600 1.75788200

N -1.98818600 0.94113600 -1.01829400

C 1.48309700 3.07918500 0.02039300

C 1.46111100 2.12877900 2.21238300

C -0.32728500 3.60959500 1.51836800

C -1.44050000 3.05600400 2.39639400

C -2.84105500 1.12123200 2.74714100

C -3.06147800 2.38799400 0.69916000

C -3.24974000 1.32155800 -0.36937400

C -2.16727300 -0.30111200 -1.77411600

C -1.49804600 1.98405900 -1.91726400

C 1.11026800 -1.79300500 -2.30079600

C 0.91971400 -3.17822200 -2.14165500

C 0.65100500 -3.99250600 -3.24096600

C 0.56144100 -3.43823700 -4.52215400

C 0.74787600 -2.06574900 -4.69622200

C 1.02341700 -1.25220300 -3.59400600

C 2.89364900 -1.38258200 -0.11690300

C 3.90687900 -2.00512900 -0.86903200

C 5.07585700 -2.45953500 -0.25885500

C 5.26673700 -2.30284000 1.11730800

C 4.26748400 -1.69117100 1.87795700

C 3.09481400 -1.24143300 1.26980000

H 2.13359600 2.26855200 -0.33624900

H 2.11164400 3.93111900 0.34944000

H 0.86843300 3.41190100 -0.82715800

H 0.85595000 1.63482800 2.98381700

H 2.01558500 2.96810900 2.67875900

H 2.19112400 1.39588400 1.84584900

H 0.17958300 4.43827200 2.05568800

H -0.75779300 4.05729300 0.60922000

H -2.12877300 3.87984300 2.67561700

H -1.01560800 2.68418200 3.33995800

H -3.34274400 0.27789400 2.25702300

H -3.59768100 1.70194000 3.31198900

H -2.11563300 0.69395000 3.45170300

H -2.65954400 3.30165300 0.23946000

H -4.04955700 2.66965400 1.11455900

H -3.66914800 0.41473800 0.09008700

H -3.99457800 1.67290800 -1.11352300

H -2.43424400 -1.12149000 -1.09823300

H -1.23190000 -0.56960000 -2.27935800

H -2.95702400 -0.19924100 -2.54543000

H -1.36750100 2.93498400 -1.38432100

H -2.19072100 2.16135100 -2.76492800

H -0.51837800 1.68684700 -2.31831800

H 0.98187300 -3.61465300 -1.14129400

H 0.50304200 -5.06655800 -3.09811600

H 0.34774700 -4.07742900 -5.38301700

H 0.68486100 -1.62635900 -5.69565200

H 1.17477000 -0.17788200 -3.73478600

H 3.77413600 -2.13811100 -1.94595800

H 5.84798800 -2.94105400 -0.86595400

H 6.18410300 -2.65944800 1.59273600

H 4.39593800 -1.57196100 2.95766500

H 2.31567200 -0.78395900 1.88466800

C -1.61019500 -2.44089500 1.80333300

C -0.26596700 -1.87208700 1.58821000

F -2.58727000 -1.80199400 1.08798100

F -1.62650800 -3.74037600 1.43662700

F -2.09605200 -2.41296000 3.09671600

H 0.43516000 -1.46023900 0.16042700

F -0.31083100 -0.50435500 2.07845500

H 0.44974600 -2.40550900 2.22882400

Int-6

SCF (RB3PW91) = -1809.16457350

E(SCF)+ZPE(0 K)= -1808.609417

H(298 K)= -1808.573240

G(298 K)= -1808.678531

Lowest frequency = 15.0493 cm-1

P 2.66517800 0.01806900 -1.09748300

Li -1.06177400 1.04401900 0.78786800

N -0.32264500 3.02579900 0.23562900

N -2.94439300 2.05058800 1.17119500

N -2.05973100 0.01403500 -0.79095200

C 0.50851900 3.20274700 -0.95000500

C 0.43207100 3.39651200 1.43460000

C -1.55421200 3.80969000 0.09949400

C -2.60550600 3.47321500 1.14599100

C -3.58584900 1.69414500 2.43468100

C -3.75701400 1.62457500 0.02600700

C -3.45232700 0.18294100 -0.35889300

C -1.65911600 -1.39087500 -0.74777300

C -1.83744600 0.55240400 -2.12938000

C 1.78597700 -0.16028200 -2.70413800

C 1.19676700 -1.35679400 -3.14097800

C 0.50138700 -1.40379200 -4.34986800

C 0.38239600 -0.25731900 -5.13920500

C 0.98235200 0.93354200 -4.72399600

C 1.69117000 0.97689200 -3.52193700

C 2.89883400 -1.71661900 -0.59068700

C 3.95602600 -2.45256900 -1.15064800

C 4.16138100 -3.78285200 -0.78561200

C 3.32609500 -4.38876700 0.15792300

C 2.28151100 -3.66012800 0.72966400

C 2.06452200 -2.33338400 0.35266400

H 1.44220400 2.63569300 -0.83899800

H 0.77748800 4.26572000 -1.11926100

H -0.01209900 2.83050100 -1.84306600

H -0.14479600 3.18157400 2.34227600

H 0.71757700 4.46840800 1.42777400

H 1.34732000 2.79094700 1.48935700

H -1.34337300 4.89949600 0.14338200

H -1.95687500 3.62015700 -0.90783200

H -3.49848100 4.11023800 0.97491400

H -2.22944000 3.74012600 2.14421600

H -3.86492800 0.63405000 2.42981200

H -4.49902700 2.29668200 2.61698100

H -2.87861400 1.83532000 3.26217600

H -3.55373400 2.28310600 -0.82980800

H -4.83907100 1.73241700 0.23819500

H -3.61128400 -0.46319500 0.51729400

H -4.16246500 -0.15769200 -1.14117700

H -1.78861300 -1.78941100 0.26639200

H -0.59695900 -1.47873600 -1.01383200

H -2.24310800 -2.01394600 -1.45564000

H -2.10943000 1.61515600 -2.17598000

H -2.42931200 0.01428900 -2.89744800

H -0.77712600 0.46490600 -2.39629200

H 1.27851100 -2.25757600 -2.52808700

H 0.04304900 -2.34183200 -4.67383100

H -0.16804400 -0.29495300 -6.08268300

H 0.90871100 1.83076300 -5.34412000

H 2.17668800 1.90727000 -3.21431500

H 4.62035700 -1.98276700 -1.88218000

H 4.98310100 -4.34807200 -1.23323300

H 3.49336200 -5.42906500 0.44892600

H 1.62372800 -4.12139400 1.47051300

H 1.23723500 -1.77825600 0.79693200

C -0.89206300 -1.11692400 3.03353700

C -0.24924600 0.12364400 2.53413600

F -2.25825700 -1.12395400 2.91272900

F -0.45352600 -2.19570500 2.32560500

F -0.68330600 -1.43767600 4.35228600

H 1.51937500 0.27505100 -0.29108100

F -0.55985600 1.14447900 3.51352200

H 0.83759300 -0.05221600 2.63431700

TS-6

SCF (RB3PW91) = -1809.14598180

E(SCF)+ZPE(0 K)= -1808.592219

H(298 K)= -1808.556292

G(298 K)= -1808.659372

Lowest imaginary frequency = -356.8229 cm-1

P 2.89313900 -0.40296700 -0.71432900

Li -0.65540000 0.51750400 1.31914800

N -0.02692700 2.70269200 0.70071000

N -2.57055600 1.42947300 1.60379800

N -1.63639800 -0.43212700 -0.55918300

C 0.72426700 2.83749600 -0.54187300

C 0.73872900 3.30597200 1.79075800

C -1.34677500 3.32723900 0.57293800

C -2.33052700 2.87296100 1.64218500

C -3.15955800 0.95517200 2.85461800

C -3.38609200 1.01350600 0.45581800

C -3.00560800 -0.37432700 -0.03596800

C -1.21082800 -1.82327100 -0.70198700

C -1.52569700 0.23220100 -1.85376000

C 2.14769700 -0.44170100 -2.39576300

C 1.61956700 -1.59414000 -2.99633100

C 1.02733200 -1.52767600 -4.25867000

C 0.95161200 -0.31049900 -4.93888200

C 1.49592600 0.83865900 -4.36033800

C 2.10342400 0.76916100 -3.10599100

C 2.89978200 -2.15968900 -0.23023900

C 3.89610900 -2.99743600 -0.76010600

C 3.94930400 -4.34556600 -0.40788000

C 3.02251000 -4.86997100 0.49799200

C 2.04124400 -4.04065100 1.04350100

C 1.97348000 -2.69489800 0.67725200

H 1.72550200 2.40360100 -0.41454300

H 0.85438300 3.89777600 -0.84428800

H 0.22426100 2.30305700 -1.35943900

H 0.19976400 3.22072600 2.74029500

H 0.95279700 4.37723700 1.59349100

H 1.69159400 2.77298800 1.90957000

H -1.27181800 4.43513500 0.59841500

H -1.74221500 3.07680900 -0.42291200

H -3.27240400 3.45101900 1.53680700

H -1.92868800 3.11313400 2.63707000

H -3.27831000 -0.13547400 2.82232600

H -4.15024400 1.41604300 3.04527000

H -2.48971200 1.18432500 3.69232900

H -3.26370600 1.73851400 -0.36034200

H -4.46410700 1.03041800 0.71077300

H -3.06783300 -1.08235600 0.80313000

H -3.74017000 -0.71076500 -0.79875800

H -1.26506000 -2.33304500 0.26741600

H -0.17049300 -1.85521400 -1.04872600

H -1.83375200 -2.37320600 -1.43786100

H -1.81350100 1.28911200 -1.78354700

H -2.17009200 -0.24421300 -2.62178200

H -0.48802600 0.18674600 -2.20912100

H 1.66494900 -2.55107200 -2.47135700

H 0.61476700 -2.43401900 -4.70957300

H 0.48014000 -0.25944100 -5.92355900

H 1.45798900 1.79241800 -4.89309900

H 2.54770300 1.66943800 -2.67239700

H 4.63344000 -2.59216100 -1.45973400

H 4.72398900 -4.98817600 -0.83440200

H 3.06998000 -5.92463200 0.78140500

H 1.31589300 -4.43736800 1.75812100

H 1.19091800 -2.06856000 1.10850500

C -0.03667500 -0.77364600 3.57558900

C 0.47563400 0.52073600 3.47262600

F -0.89580900 -1.20200600 2.22557800

F 0.84830400 -1.78022200 3.52248600

F -1.00374500 -1.03815800 4.46351900

H 1.71645100 -0.02717900 -0.00900900

F -0.22925500 1.49498800 4.19249600

H 1.55739800 0.64957300 3.51981600

HFC=CFF

SCF (RB3PW91) =	-376.229633107
E(SCF)+ZPE(0 K)=	-376.200296
H(298 K)=	-376.194874
G(298 K)=	-376.228054

Lowest frequency = 232.7980 cm-1

C 3.43008800 -2.18280000 -5.37328600

C 3.67756200 -0.91544200 -5.04810800

F 4.38276600 -3.04471800 -5.67738800

F 2.23310000 -2.72826100 -5.43237600

F 2.67633000 -0.07781600 -4.74677000

H 4.68541300 -0.50178700 -5.01083400

$HPPh_2$

SCF (RB3PW91) =	-804.746586856
E(SCF)+ZPE(0 K)=	-804.554897
H(298 K)=	-804.542901
G(298 K)=	-804.595581

Lowest frequency = 5.5709 cm-1

P 2.41599200 -0.09751700 -1.03083600

C 1.69726500 -0.15180400 -2.72391800

C 0.91693000 -1.21621300 -3.20194100

C 0.40064300 -1.18773800 -4.49728100

C 0.65324800 -0.09511000 -5.33199000

C 1.43067200 0.96685300 -4.86742500

C 1.95635800 0.93384900 -3.57367300

C 2.49051200 -1.87018400 -0.59415600

C 3.51415300 -2.65020100 -1.15821200

C 3.62842900 -4.00499700 -0.85001100

C 2.73226400 -4.59922200 0.04308900

C 1.71835200 -3.83171600 0.61854800

C 1.59341400 -2.47798400 0.29732700

H 0.71575900 -2.07495800 -2.55628200

H -0.20490100 -2.02353200 -4.85807900

H 0.24780100 -0.07549600 -6.34697700

H 1.63841200 1.82103300 -5.51725400

H 2.57927800 1.76087600 -3.22001200

H 4.22594300 -2.19178900 -1.85133500

H 4.42582300 -4.59956500 -1.30350900

H 2.82557500 -5.65986200 0.29006400

H 1.01254200 -4.29012500 1.31633200

H 0.78457800 -1.89171300 0.74154000

H 1.22215900 0.20518500 -0.31823900

TS-7

SCF (RB3PW91) = -1809.10431170

E(SCF)+ZPE(0 K)= -1808.548010

H(298 K)= -1808.512245

G(298 K)= -1808.615822

Lowest imaginary frequency = -388.4053 cm-1

P 2.34180300 4.63707900 3.51635200

Li 3.09352100 5.79332600 1.50488800

N 4.66718300 7.17104100 1.75367100

N 1.92293400 7.38202300 0.82920100

N 2.86516100 4.82593100 -0.33737800

C 6.01128000 6.74362300 1.37801600

C 4.62238400 7.48475900 3.18320900

C 4.22348800 8.29794500 0.92528400

C 2.73160400 8.57972700 1.06527300

C 0.62393400 7.45342500 1.49372900

C 1.78306200 7.04376200 -0.59296800

C 1.66440800 5.53930400 -0.79693600

C 2.57706900 3.40376100 -0.13944100

C 3.98736500 4.98829300 -1.25530700

C 3.70259400 3.87916300 4.44435600

C 3.65578000 3.53817200 5.81155800

C 4.72172900 2.88442800 6.42659000

C 5.86115000 2.53491400 5.69367300

C 5.91998100 2.84736100 4.33309400

C 4.85754600 3.51315100 3.72188000

C 1.55370000 5.75968300 4.71204100

C 2.27904100 6.47233500 5.69017700

C 1.65566400 7.40438300 6.51936400

C 0.28703300 7.66029800 6.39486100

C -0.44843200 6.96245300 5.43344300

C 0.17229400 6.02281800 4.61020400

H 6.03887300 6.46609600 0.31481100

H 6.29843500 5.86387800 1.97199400

H 6.76828100 7.53586200 1.54779600

H 3.59733100 7.70811800 3.50584400

H 5.27298700 8.34621200 3.43688700

H 4.95168400 6.60813500 3.75795200

H 4.79035300 9.22337300 1.15913900

H 4.45906400 8.05213300 -0.12231700

H 2.45380400 9.41445200 0.39048100

H 2.51784300 8.93073900 2.08485500

H 0.09987300 6.49235600 1.40259700

H -0.01476500 8.25729100 1.07537500

H 0.76511200 7.63347300 2.56740100

H 2.65732900 7.42438700 -1.14039100

H 0.90326400 7.54852600 -1.03841400

H 0.81512200 5.15916300 -0.20958300

H 1.44694700 5.31972900 -1.86256700

H 1.75312000 3.29314600 0.57549800

H 3.46096200 2.89944500 0.27748100

H 2.30677800 2.90148200 -1.09018400

H 4.87610600 4.48558800 -0.84706900

H 4.23679500 6.04926500 -1.39301700

H 3.77486900 4.55601600 -2.25431900

H 2.76569100 3.78602300 6.39481800

H 4.65986800 2.63418700 7.48966800

H 6.69408300 2.01992500 6.17934200

H 6.80247900 2.57695500 3.74603400

H 4.91779000 3.75880100 2.65585800

H 3.35105400 6.29309600 5.80296300

H 2.24654900 7.93875900 7.26916800

H -0.20154000 8.39046900 7.04535300

H -1.52224500 7.14343000 5.32920700

H -0.41279800 5.48244100 3.86322500

C 0.52343000 2.81968700 3.23495800

C -0.88717100 2.29875500 2.95009400

H -1.62460400 3.02271500 3.32158300

H -1.01340300 2.13649400 1.87331300

F -1.08535000 1.08498400 3.60226800

F 0.14120800 4.09708900 2.09494900

F 1.42074400 2.00156200 2.65781200

F 0.62892400 2.73669300 4.60294000

Int-8 ($Ph_2PCF_2CH_2F$)

SCF (RB3PW91) = -1181.03534614

E(SCF)+ZPE(0 K)= -1180.808895

H(298 K)= -1180.791782

G(298 K)= -1180.854656

Lowest frequency = 20.2473 cm-1

P 2.09937700 -0.49539200 -1.69480900

C 0.85487600 -1.52366300 -2.55564700

C -0.38092500 -0.99419400 -2.96235100

C -1.26439500 -1.76284800 -3.72071300

C -0.92520300 -3.06339100 -4.09914900

C 0.30829900 -3.59168600 -3.71424300

C 1.19204500 -2.82931200 -2.94986300

C 2.90030800 -1.67679800 -0.54976900

C 2.20179100 -2.67309000 0.15424600

C 2.87286800 -3.49816100 1.05462200

C 4.24710500 -3.33997100 1.26311700

C 4.94934200 -2.35576700 0.56688500

C 4.27855300 -1.52872700 -0.33754800

H -0.66424700 0.02270600 -2.68543000

H -2.22679800 -1.33843500 -4.01820700

H -1.61915900 -3.66223700 -4.69431500

H 0.58711200 -4.60707300 -4.00786200

H 2.15154000 -3.26050700 -2.65342900

H 1.12912000 -2.79989900 -0.00731500

H 2.32161300 -4.26932400 1.59889300

H 4.77042100 -3.98928700 1.96979800

H 6.02352400 -2.23097500 0.72460500

H 4.82787100 -0.75524400 -0.88108500

C 0.99907100 0.38379100 -0.42053900

C 1.76213800 0.93451000 0.77694400

H 1.06937200 1.55422400 1.36973100

H 2.13669700 0.09914400 1.38725300

F 2.82475500 1.70959400 0.35917000

F 0.35917000 1.42139000 -1.05814300

F 0.00763100 -0.41127700 0.09785000

9.1 IR Spectra

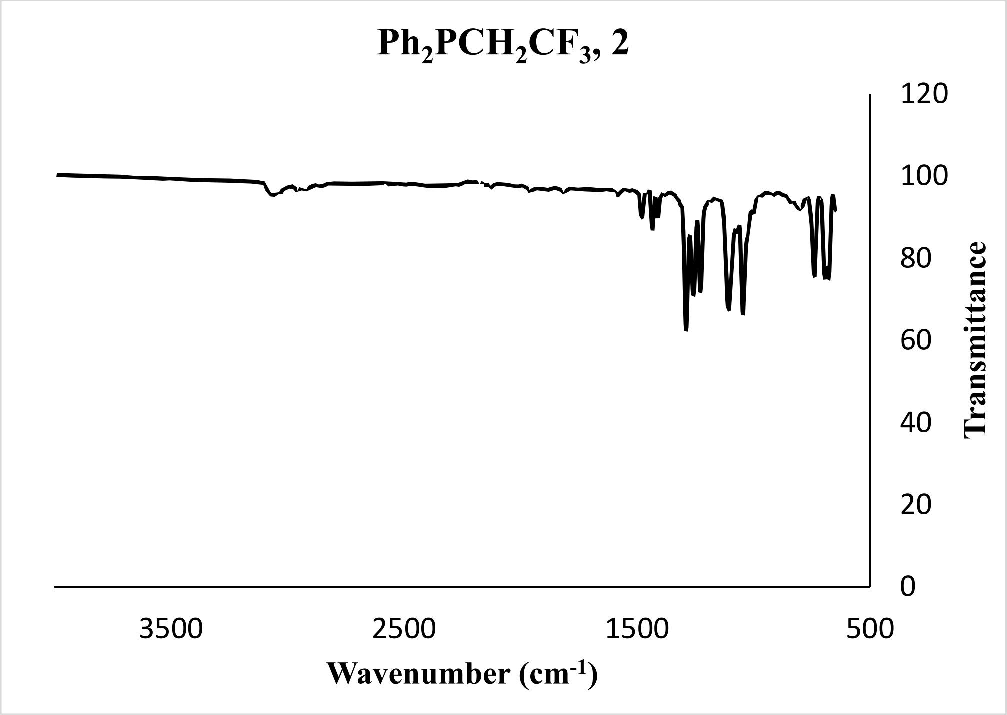

IR Spectra of **2**

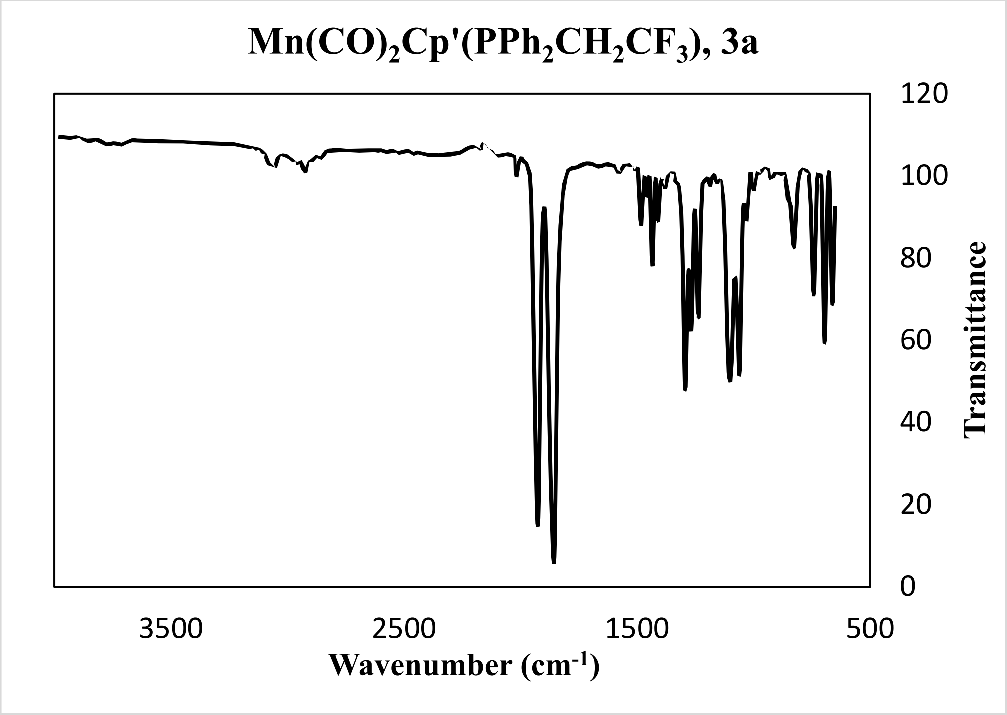

IR Spectra of **3a**

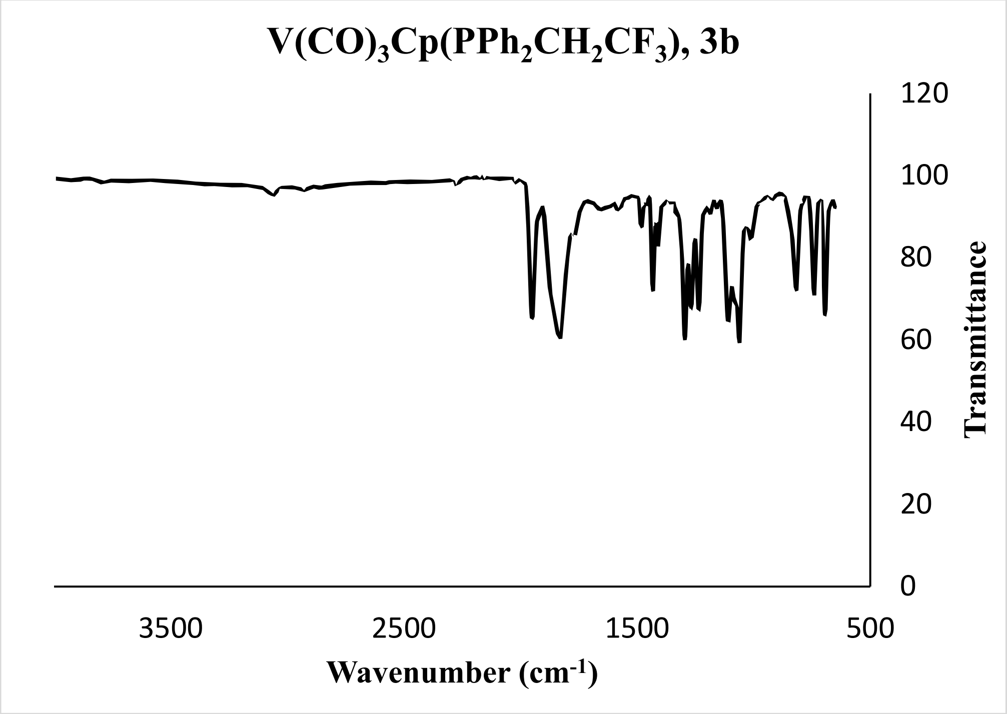

IR Spectra of **3b**

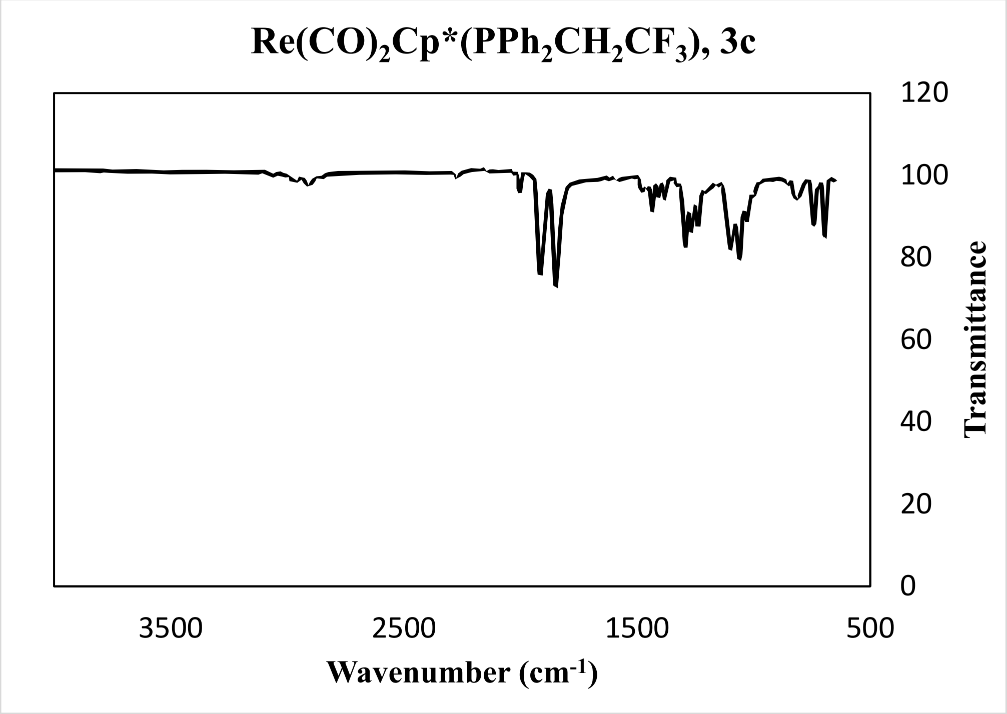

IR Spectra of **3c**

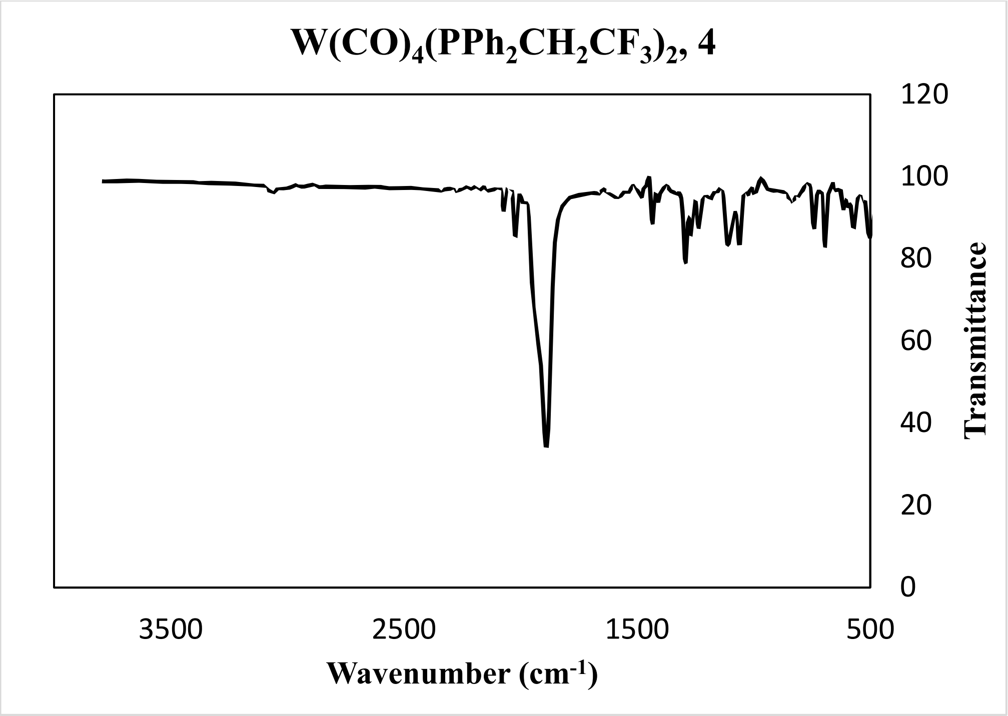

IR Spectra of **4**

10.1 NMR Spectra

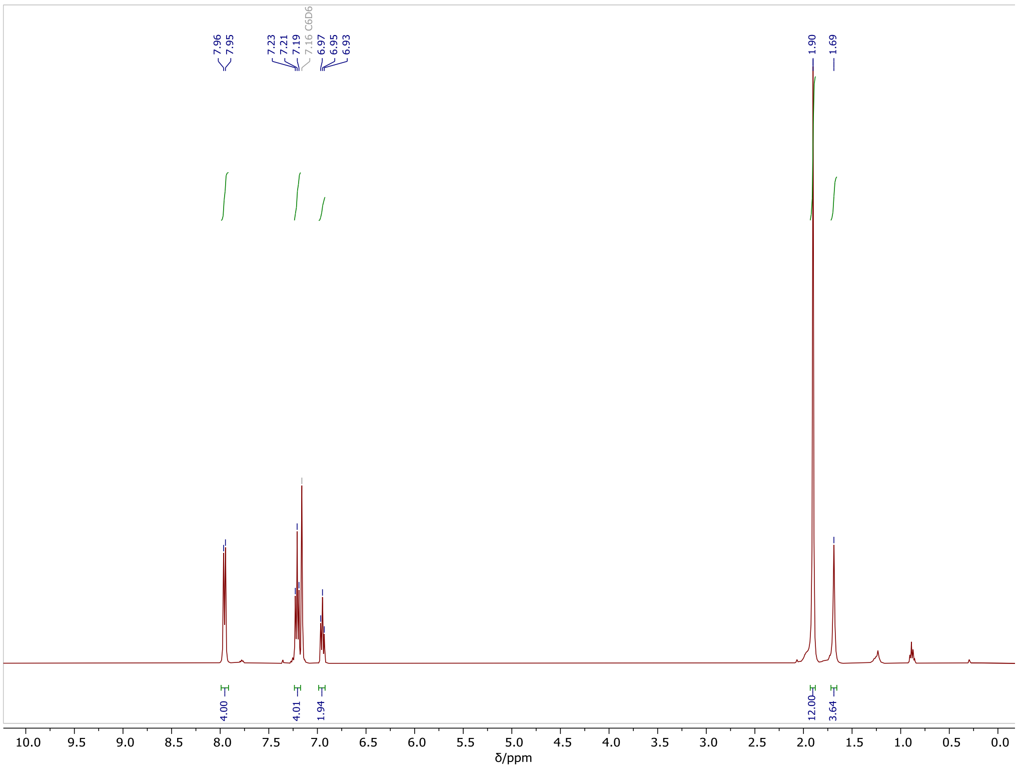

^{1}H NMR Spectra of **1·TMEDA**

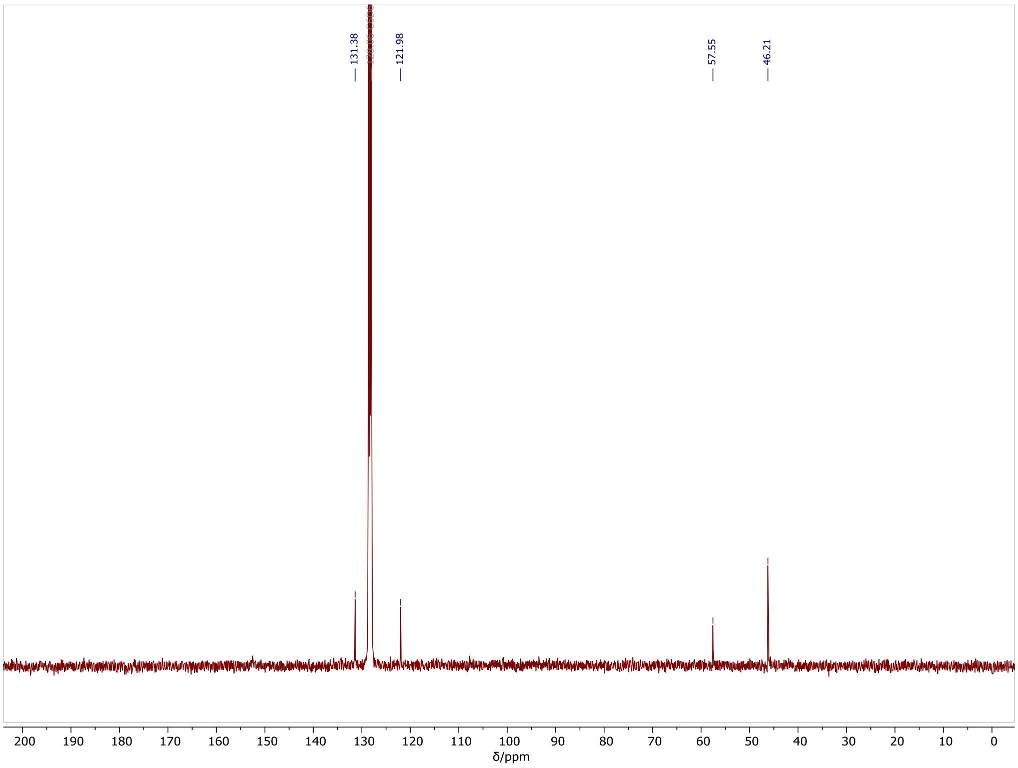

^{13}C NMR Spectra of **1·TMEDA**

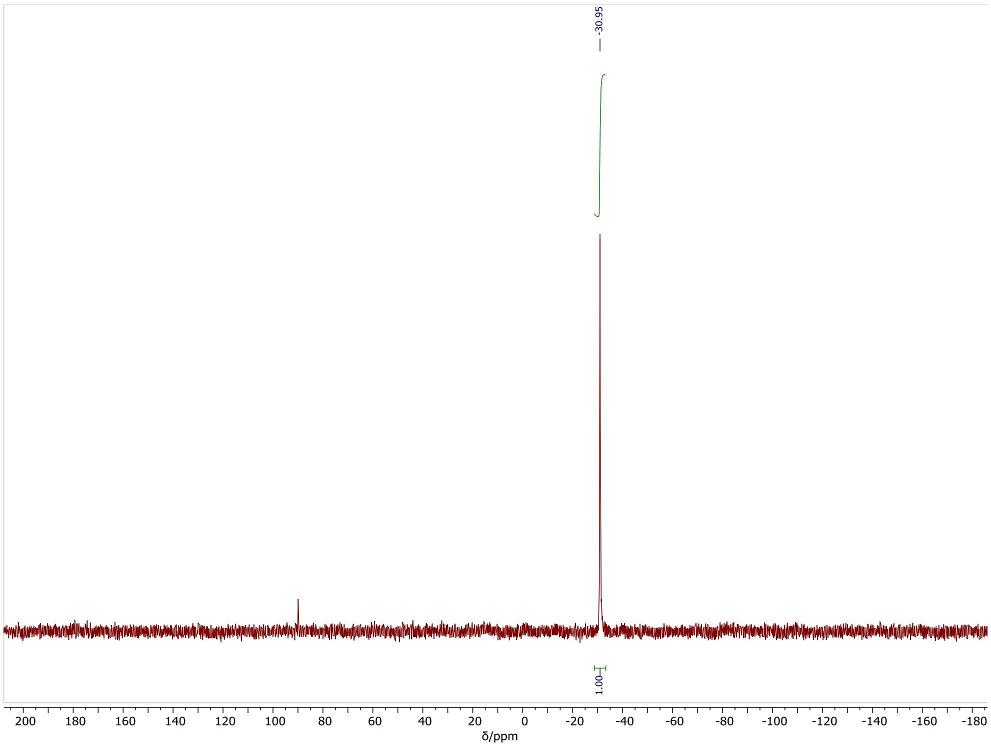

$^{31}P\{^{1}H\}$ NMR Spectra of **1·TMEDA**

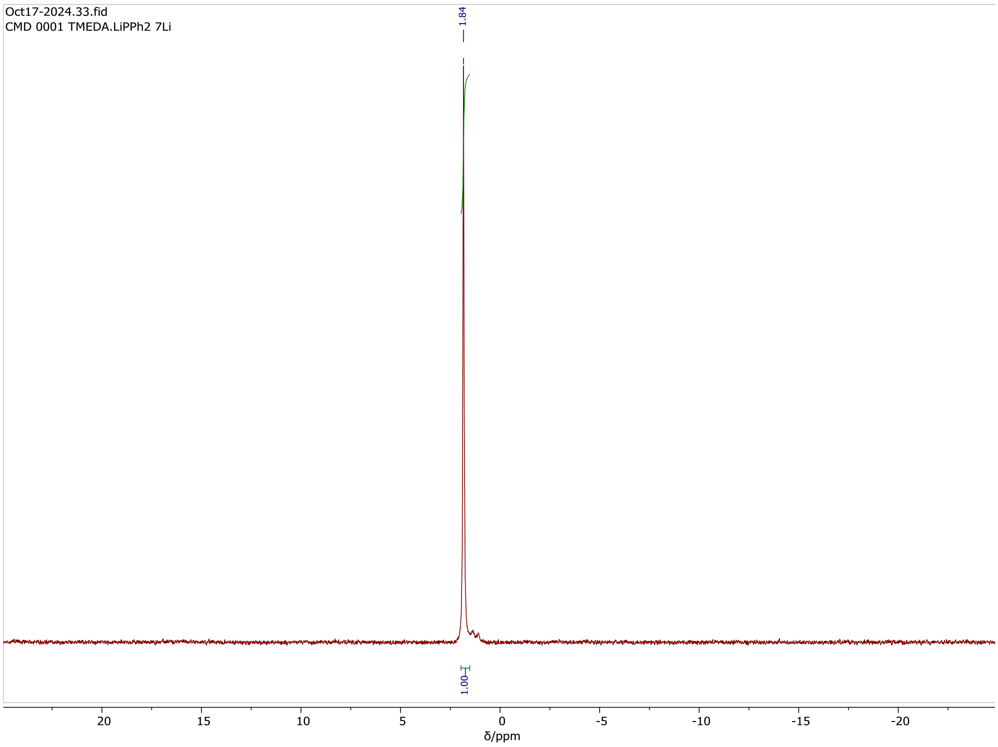

^{7}Li NMR Spectra of **1·TMEDA**

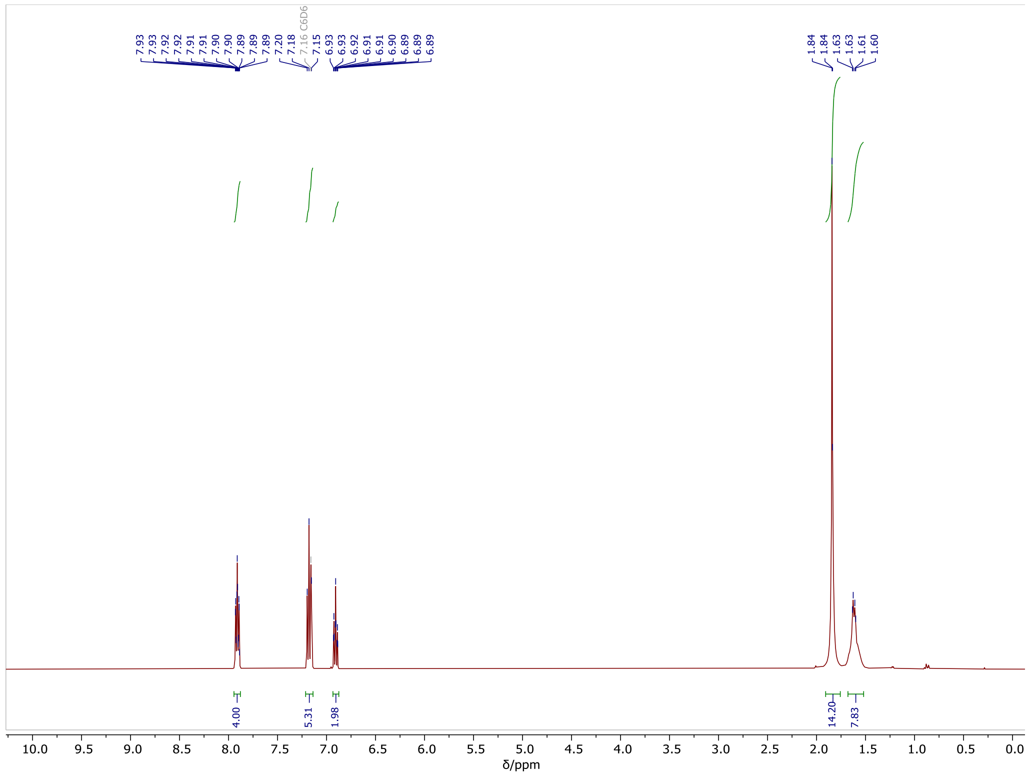

^{1}H NMR Spectra of **1·PMDETA**

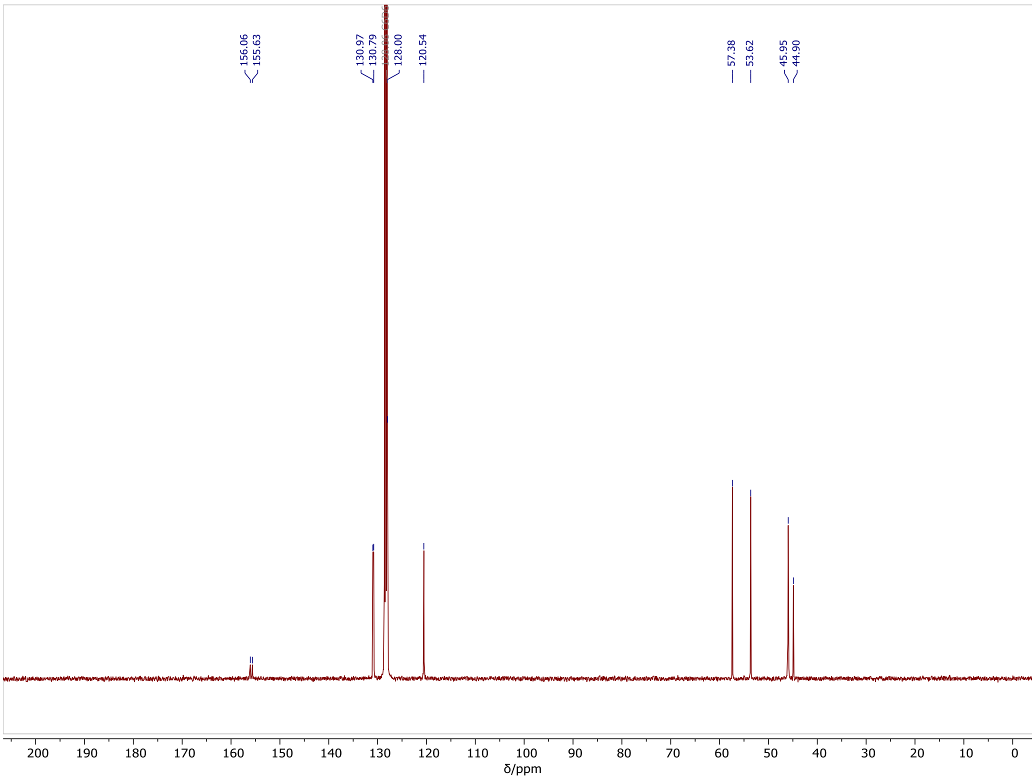

^{13}C NMR Spectra of **1·PMDETA**

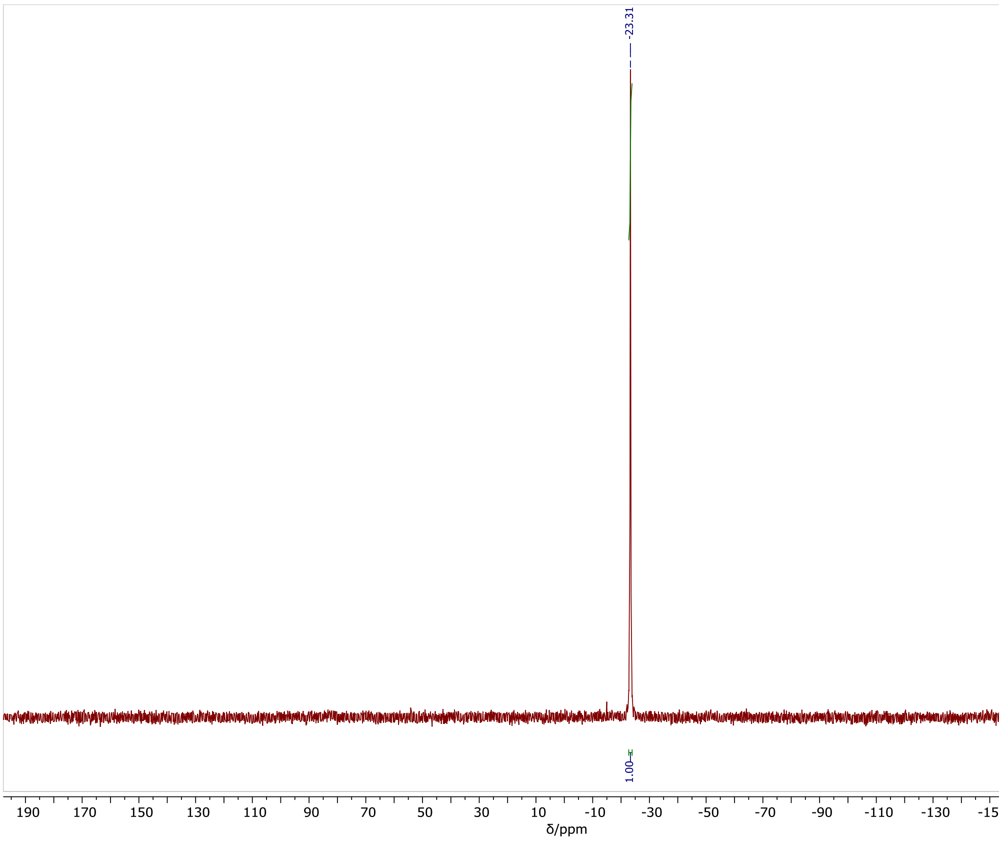

^{31}P NMR Spectra of **1·PMDETA**

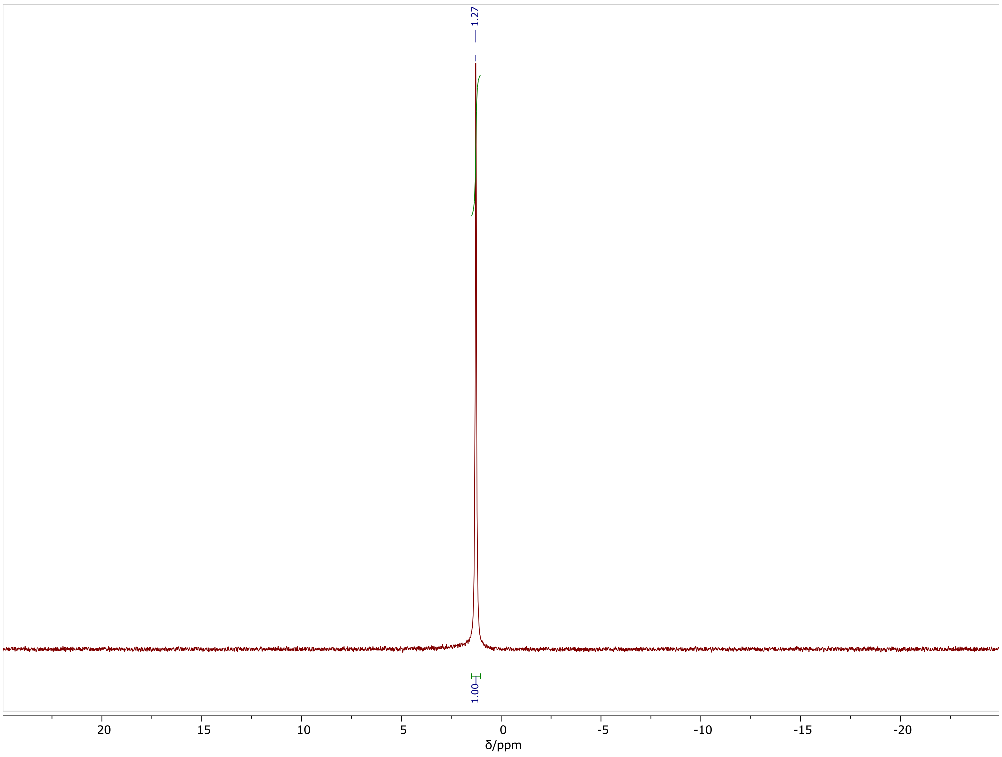

^{7}Li NMR Spectra of **1·PMDETA**

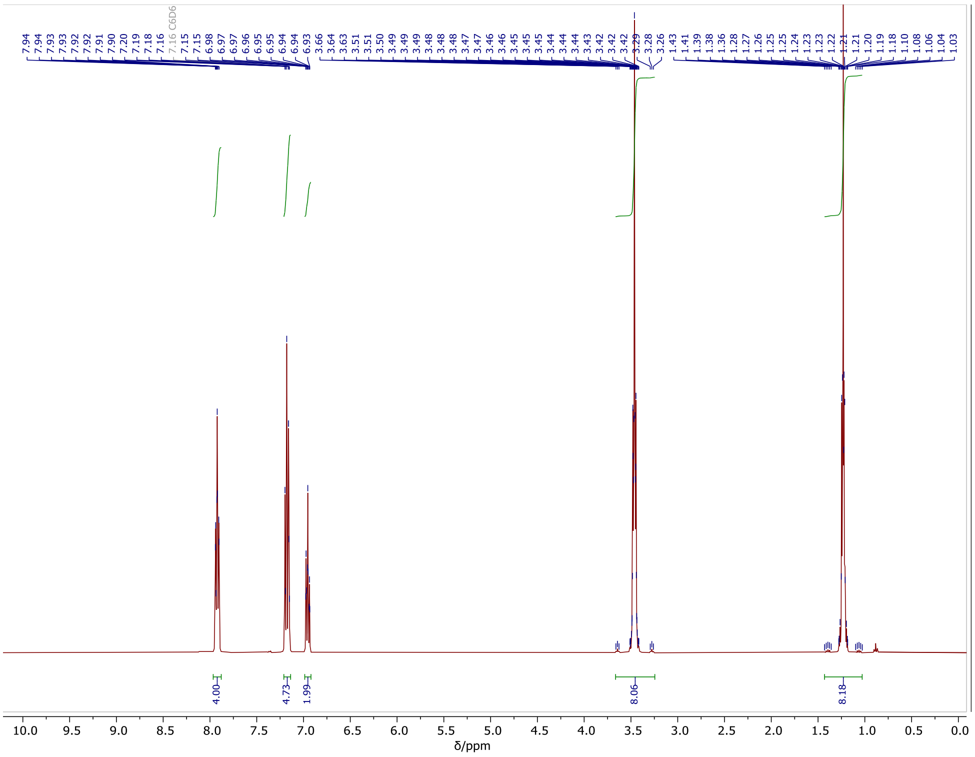

^{1}H NMR Spectra of **1·(THF)₂**

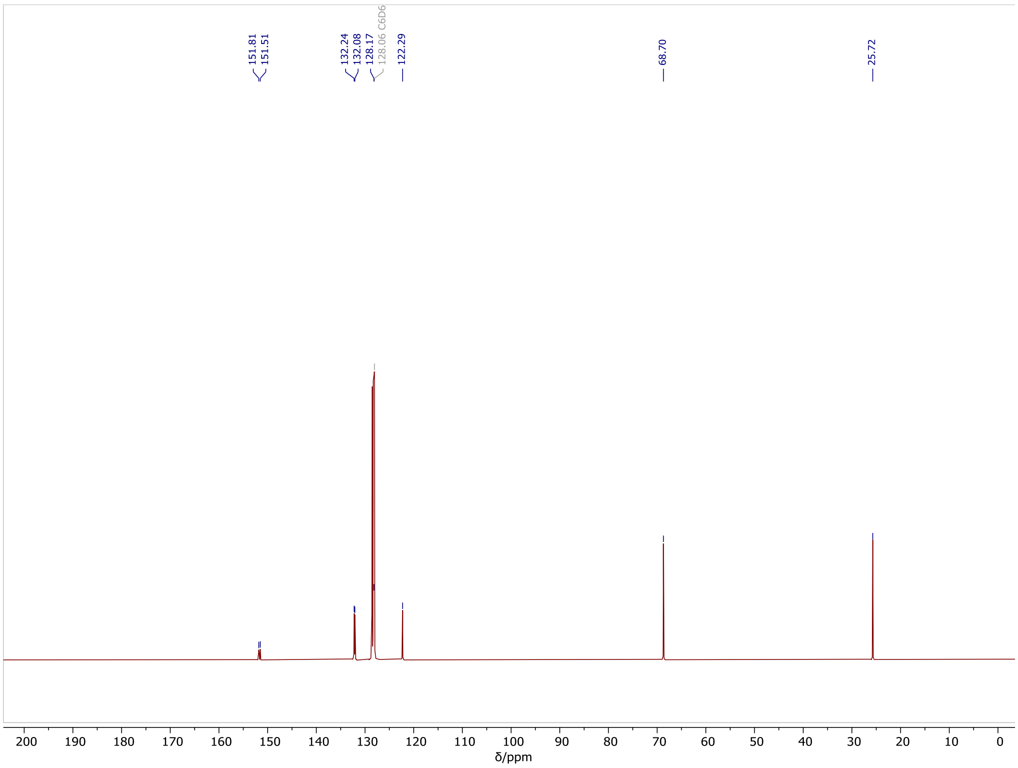

^{13}C NMR Spectra of **1·(THF)₂**

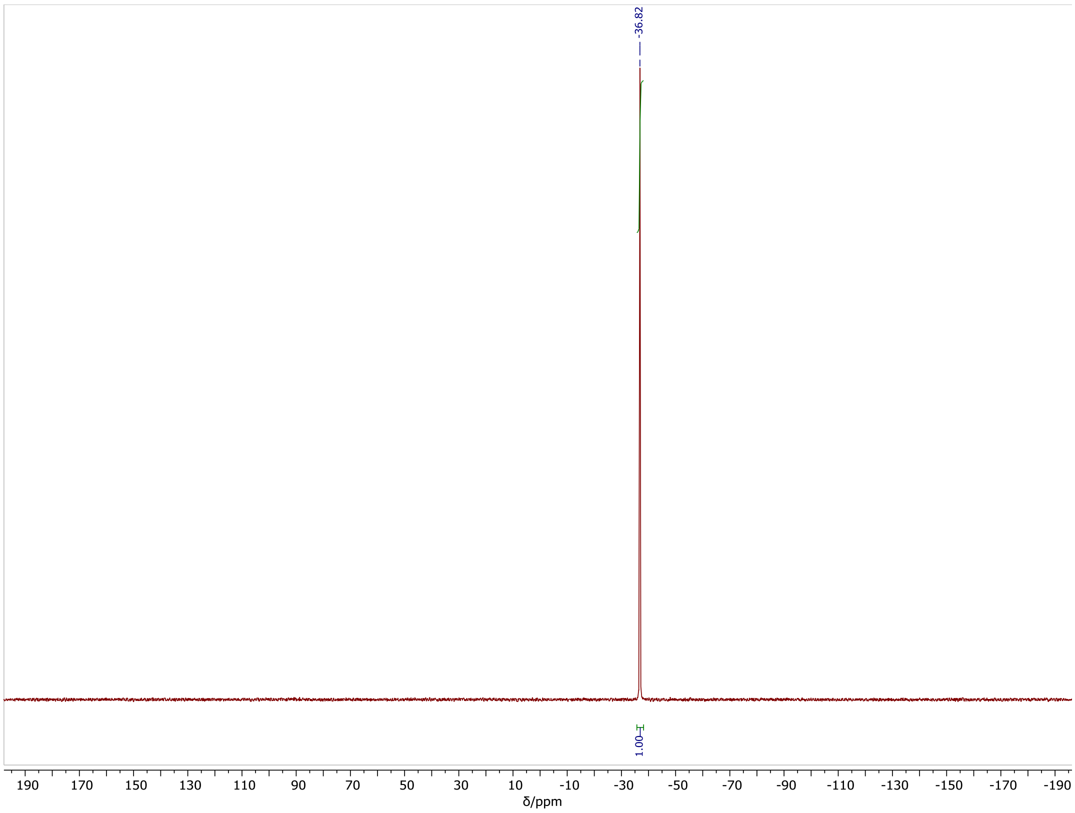

^{31}P NMR Spectra of **1·(THF)₂**

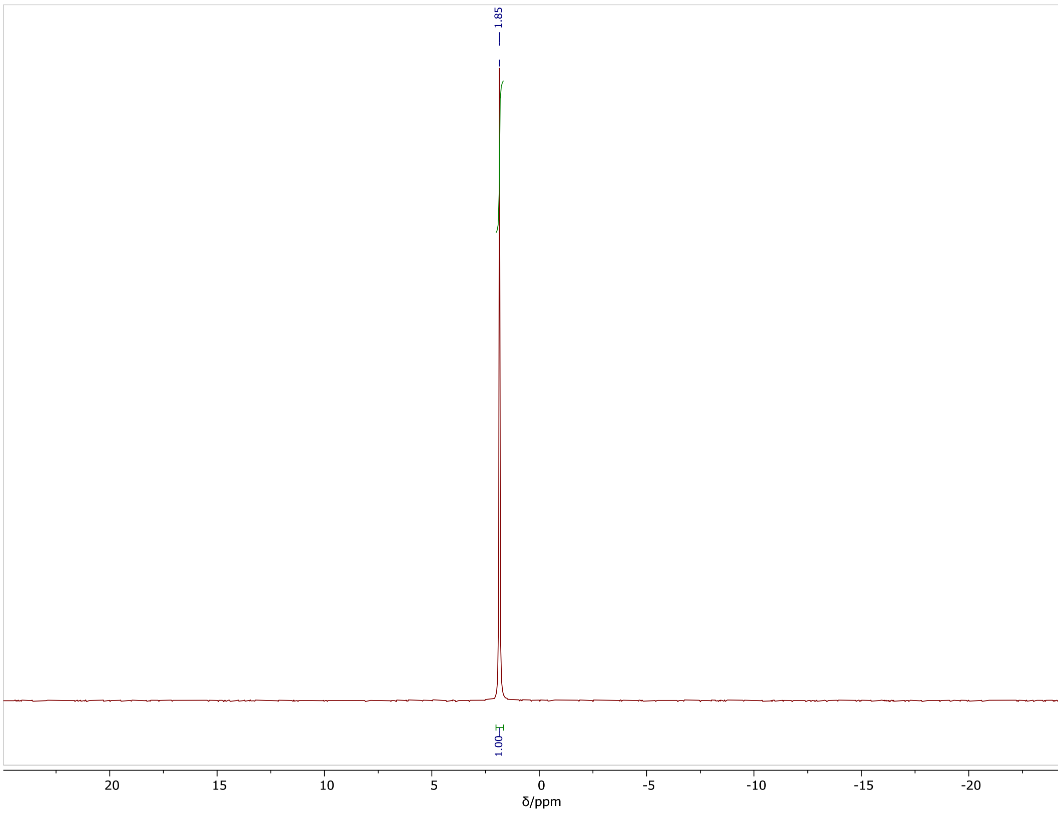

^{7}Li NMR Spectra of **1·(THF)₂**

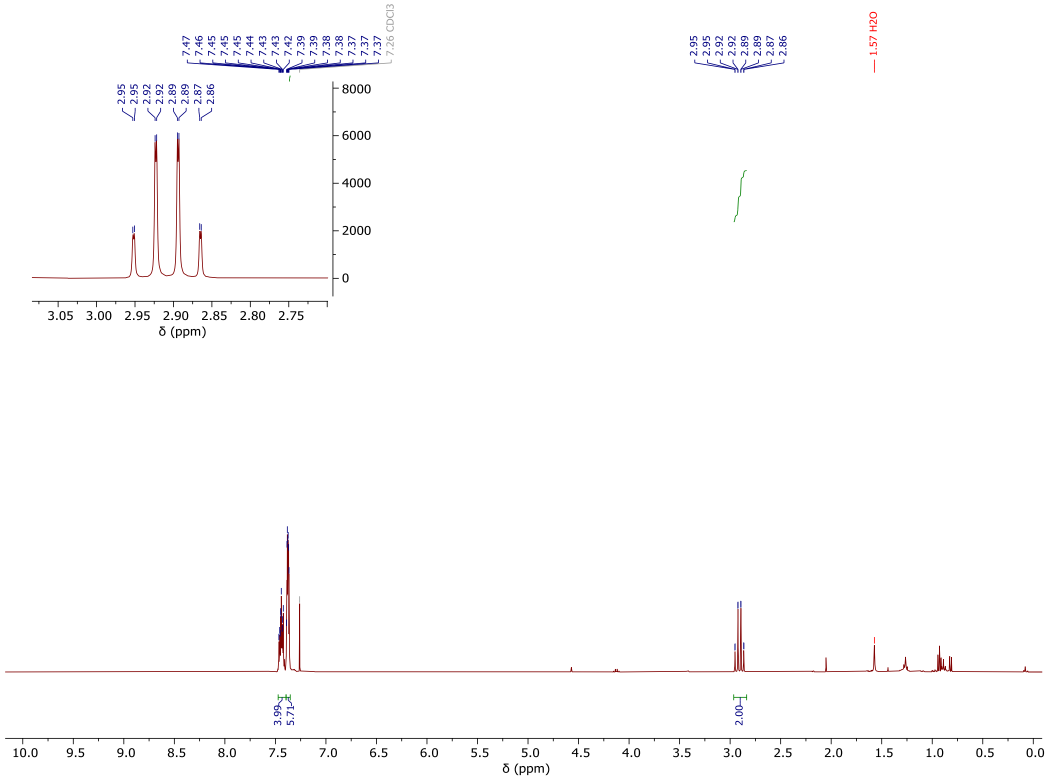

^{1}H NMR Spectra of **2**

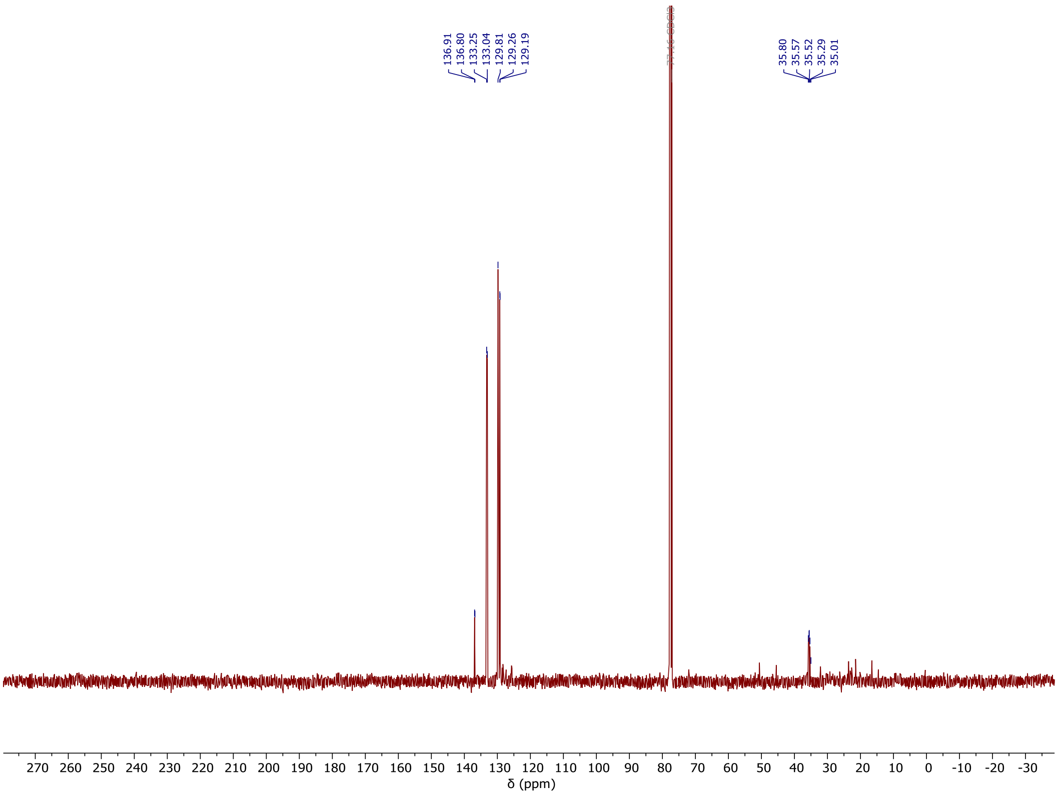

^{13}C NMR Spectra of **2**

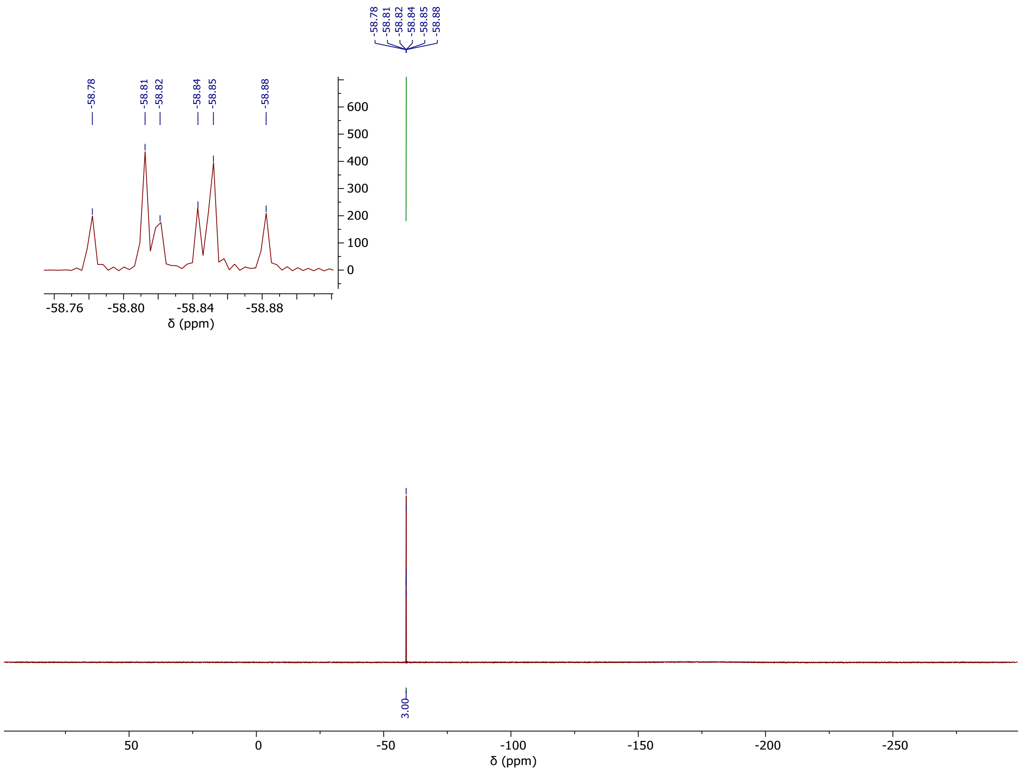

^{19}F NMR Spectra of **2**

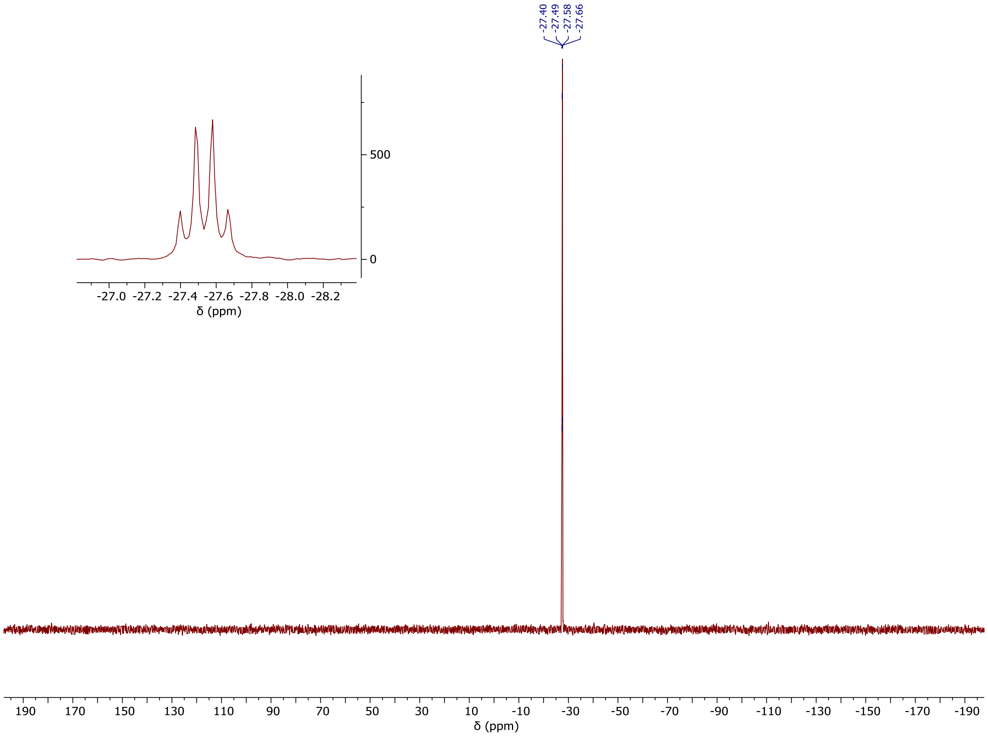

^{31}P NMR Spectra of **2**

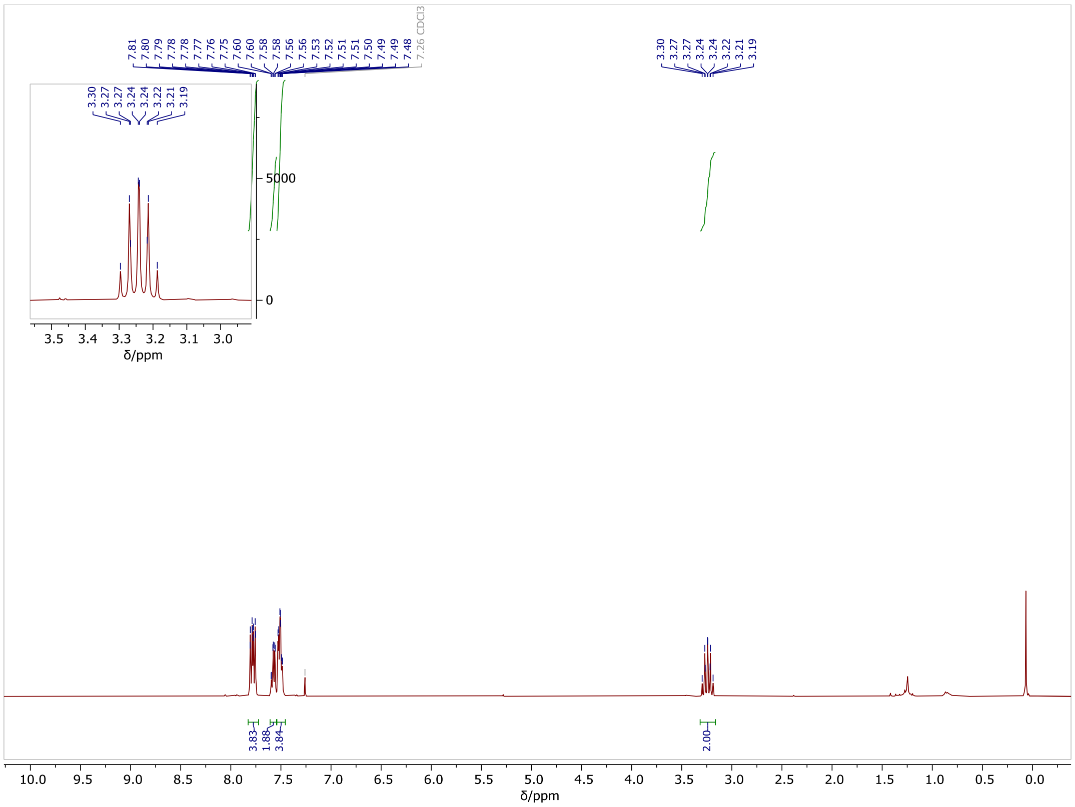

^{1}H NMR Spectra of **2·O**

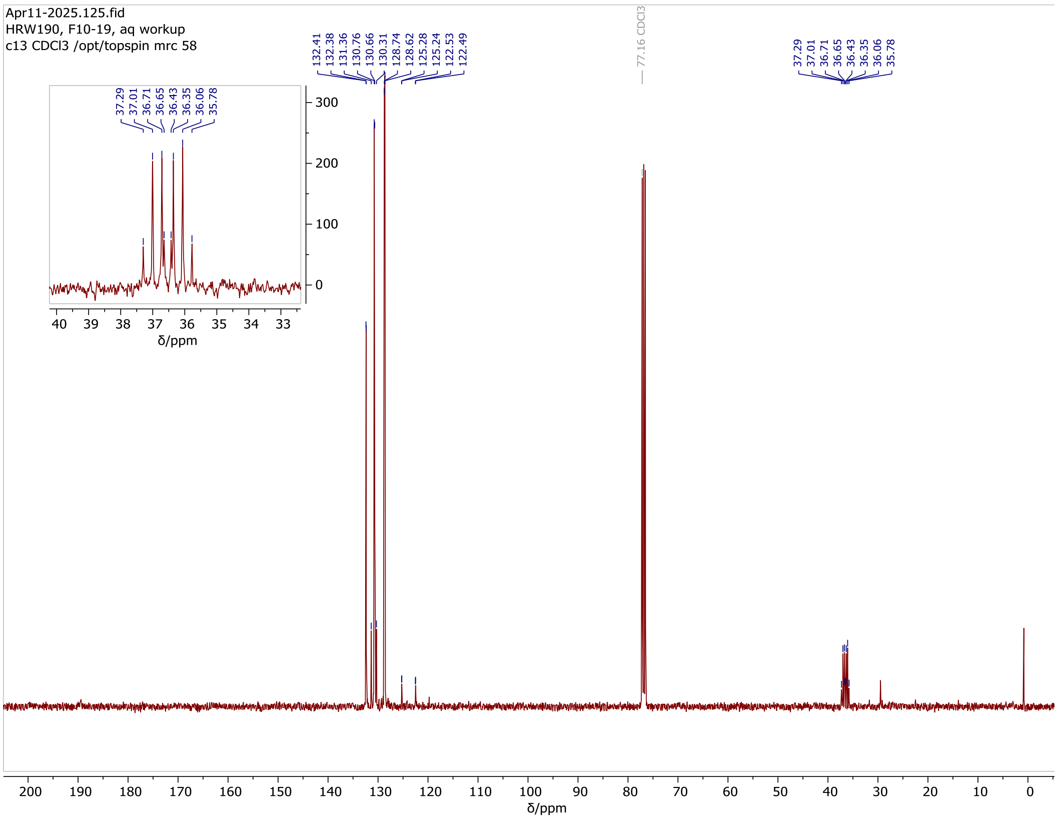

^{13}C NMR Spectra of **2·O**

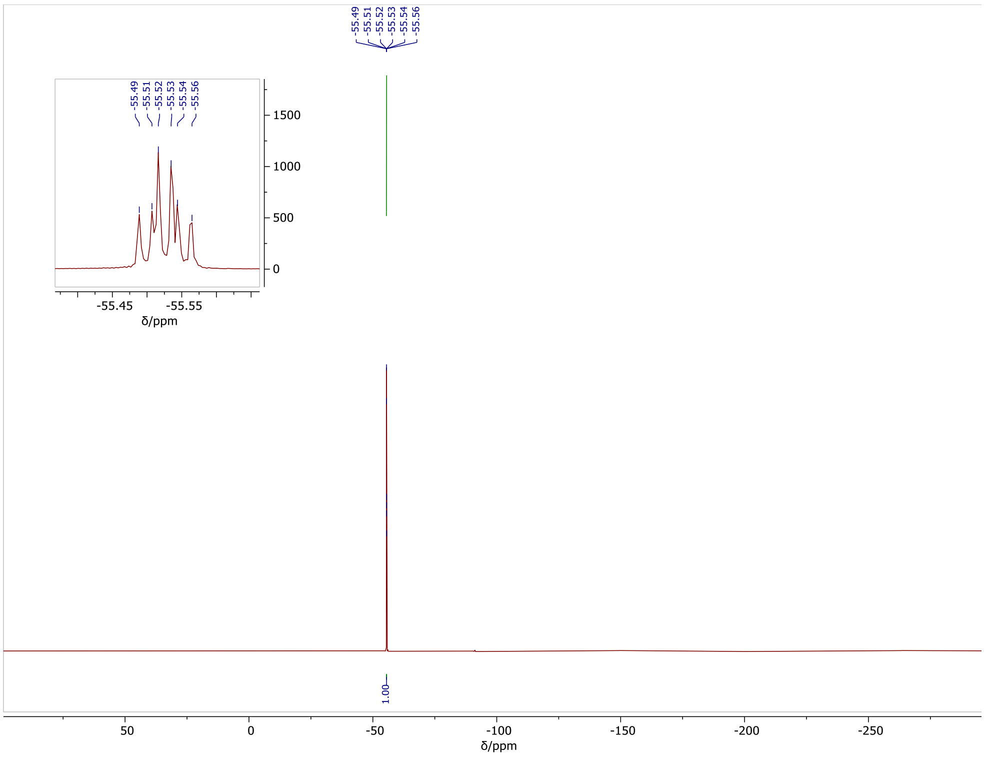

^{19}F NMR Spectra of **2·O**

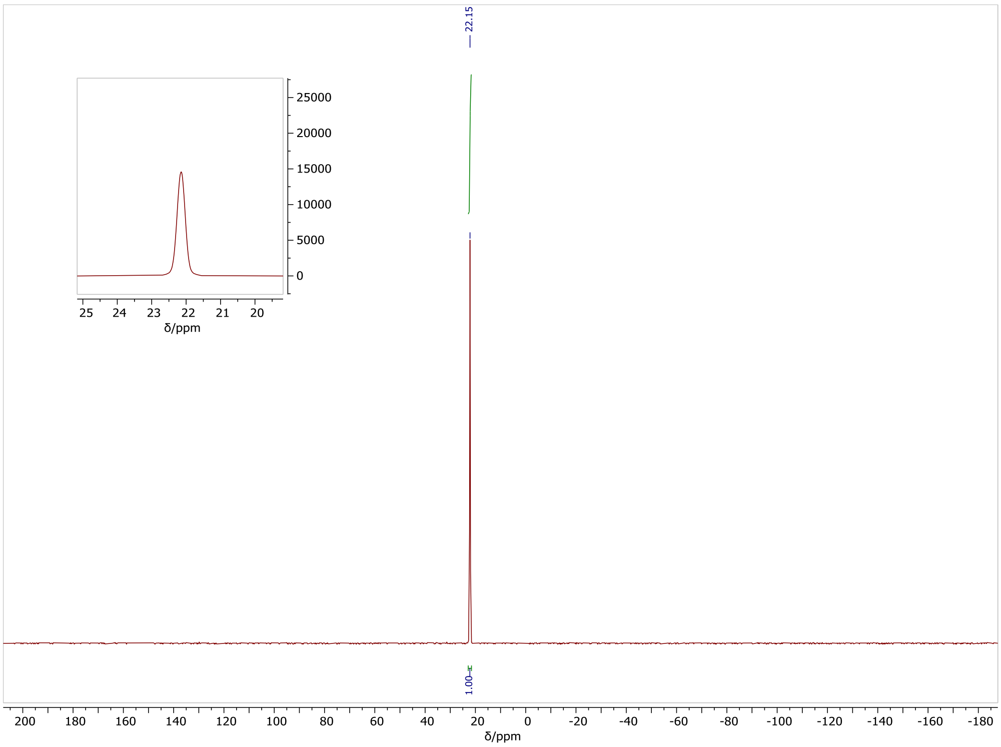

^{31}P NMR Spectra of **2·O**

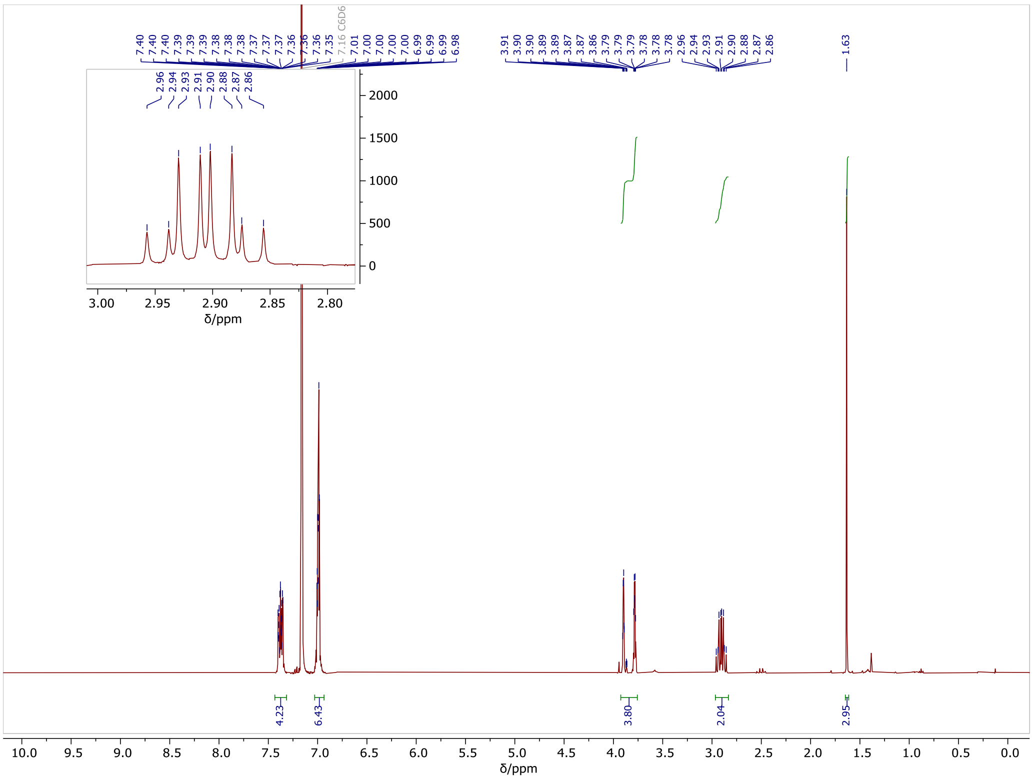

^{1}H NMR Spectra of **3a**

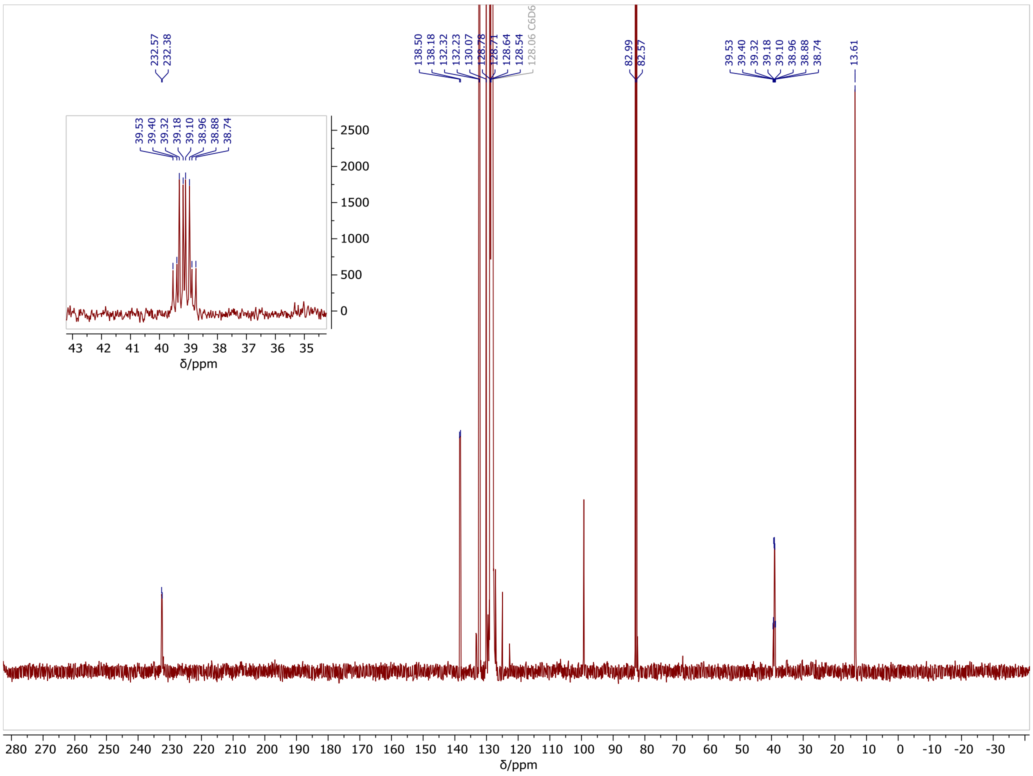

^{13}C NMR Spectra of **3a**

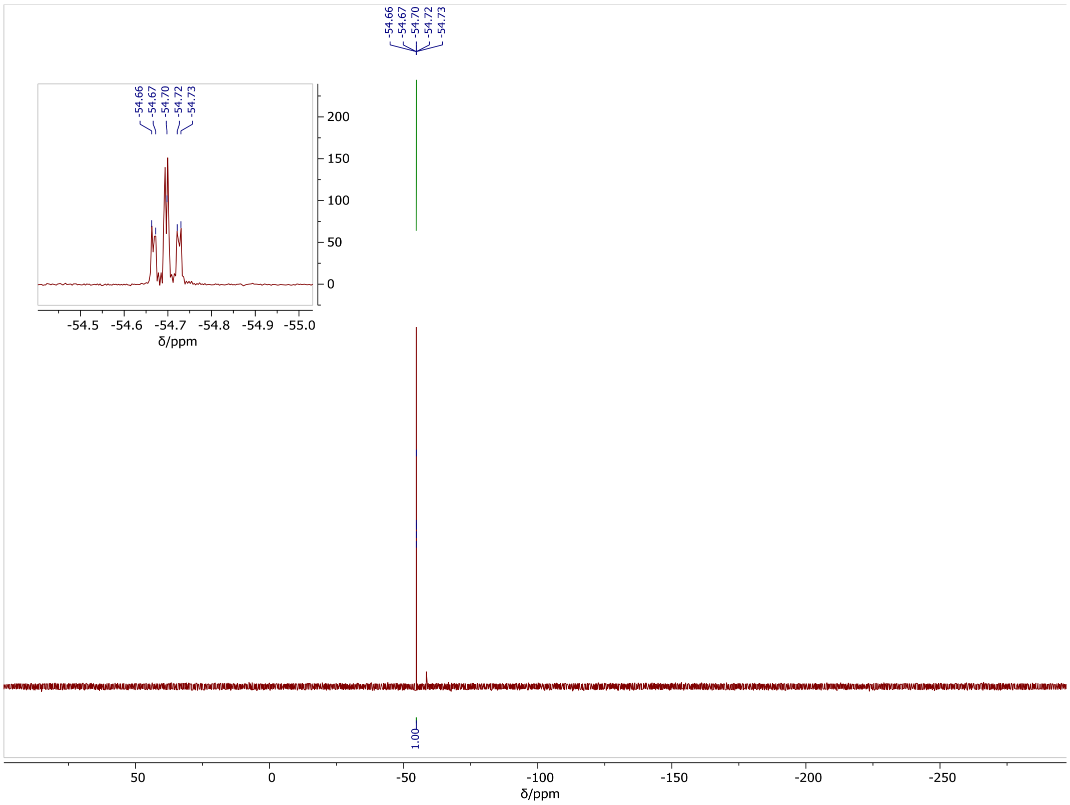

^{19}F NMR Spectra of **3a**

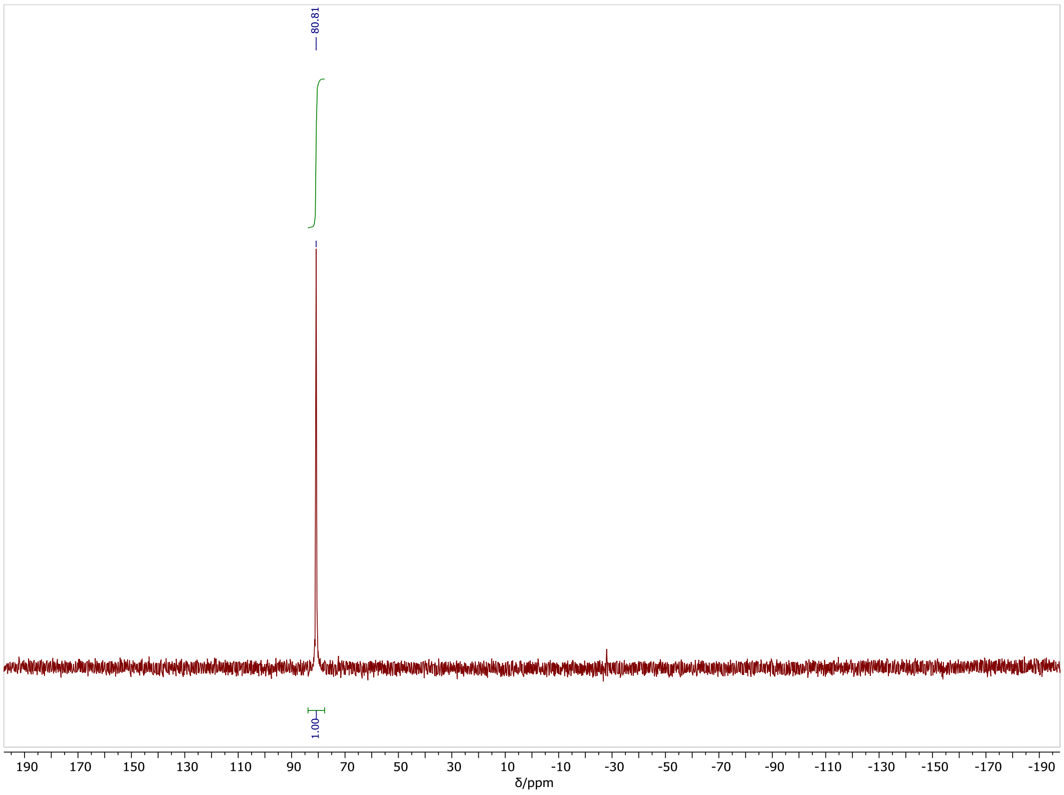

^{31}P NMR Spectra of **3a**

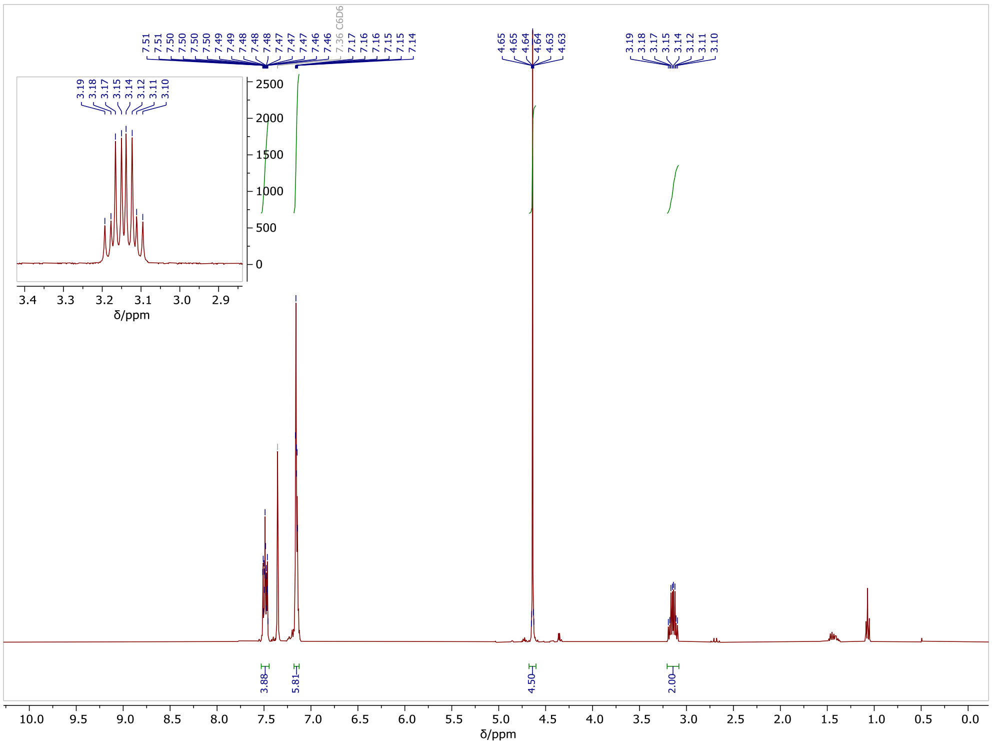

^{1}H NMR Spectra of **3b**

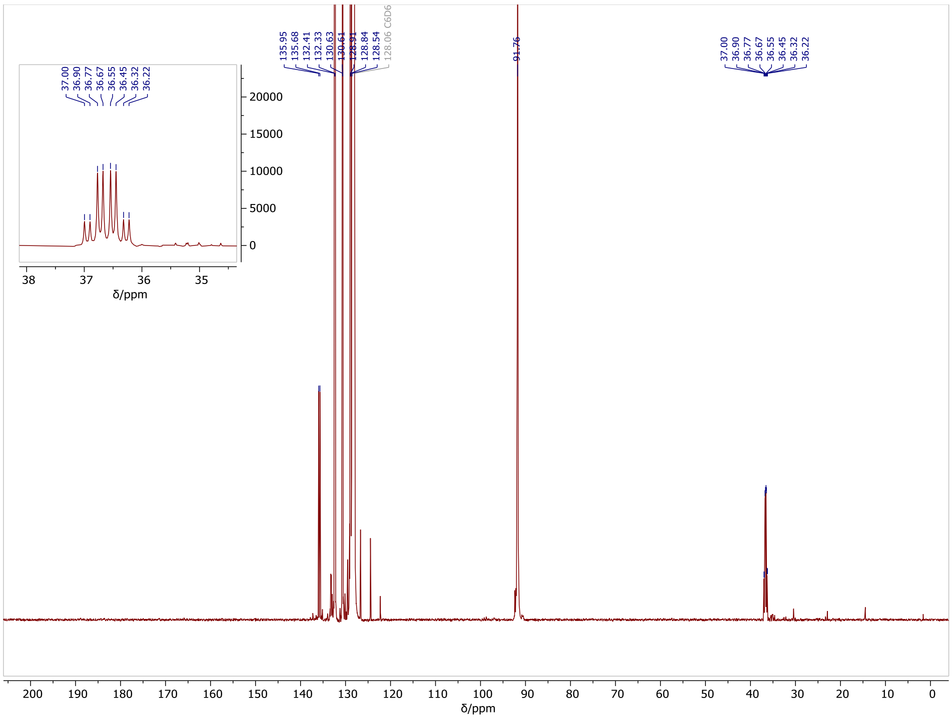

^{13}C NMR Spectra of **3b**

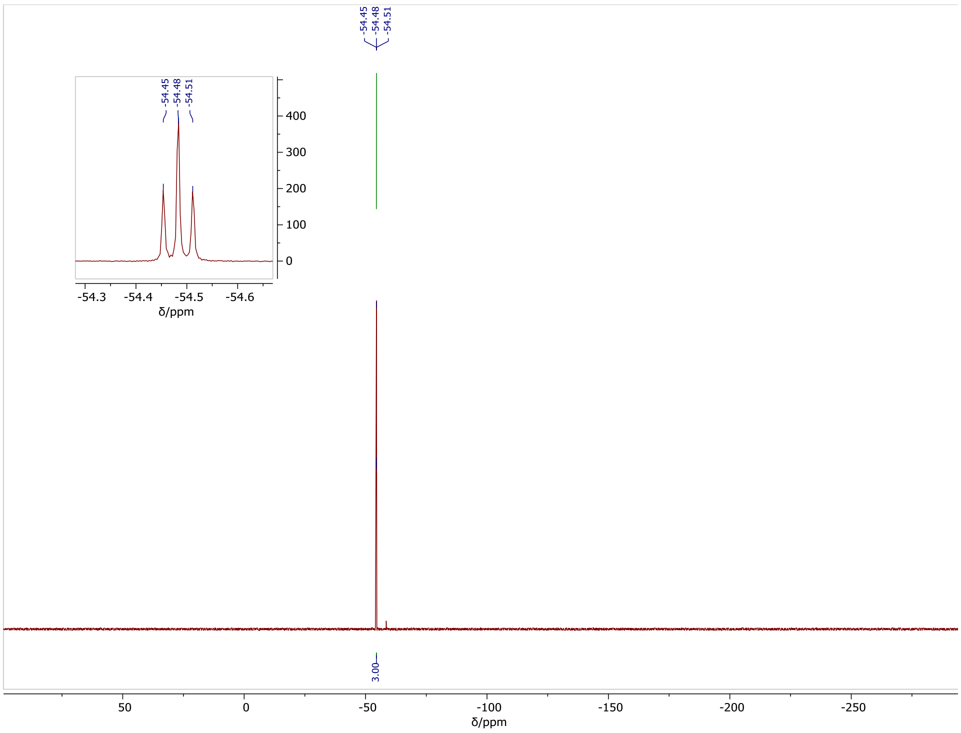

^{19}F NMR Spectra of **3b**

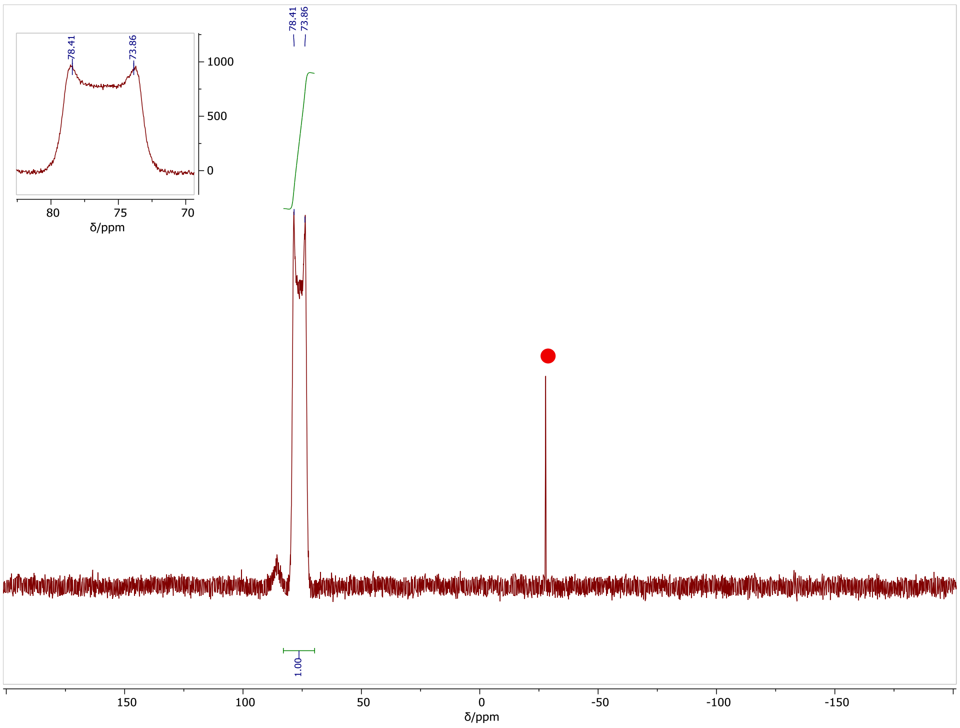

^{31}P NMR Spectra of **3b**, red dot labelled is starting material

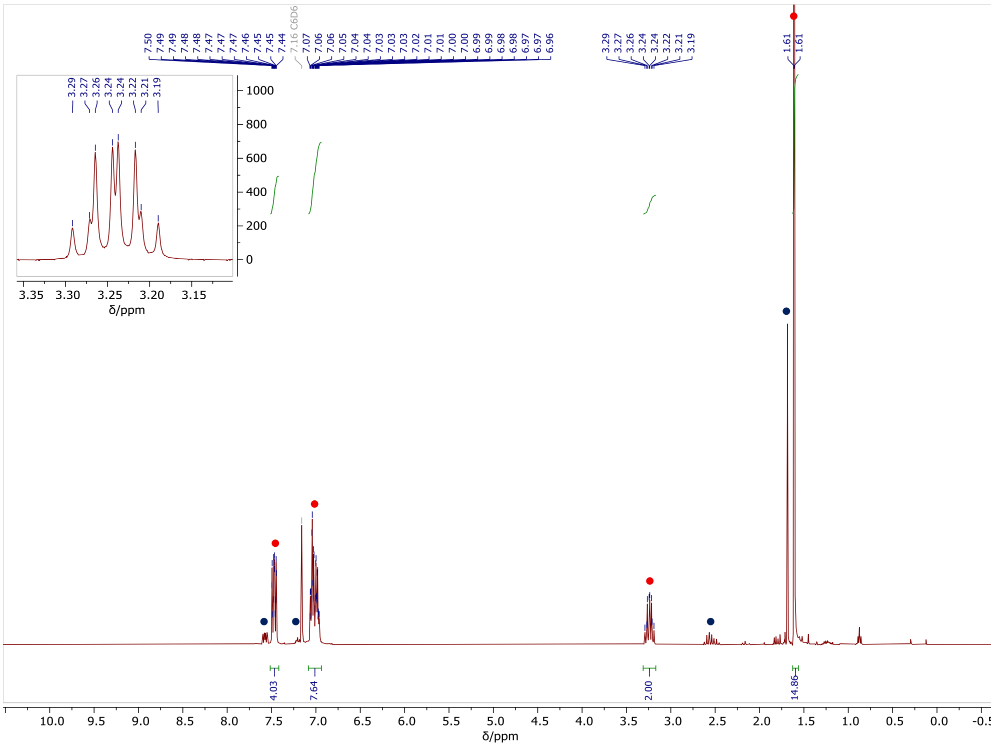

^{1}H NMR Spectra of **3c** (labelled in red), **3c'**(labelled in blue)

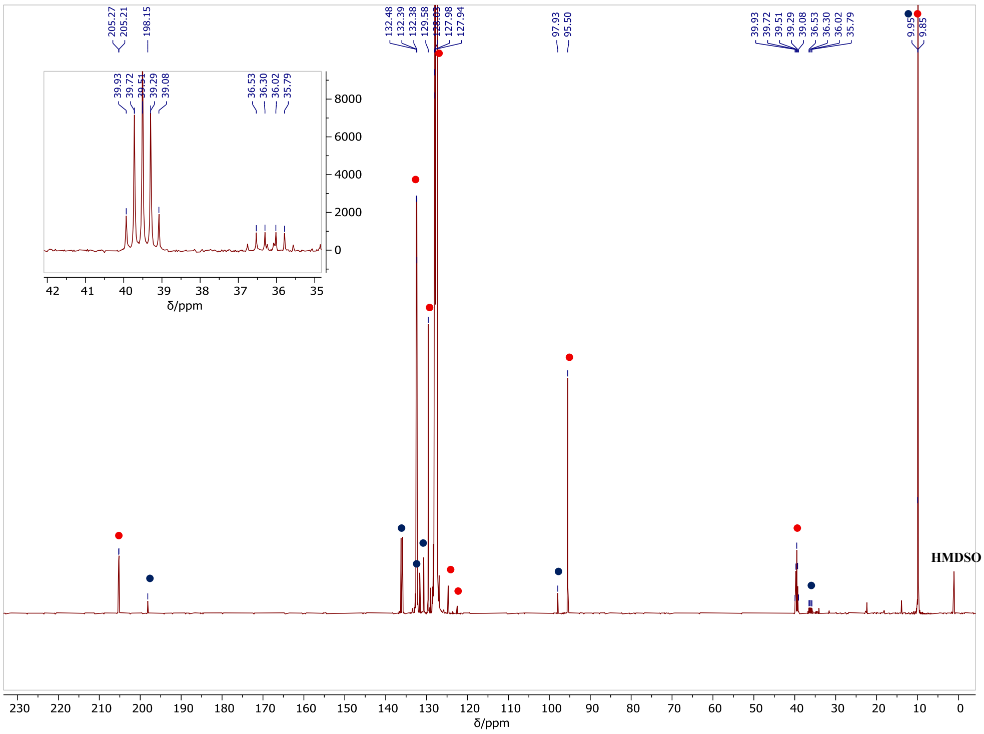

^{13}C NMR Spectra of **3c** (labelled in red), **3c'**(labelled in blue)

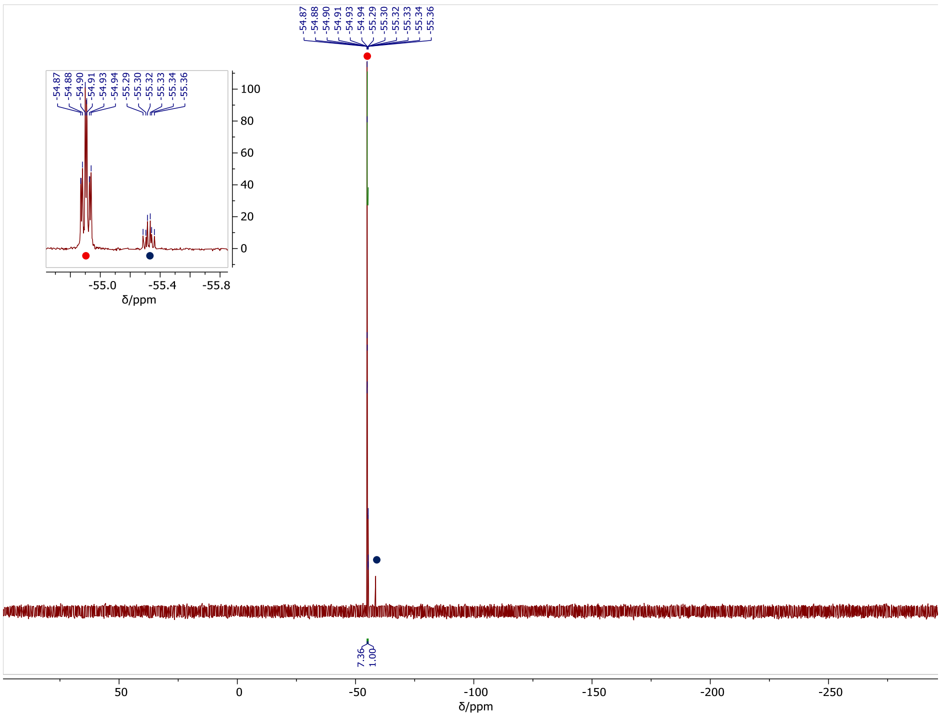

^{19}F NMR Spectra of **3c** (labelled in red), **3c'**(labelled in blue)

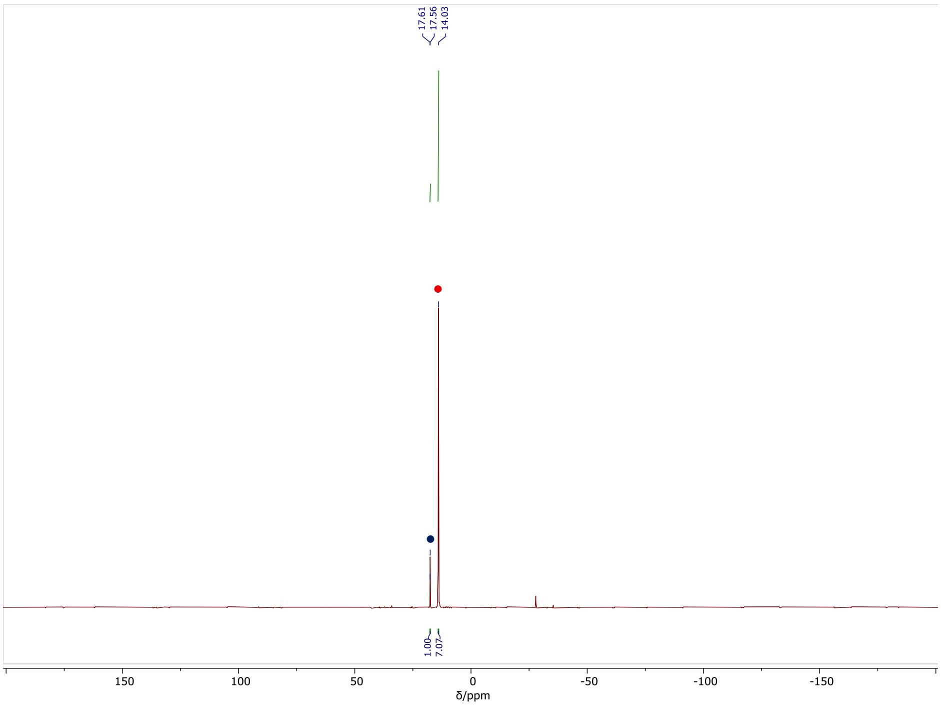

^{31}P NMR Spectra of **3c** (labelled in red), **3c'**(labelled in blue)

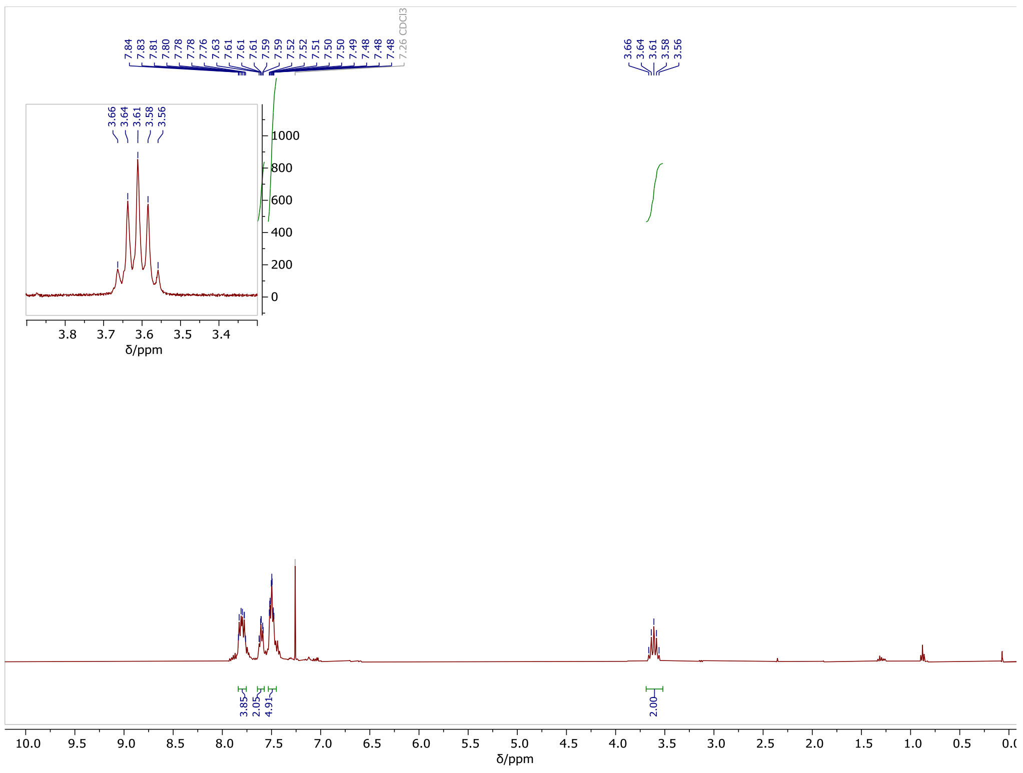

^{1}H NMR Spectra of **4**

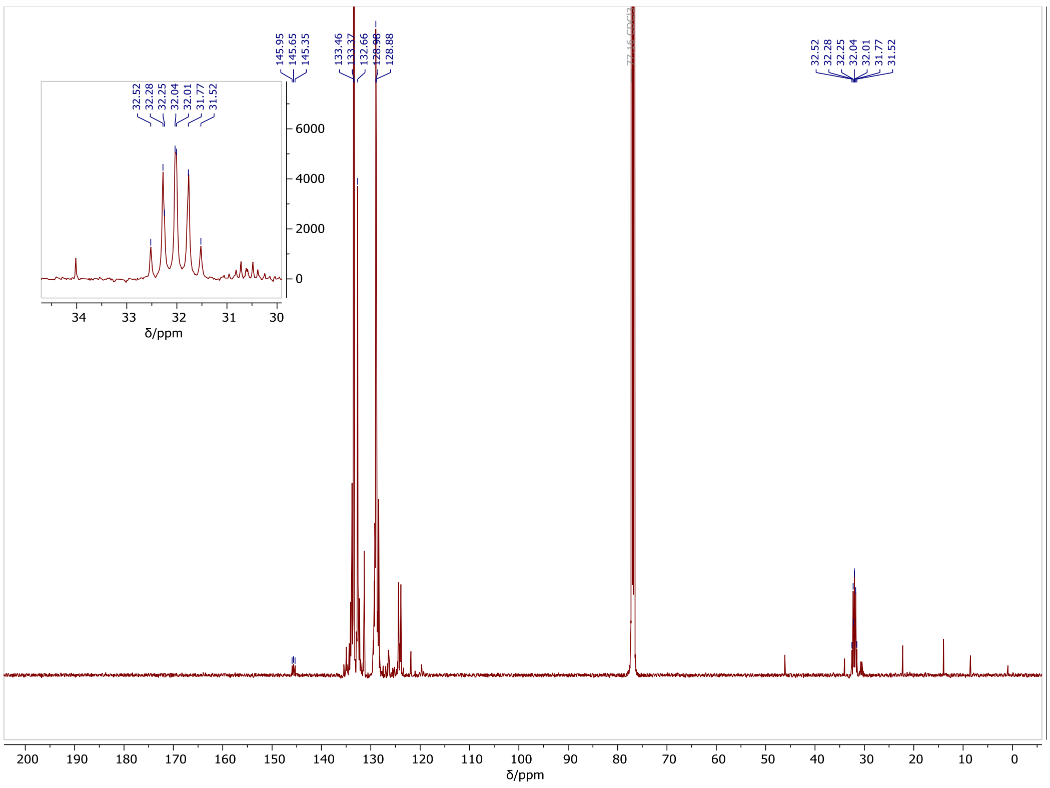

^{13}C NMR Spectra of **4**

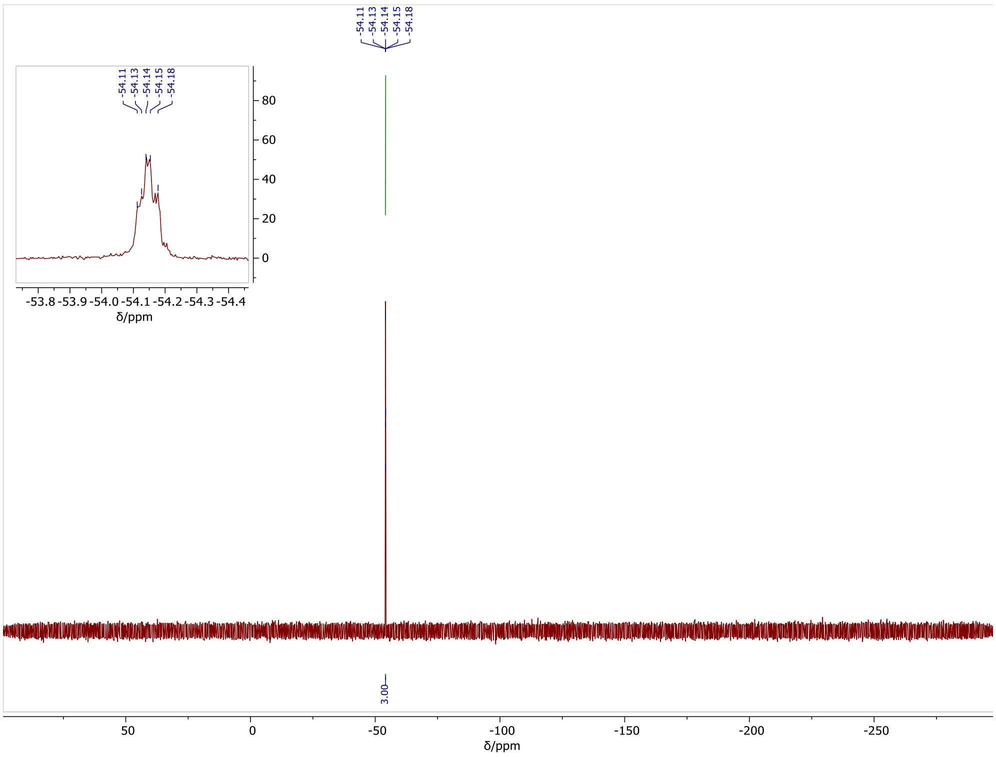

^{19}F NMR Spectra of **4**

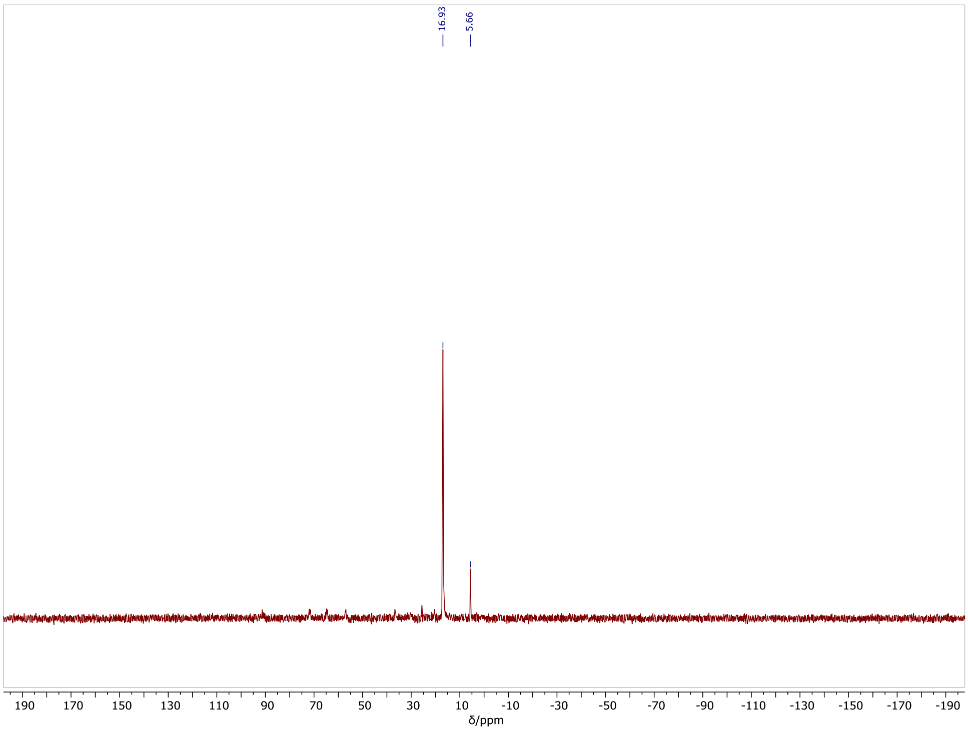

^{31}P NMR Spectra of **4**

11.1 References

1. J. Geicke, I. P. Lorenz and K. Polborn, *Inorg. Chim. Acta.*, 1998, **272**, 101-108.

2. I.-P. L. S.Rudolph, K.Polborn, *CSD Communication*, 2005.

3. G. M. H. Lang, O. Scheidsteger, G. Huttner, *Chem. Ber.*, 1985, **118**, 574.

4. S. Fortier, M. C. Baird, K. F. Preston, J. R. Morton, T. Ziegler, T. J. Jaeger, W. C. Watkins, J. H. MacNeil and K. A. Watson, *J. Am. Chem. Soc.*, 1991, **113**, 542-551.

5. L. Falivene, Z. Cao, A. Petta, L. Serra, A. Poater, R. Oliva, V. Scarano and L. Cavallo, *Nat. Chem.*, 2019, **11**, 872-879.

6. Agilent, CrysAlis PRO. Agilent Technologies Ltd, Yarnton, Oxfordshire, England).

7. O. V. Dolomanov, L. J. Bourhis, R. J. Gildea, J. A. K. Howard and H. Puschmann, *J. Appl. Crystallogr.*, 2009, **42**, 339-341.

8. G. Sheldrick, *Acta Crystallogr. Sect. A*, 2015, **71**, 3-8.

9. G. M. Sheldrick, *Acta Crystallogr. C Struct. Chem*, 2015, **71**, 3-8.

10. J. P. Perdew, J. A. Chevary, S. H. Vosko, K. A. Jackson, M. R. Pederson, D. J. Singh and C. Fiolhais, *Phys. Rev. B*, 1992, **46**, 6671-6687.

11. J. P. Perdew, J. A. Chevary, S. H. Vosko, K. A. Jackson, M. R. Pederson, D. J. Singh and C. Fiolhais, *Phys. Rev. B*, 1993, **48**, 4978-4978.

12. J. P. Perdew, K. Burke and M. Ernzerhof, *Phys. Rev. Lett.*, 1996, **77**, 3865-3868.

13. A. D. Becke, *J. Chem. Phys.*, 1993, **98**, 5648-5652.

14. M. J. Frisch, G. W. Trucks, H. B. Schlegel, G. E. Scuseria, M. A. Robb, J. R. Cheeseman, G. Scalmani, V. Barone, G. A. Petersson, H. Nakatsuji, X. Li, M. Caricato, A. V. Marenich, J. Bloino, B. G. Janesko, R. Gomperts, B. Mennucci, H. P. Hratchian, J. V. Ortiz, A. F. Izmaylov, J. L. Sonnenberg, Williams, F. Ding, F. Lipparini, F. Egidi, J. Goings, B. Peng, A. Petrone, T. Henderson, D. Ranasinghe, V. G. Zakrzewski, J. Gao, N. Rega, G. Zheng, W. Liang, M. Hada, M. Ehara, K. Toyota, R. Fukuda, J. Hasegawa, M. Ishida, T. Nakajima, Y. Honda, O. Kitao, H. Nakai, T. Vreven, K. Throssell, J. A. Montgomery Jr., J. E. Peralta, F. Ogliaro, M. J. Bearpark, J. J. Heyd, E. N. Brothers, K. N. Kudin, V. N. Staroverov, T. A. Keith, R. Kobayashi, J. Normand, K. Raghavachari, A. P. Rendell, J. C. Burant, S. S. Iyengar, J. Tomasi, M. Cossi, J. M. Millam, M. Klene, C. Adamo, R. Cammi, J. W. Ochterski, R. L. Martin, K. Morokuma, O. Farkas, J. B. Foresman and D. J. Fox, *Journal*, 2016.

15. J. Tomasi, B. Mennucci and R. Cammi, *Chem. Rev.*, 2005, **105**, 2999-3094.

16. S. Grimme, J. Antony, S. Ehrlich and H. Krieg, *J. Chem. Phys.*, 2010, **132**.

17. C. Dykstra, G. Frenking, K. Kim and G. Scuseria, 2005.

18. K. Fukui, *Acc. Chem. Res.*, 1981, **14**, 363-368.

19. G. Luchini, J. Alegre-Requena, I. Funes-Ardoiz and R. Paton, *F1000Research*, 2020, **9**.

20. I. M. Alecu, J. Zheng, Y. Zhao and D. G. Truhlar, *J. Chem. Theory Comput.*, 2010, **6**, 2872-2887.

21. S. Grimme, *Chem. Eur. J.*, 2012, **18**, 9955-9964.

22. S. Grimme, J. Antony, S. Ehrlich and H. Krieg, *J Chem Phys*, 2010, **132**, 154104.

23. E. D. Glendening, C. R. Landis and F. Weinhold, *J. Comput. Chem.*, 2013, **34**, 1429-1437.

24. F. Neese, *WIREs Comp. Mol. Sci.*, 2022, **12**, e1606.

25. F. Neese, *WIREs Comp. Mol. Sci.*, 2012, **2**, 73-78.

26. H. R. Warsame, S. L. Patrick, J. A. Bull, P. W. Miller and M. R. Crimmin, *Angew. Chem. Int. Ed.*, 2026, **65**, e16598.
